# Psilocybin with psychotherapeutic support for treatment-resistant depression: a pilot clinical trial

**DOI:** 10.1177/20451253251377187

**Published:** 2025-10-02

**Authors:** Sally Meikle, Olivia Carter, Paul Liknaitzky, Lauren Johansen, Ravi Iyer, Nigel Strauss, Martin Williams, David Castle, Susan L. Rossell

**Affiliations:** Melbourne School of Psychological Sciences, University of Melbourne, Redmond Barry Building, 17 Spencer Road, Melbourne, VIC 3010, Australia; Centre for Mental Health & Brain Sciences, Swinburne University of Technology, Melbourne, VIC, Australia; Melbourne School of Psychological Sciences, University of Melbourne, Melbourne, VIC, Australia; Department of Psychiatry, School of Clinical Sciences, Monash University, Melbourne, VIC, Australia; School of Psychological Sciences, Monash University, VIC, Australia; Centre for Mental Health & Brain Sciences, Swinburne University of Technology, Melbourne, VIC, Australia; Centre for Mental Health & Brain Sciences, Swinburne University of Technology, Melbourne, VIC, Australia; Centre for Mental Health & Brain Sciences, Swinburne University of Technology, Melbourne, VIC, Australia; Millswyn Clinic, South Yarra, VIC, Australia; Centre for Mental Health & Brain Sciences, Swinburne University of Technology, Melbourne, VIC, Australia; Psychedelic Research in Science & Medicine (PRISM) Ltd, Melbourne, VIC, Australia; Centre for Mental Health Services Innovation, University of Tasmania, Hobart, TAS, Australia; Centre for Mental Health & Brain Sciences, Swinburne University of Technology, Melbourne, VIC, Australia

**Keywords:** clinical trial, depression, psilocybin, psychedelic-assisted psychotherapy, qualitative

## Abstract

**Background:**

Depressive disorders are a major global health challenge, with many individuals unresponsive to existing treatments. Novel psychedelic therapies show promise but require further research.

**Objectives:**

This study aimed to evaluate the feasibility, safety and effectiveness of psilocybin with psychotherapeutic support for treatment-resistant depression (TRD), investigate predictors of treatment outcomes and deepen understanding of individual variability in response.

**Design:**

Open-label, single-arm pilot trial with mixed-methods assessment.

**Methods:**

Treatment consisted of two 25 mg psilocybin sessions, alongside three preparatory and six integration sessions. Depression severity was assessed using the self-rated Quick Inventory of Depressive Symptomatology at 3 weeks (primary endpoint) and at 20 weeks post-dose 2 (long-term follow-up). Potential predictors of clinical outcomes were evaluated using questionnaires, and qualitative interviews were used to capture individual experiences.

**Results:**

At the aggregate level, a clinically meaningful reduction in depressive symptoms was observed at the primary endpoint (mean change = –7.14; *p* = 0.02; Hedges’ g = –1.27; 95% CI [–2.40, –0.37]) and maintained long-term. Individual participant data revealed diverse response patterns. Two participants displayed a sustained treatment response, three relapsed, and two exhibited no substantial improvement. Exploratory analyses identified mindset prior to dosing, spiritual experiences and perceptual shifts during dosing as predictors of treatment trajectory, while treatment expectations were not a reliable predictor. Adverse events were largely consistent with previous studies, with no serious adverse events.

**Conclusion:**

Findings add to the growing evidence base for psilocybin therapy and provide direction for further research on individual variability in response to better tailor treatments and enhance efficacy.

**Trial registration:**

Australian New Zealand Clinical Trials Registry (ACTRN12621001097831).

## Plain language summary

Psilocybin therapy for depression: a small study on safety, effectiveness, and individual differences

Many people with depression do not respond to current treatments, leading researchers to explore new options like psilocybin, the active ingredient in psychedelic mushrooms. This study tested whether psilocybin, combined with therapy, could help people with treatment-resistant depression. Seven participants took part in two psilocybin sessions with supportive therapy. Overall, depression symptoms improved significantly. However, responses varied—some people had lasting benefits, while others saw their symptoms return or did not improve. Factors such as a person’s mindset before treatment and the nature of their experience during the sessions seemed to influence outcomes. The treatment was generally safe, with no serious side effects. These findings support further research to understand who benefits most from psilocybin therapy and how to optimise treatment.

## Introduction

Depressive disorders rank among the top three contributors to global non-fatal disease burden.^1,2^ Only around half of the patients respond to antidepressants^3^ or psychotherapy,^4^ and remission rates decrease with each progressive course of treatment.^5,6^ After two unsuccessful pharmacological interventions, the term ‘treatment-resistant depression’ (TRD) is often applied, with 10%–30% of those with major depressive disorder (MDD) falling into this category.^5,7^ TRD is associated with poorer clinical outcomes, greater healthcare utilisation, lower quality of life and occupational functioning and increased suicide risk.^5,8–11^ The pressing need for novel treatment approaches has fuelled a growing interest in psychedelic therapies.

Modern clinical trials have assessed the efficacy of psilocybin therapy in MDD and TRD populations, with over 500 participants across nine trials.^12–23^ All published studies reported significant improvements from baseline, and three showed a significantly larger improvement compared to a placebo group.^16,19,21^ However, one study, which used procedures to enhance blinding and minimise expectancy bias, failed to find superior effects of psilocybin compared to placebo.^20^ This raises the possibility that expectancy effects contribute to treatment outcomes. Furthermore, in a double-dummy blinded comparison trial, there was no significant difference in efficacy between psilocybin and 6 weeks of escitalopram.^13^ Response and remission rates at the primary endpoint varied widely across trials, ranging from 37% to 71% and 20% to 57%, respectively. Thus, while these results are promising, there remains uncertainty regarding the magnitude and consistency of effects.

This variability may stem from differences in trial design. While most studies in MDD and TRD populations have employed a single moderate-to-high dose of psilocybin, two have employed a two-dose protocol^13,15^ and reported higher response rates. A recent meta-analysis of the broader literature supports the superiority of two-dose protocols.^24^ Similarly, the number of non-dosing sessions may play a role in treatment efficacy and durability of treatment effects.

Research has established the physiological safety profile of psilocybin in healthy individuals^25^ but safety in clinical populations is still being characterised. Psychedelic therapy trials typically report mild, transient adverse events such as headache, nausea and anxiety. However, there have also been recent reports of suicidal ideation and self-injurious behaviour in the days and weeks following dosing.^16,19^ Additionally, there is growing recognition of unique interpersonal risks.^26,27^ Of particular interest is the use of therapeutic touch, viewed as clinically indicated in this modality^28^ yet generally outside the boundaries of conventional mental healthcare. Given the heightened vulnerability in these settings, the use of touch requires further empirical exploration.

How psychedelics such as psilocybin produce persisting effects has been the subject of much debate, with some prominent theories pointing to psychological mechanisms related to the acute experience. Findings show acute experiences, such as mystical experiences,^12,29–31^ psychological insight^30,32^ and emotional breakthrough^30,33–35^ may predict therapeutic benefit. There is also growing recognition that expectancy effects – a key component of placebo effects – shaped by positive media coverage,^36^ may contribute.^37,38^ Finally, while general preparedness for dosing has shown to predict the quality of experience,^39,40^ it is yet to be examined as a predictor of clinical benefit.

As psilocybin trials have scaled up, emphasis has shifted towards aggregate data to demonstrate overall efficacy. However, when individual-level data are reported, distinct response patterns emerge. Some participants experience sustained benefits, others relapse, some show no change or even worsen.^14,18,20^ Examining individual patterns of response across all timepoints could provide valuable information for tailoring therapies to individual needs.

Recently, the Australian Therapeutic Goods Administration (TGA) rescheduled psilocybin, allowing its prescription for TRD under specific circumstances.^41,42^ This presages an urgent need for research in the Australian context, along with developing the skills, infrastructure, and protocols for safe psychedelic therapy practices. The study described here aimed to address these needs by conducting a pilot, open-label trial with a two-dose psilocybin protocol combined with psychotherapeutic support for treatment-resistant depression. In addition to evaluating treatment effectiveness and safety, a mixed-methods approach was employed to better understand individual variability in treatment response. This included quantitative analyses to examine potential explanatory factors, alongside qualitative data and individual-level results to capture the nuanced experiences of participants. A fully disclosed, comprehensive treatment protocol is included to promote transparency and provide context for the findings.

## Methods

### Study design

The study was an open-label, single-arm, pilot trial of two 25 mg doses of psilocybin with psychotherapeutic support, conducted by a co-therapist dyad. All on-site study activities were conducted at Swinburne University.

All data were collected in accordance with approvals obtained from The Swinburne University Human Research Ethics Committee (#20231367-15628). The trial was conducted under the Clinical Trial Notification scheme (CT-2020-CTN-02260-1) and registered on the Australian New Zealand Clinical Trials Registry (ACTRN12621001097831). The investigational product was provided by Usona Institute (WI, USA). This manuscript was prepared in accordance with the TREND (Transparent Reporting of Evaluations with Nonrandomized Designs) statement. A completed TREND checklist is provided in the Supplemental Materials.

### Participants

This study aimed to recruit 15 participants through study advertisements, clinical trial registries, and direct referrals from health practitioners.

Inclusion criteria included age between 18 and 65, and moderate to severe Major Depressive Disorder (MDD) – Montgomery–Åsberg Depression Rating Scale (MADRS) score ⩾ 30, deemed treatment-resistant (defined as having failed to respond to two or more antidepressant medications in the current episode). Medical exclusion criteria included conditions and use of medications contraindicated for psilocybin administration, pregnancy or breastfeeding, and past 12-month use of psychedelic substances or 3,4-Methylenedioxymethamphetamine (MDMA) (or past 1-month for microdosing). Psychiatric exclusion criteria included very severe anxiety, depression or suicidality; current or past history of schizophrenia, other Psychotic Disorders or Bipolar I or II Disorder, or a first-degree relative with these conditions; current or past 5 year history with alcohol or drug dependence; current Dissociative Disorder, Anorexia Nervosa, Bulimia Nervosa, or any psychiatric condition judged by screening clinicians to be incompatible with the establishment of rapport or safe exposure to psilocybin; situational or personal factors that may interfere with involvement; or a ‘complex case’ of depression (e.g. extensive trauma history or complex psychiatric or medical comorbidities, or comorbidities where depression appears to be secondary).

Eligibility criteria were assessed in a four-phase screening process: (1) online pre-screening survey, (2) screening interview video-call, (3) medical exam, (4) consultation with treating healthcare professional. Following successful completion of all screening procedures, participants tapered off contraindicated medications under the supervision of their usual care team and were enrolled only after cessation of these agents.

### Trial intervention

**Figure 1.**
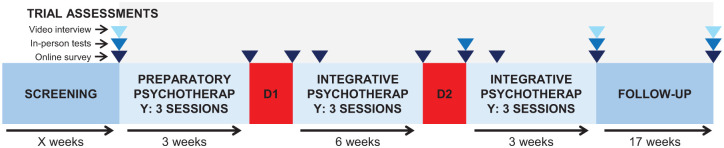
Timeline of trial intervention and assessments.

The intervention had three distinct phases:

(1) Preparation: Three 60–90 min psychotherapy sessions across 3 weeks, focused on building therapeutic alliance and preparing the participant for dosing.
(2) Dosing session: Two all-day sessions, 4–6 weeks apart. During each session, participants were encouraged to lie comfortably on a couch, wearing eyeshades and headphones playing preselected music, and attend to their internal experience. Participant-led, non-directive support was provided as needed. Minimal use of therapeutic touch was permitted within these sessions, subject to certain conditions. Prior to administration of psilocybin, a re-assessment of eligibility was conducted, which included blood pressure and heart rate measurements and drug and pregnancy urine tests. All dosing sessions were video recorded by a discreet camera.
(3) Integration: Three 60–90 min psychotherapy sessions following each dosing session, focused on exploring the dosing experience, integrating it into a wider context, and sustaining any positive changes (Figure 1).

The second dosing session was optional but encouraged unless contraindicated. All sessions were conducted by a co-therapy dyad. Therapists were qualified mental healthcare workers who underwent training designed specifically for this trial. The treatment protocol was not manualised, instead basic treatment guides were provided to therapists. The intervention was informed by contemporary practices in psychedelic therapy rather than a specific psychological framework. See Supplemental Methods for comprehensive information on trial screening and intervention.

### Trial assessments

The primary outcome was change in depression scores from baseline to 3 weeks post-dose 2, measured using the self-rated Quick Inventory of Depressive Symptomatology (QIDS-SR)^43^; Secondary health and well-being outcomes included the World Health Organization Quality of Life Questionnaire – Brief Version (WHOQoL-Bref)^44^; Generalized Anxiety Disorder 7-item scale (GAD-7)^45^; Brief Experiential Avoidance Questionnaire (BEAQ)^46^; two subscales from the Revised Self-Efficacy Scale (RSES)^47^; Watts Connectedness Scale (WCS)^48^ and the Persisting Effects Questionnaire (PEQ).^49^

Participants’ treatment expectations were assessed with the Patients’ Therapy Expectation and Evaluation questionnaire (PATHEV),^50^ containing three subscales (hope of improvement, fear of change, and suitability), administered at baseline and 1 day prior to dose 1. The Mindset questionnaire^40^ was used to assess participants’ preparedness, encompassing comfort in the treatment context, rapport with therapists, and intentions, administered on the morning of each dosing session. The 11 Dimension Altered States of Consciousness scale (11D-ASC),^51^ the Psychological Insight Questionnaire (PIQ)^52^ and Emotional Breakthrough Inventory (EBI)^33^ were administered the day following each dose.

Participants were asked about their experience of any adverse events (AEs) at all study visits. Blood pressure and heart rate were measured at minimum set points throughout each dosing session. A blood test was conducted at baseline and 1-day post-dose 1 to check participant health. At long-term follow-up, the Hallucinogen Persisting Perceptual Disorder (HPPD) Questionnaire^53^ was administered. Consent to, use of and participants’ experience of therapeutic touch were documented.

One-hour qualitative interviews were conducted via video call at baseline, 3 weeks post-dose 2 and long-term follow-up. For a full list of trial assessments, refer to the Supplemental Methods.

### Data analyses

Statistical analyses were performed using R Studio version 2024.4.1.748.^54^ Due to the small sample size, exact paired permutation tests were used to assess the effect of treatment on QIDS-SR scores and secondary outcome measures at the primary endpoint (3-week post-dose 2). Permutation tests offer robust results by relying on data-driven resampling to assess significance without making assumptions about the distribution of data.^55^ Effect sizes were calculated with Hedge’s g formula with 95% confidence intervals.

For comparison with previous research, the number of participants showing clinically-meaningful treatment response, defined as a reduction of 50% or more from baseline on QIDS-SR score,^56^ and remission, defined as a QIDS-SR score of 5 or less^57,58^ was calculated.

Upon data inspection, participants were grouped descriptively into different treatment trajectory categories based on their clinical outcomes throughout the study. Multinomial logistic regressions were run to test possible predictors of treatment trajectory: PATHEV subscales, 11D-ASC subscales, PIQ, EBI, and Mindset. Reference category was rotated to capture a fuller picture of the relationships among all outcome categories.

Due to the small sample size, analyses of secondary and exploratory outcomes were conducted using a descriptive approach, focusing on effect size estimation and confidence intervals rather than significance testing. Results with confidence intervals that did not cross zero (for Hedges’ g) or 1 (for odds ratios) were prioritised, and *p*-values are reported in the supplementary results for completeness. Quotes from qualitative interviews were selectively extracted to contextualise and complement quantitative findings.

## Results

### Participants

**Figure 2.**
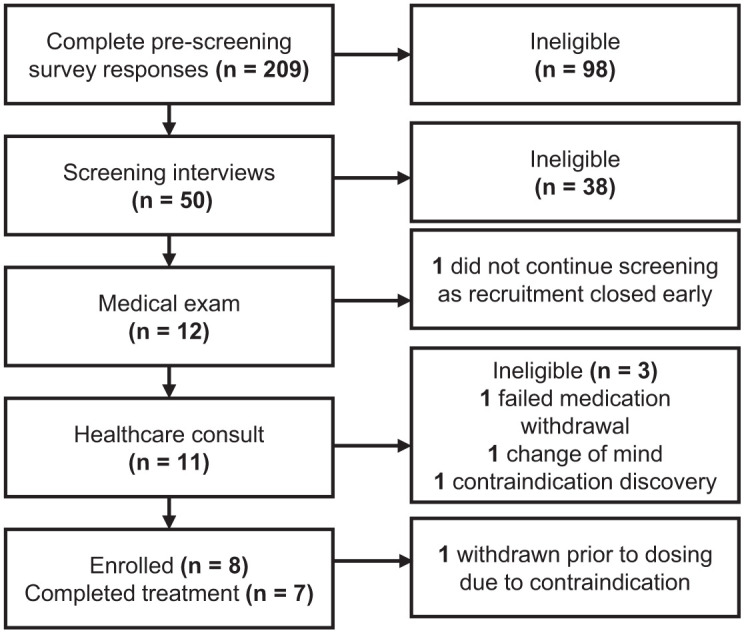
Screening and enrolment of participants.

Recruitment commenced in February 2022, with enrolment spanning August 2022 to August 2023. From an initial 209 complete pre-screening survey responses, eight participants were enrolled in the study (Figure 2). The most common reasons for exclusion at pre-screening were having taken a psychedelic drug in the previous 12 months (28.41%) and/or not meeting criteria for TRD (26.14%); and at interview, MADRS score < 30 (52.63%), and/or ‘complex case’ depression (34.21%). Of the eight enrolled; one was withdrawn prior to starting the treatment after discovery of factors leading to a ‘complex case’ of depression, determined to pose a safety concern (Table S1). The remaining seven participants completed the full treatment protocol and all outcome assessments reported here, and were included in all analyses. Due to delays related to the COVID-19 pandemic, and time and funding constraints, recruitment was closed prior to reaching the planned sample size. Final follow-up assessments were completed in January 2024.

The majority of potential and enrolled participants found out about the study from australianclinicaltrials.gov.au (49.03% and 75%, respectively) or a healthcare professional (18.93% and 25%, respectively; Table S2). Full demographic information for completing participants is displayed in Table 1.

**Table 1.** Participant demographics at screening.

Participant number	1	2	3	4	5	6	7
Sex	Male	Female	Male	Female	Female	Male	Female
Age	40–50	50–60	30–40	30–40	40–50	50–60	30–40
Employment	Unemployed	Part-time	Full-time	Full-time	Full-time	Full-time	Full-time
Marital status	Single	Married/de facto	Married/de facto	Single	Single	Married/de facto	Married/de facto
Estimated illness duration	30 years	30 years	5 years	17 years	30 years	45 years	5 years
Psychiatric comorbidities	ADHD	Nil	Nil	GAD	Nil	Nil	GAD
MADRS	34	34	30	32	30	31	30
QIDS-SR	13	17	14	16	8	10	15
Current episode number antidepressants	6	2	5	3	3	3	5
Lifetime number antidepressants	10	6	5	8	12	3	5
Previous talk-therapy: number therapists, total duration	2 therapists, 11 years	5 therapists, 5 years	2 therapists, 1 year	4 therapists, 17 years	3 therapists, 38 years	4 therapists, 2 years	1 therapist, <1 year
Other previous treatments	Ketamine, TMS	Nil	Nil	Nil	ECT, TMS	Ketamine	Nil
Previous psychedelic or MDMA use	20 occasions	5 occasions	Nil	1 occasion (microdose)	Nil	4 occasions	Nil
On antidepressants at screening	Yes	Yes	Yes	No	Yes	No	No

Demographics shown for completers only. Participants randomly labelled 1–9.

ADHD, attention deficit hyperactivity disorder; ECT, electroconvulsive therapy; GAD, generalised anxiety disorder; MADRS, Montgomery–Åsberg Depression Rating Scale; MDMA, 3,4-Methylenedioxymethamphetamine; QIDS-SR, quick inventory of depressive symptomatology (self-report); TMS, transcranial magnetic stimulation.

### Effectiveness

**Figure 3.**
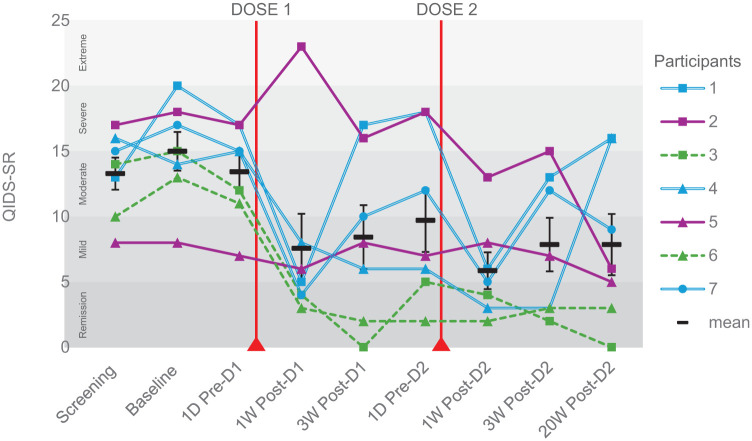
Change in QIDS-SR scores by participant. Depression severity over time for each of the seven participants with mean and standard error. Participants 3 and 6 = sustained response, participants 1, 4 and 7 = relapsing, participants 2 and 5 = non-response. Primary endpoint = 3-week post-dose 2. The response trajectory of participant 2 was challenging to categorise due to a lack of treatment response during the treatment period, followed by a reduction in QIDS-SR at long-term follow-up. This improvement cannot be clearly attributed, as the participant resumed antidepressant medication during the follow-up period. Participant 5 scored highly on the clinician-rated MADRS at screening, despite reporting low symptoms on the QIDS at the same timepoint and throughout the trial, suggesting possible underreporting on the self-report measure relative to clinician assessment. MADRS, Montgomery–Åsberg Depression Rating Scale; QIDS-SR, Quick Inventory of Depressive Symptomatology (Self-Report).

All participants displayed a reduction in QIDS-SR scores from baseline to the primary endpoint (3 weeks following dose two; Figure 3), with a significant mean reduction of 7.14 points (SD = 4.41; *p* = 0.02; Hedges’ g = –1.27, 95% CI [–2.40, –0.37]), and average decrease from baseline of 47.96%. Treatment response and remission was observed in three of seven participants at the primary endpoint (ID 3, 4 and 6). At the long-term follow-up (20-week post-dose 2), aggregated reduction in QIDS-SR scores was statistically significant (mean reduction of 7.14 points; SD = 5.84; *p* = 0.02; Hedge’s g = –1.16; 95% CI (–2.77, –0.46)). However, three participants resumed or started a new antidepressant medication prior to follow-up (ID 1, 2 and 5).

In terms of individual response trajectories, we determined three categories:

- Sustained response (two participants, ID 3 and 6): ⩾50% reduction in QIDS-SR scores from baseline at any point throughout the treatment (1-week post-dose 1 through to 3 weeks post-dose 2), sustained through to long-term follow-up.
- Relapsing (three participants, ID 1, 4 and 7): ⩾50% reduction in QIDS-SR scores from baseline at any point throughout the treatment but this reduction is not sustained through to long-term follow-up.
- Non-response (two participants, ID 2 and 5): <50% reduction in QIDS-SR scores from baseline at all points throughout the treatment.

### Secondary health and well-being outcomes

Mean improvements at the primary endpoint compared to baseline were observed in all secondary mental health and well-being outcomes (Table S3 and Figure S1), with all confidence intervals excluding zero, except for anxiety scores. The Persisting Effects Questionnaire scores indicated largely positive lasting changes to attitudes, mood and behaviour. Both QIDS scores and secondary health and well-being outcomes appeared to differ by treatment trajectory (Tables S4 and S5).

### Exploring indicators of clinical improvement

**Figure 4.**
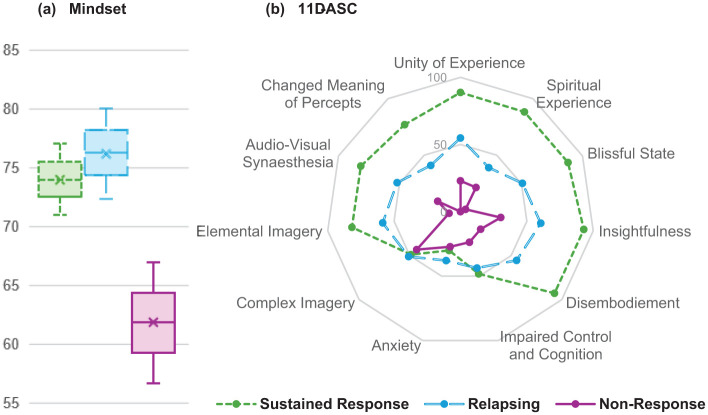
Mindset and 11D-ASC scores by treatment trajectory. Mean scores on (a) Mindset total scores and (b) 11D-ASC subscale scores for sustained response (*n* = 2), relapsing (*n* = 3), and non-response (*n* = 2) treatment trajectory groups. Mindset scores range from 0 to 100. 11D-ASC scores range from 0 to 100. 11D-ASC, 11 Dimension Altered States of Consciousness scale.

Multinomial logistic regression modelling showed that Mindset and some elements of the 11D-ASC reliably predicted treatment trajectory. Higher Spiritual Experience scores decreased the odds of relapsing and non-response compared to sustained response. Higher Changed Meaning of Percepts scores increased the odds of sustained and relapsing responses compared to non-response. Higher Mindset scores decreased the odds of non-response compared to both sustained and relapsing responses and increased the odds of sustained and relapsing responses compared to non-response. These models displayed good fit metrics (Table 2 and Figure 4). A combined multinomial logistic regression model was not run due to small sample size and multicollinearity between predictors. All other predictors displayed less certain effects (Table S6).

**Table 2.** Multinomial logistic regression models using the predictors mindset, spiritual experience and changed meaning of percepts.

Predictor	Reference	Outcome	OR (95% CI)	Model fit	
				Log-likelihood	McFadden’s *R*^2^
Mindset	Sustained	Non-response	0.13 (0.11, 0.15)	6.22	0.58
	Relapsing	Non-response	0.20 (0.17, 0.22)	6.38	0.58
	Non-response	Sustained	2.20 (1.62, 2.97)	6.93	0.54
		Relapsing	2.75 (2.10, 3.60)	–	–
11D-ASC SE	Sustained	Relapsing	0.44 (0.31, 0.63)	5.94	0.61
		Non-response	0.43 (0.30, 0.61)	–	–
11D-ASC CMP	Non-response	Sustained	106.70 (101.49, 113.30)	4.58	0.70
		Relapsing	101.49 (95.58, 105.64)	–	–

The large odds ratios for the 11D-ASC CMP subscale reflect complete separation, as all non-responders scored zero. In small samples, this can inflate ORs and produce confidence intervals that understate uncertainty.

11D-ASC CMP, 11 Dimension Altered States of Consciousness scale, Changed Meaning of Percepts subscale; 11D-ASC SE, 11 Dimension Altered States of Consciousness scale, Spiritual Experience subscale; CI, confidence interval; OR, odds ratio.

### Qualitative data

Inspection of qualitative data validated the categorisation of participants into treatment trajectory groups and revealed variations in acute subjective dosing experiences (Table 3).

**Table 3.** Participant quotes.

Treatment trajectory group	Acute subjective dosing experience	Changes to mental health and well-being following treatment
Non-response	*The first dose was to do with separation from, I think, my birth mother. So, there was a sense of just being separated, acute anxiety, a lot of crying and very intensely emotional. I found it really difficult. . . It was a bit visual, like a dark, kind of stormy setting. . . just feeling like I was being pulled away. . . and wanting to try and get back. . .* **Participant 2**	3W post-dose: *So both doses were quite traumatic and for the period since the first dose I had very high anxiety and have been quite depressed.* Long-term follow-up: I’m on more medication now. . . I feel marginally better than I did before starting the trial. I can’t really sort of evaluate what the trial has done. I guess. . . It’s shown me the underlying issues, but it wasn’t helpful in dealing with those issues. **Participant 2**
	*I haven’t had any. . . kind of out of this world, revelationary kind of experiences. . . it wasn’t necessarily a negative thing. I felt like I cried for 7 hours straight. It’s hard, I can’t even really identify what I necessarily remember or felt.* **Participant 5**	3W post-dose 2: *It’s been a hard few months. . . I think coming off the medication has been hard. I think a lot of the time I kind of operate a bit on automatic. . . so I’ve continued okay in life stuff.* Long-term follow-up: I really haven’t come out of the other side of it with any sort of great change to be honest. . . I think it certainly allowed me to get in touch with parts that I don’t normally kind of open up to. **Participant 5**
Relapsing response	*The first experience was characterised by a lot of beauty and safety. . . like being in the centre of a collage. . . a kind of construction of an external being who was. . . corralling all of these. . . very benevolent resources of nature. . . to come and tend to me. . . The second experience. . . was very, very different. . . I was very much in the room in my body. . . it was just an ordeal, like these constant waves of challenging energy. . . it involved a lot of. . . kind of growling and roaring and making a lot of noise.* **Participant 1**	3W post-dose 2: *The only real improvements have been during those short periods after trips.* Long-term follow-up: The psychedelic experience is this very significant spiritual/intellectual object or anchor in the world for me, it definitely shapes my perspective on things. . . but in terms of that following through into uplifting mood, it’s quite the opposite and it’s irritating to be honest, to have experienced some very profoundly caring loving spaces in psychedelics. . . the magic of all that just fails to make a difference, it’s disheartening. **Participant 1**
	*I. . . was going through different things in sort of primary school age. It was like really like re-experiencing those things and seeing them through a different sort of lens. . . I had all these thoughts about my parents and how difficult things must have been and from quite a compassionate forgiving light. And I literally got to the end of that was like “okay, what about me now?”* **Participant 4**	*One of the things I said to [my therapists] . . . was “I feel like I’ve made all this progress and I don’t want it to stop” . . . and then I felt this very sudden like.. this is maybe not continuing.* The first 6 weeks after the second dose I didn’t have any migraines which is unusual for me. . . maybe there’s been a bit less anxiety because I haven’t been biting my nails. . . that has continued still, even though my depression has come back. **Participant 4**
	*The first dose I just saw like really pretty colours and patterns. . . but yeah, nothing. . . no themes. My second dose was also slightly different. I remember feeling as I was looking down on my conflict, and I’m thinking “oh, I don’t want to be down there”, like that looks unpleasant.* **Participant 7**	3W post-dose: *Immediately after the doses there’d been calmness and yeah, stillness. . . and then. . . eventually went back into anxiety and sadness* Long-term follow-up: Still feeling fairly depressed. . . but from the anxiety side, I haven’t had an anxiety attack for I don’t even know how long. I feel that at work, I am better able to stay focused instead of having my brain going off in like 10 different directions. **Participant 7**
Sustained response	*All these different figures were moving like prisms, and like sacred, I guess, geometry, whilst my eyes were closed. . . the soundtrack really.. evokes like a response. . . it was almost like a pathway to access something inside of me. . . I remember hugging my parents and I was crying and that I had this feeling. . . that we’re all in in this together. . . and that was a massive release.* **Participant 3**	*I feel like a lot more positive about my life, and I feel like I’m looking forward to the future. I feel like I notice the beauty in things. . . I just generally feel a lot more connected to myself and to others. I’m alleviated of like, I guess a mental restraint. . . it feels like the sky has definitely cleared up.* **Participant 3**
	*I was completely dissolved. I had no idea of time, space, or anything. There was no memories, there was no visuals, no. It was all just feelings. . . I was like a child floating in the cosmos, and I was introduced to God. . . It was the most profound experience in my life. It was both the most blissful experience, but it was also the most terrifying experience.* **Participant 6**	*I haven’t felt depressed at all. I haven’t really felt anxious at all. I don’t expect never to feel depressed or anxious again. But I feel like instead of being up and down like that, it’ll be a more even keel. I feel like I can look at my transactional self and see it for what it is much clearer. I feel that it’s more removed from that part of the mind that has to negotiate the world, and I feel like there’s a higher aspect of my mind that has much more control over things.* **Participant 6**

Filler words and repetitions have been removed from quotes for readability. Quotes trimmed for brevity; ellipses indicate omitted words. All quotes taken from 3-week post-dose 2 and long-term follow-up interviews.

### Feasibility and participant safety and well-being

All participants received both doses of psilocybin. No serious adverse events occurred at any point during the trial (Table 4). See Tables S7 and S8 for full lists of adverse events.

**Table 4.** Adverse events during screening and trial.

Event	Screening *n* = 209	Trial *n* = 8
	Instances (number of participants)	
Any adverse event	4 (4)	27 (7)
Serious adverse event	0	0
All related adverse events	1 (1)	21 (6)
Related to antidepressant withdrawal (*n* = 5)	1 (1)	5 (2)
Depression (exacerbation)	1 (1)	2 (2)
Insomnia (exacerbation)	0	1 (1)
Insomnia	0	1 (1)
Sleep myoclonus (exacerbation)	0	1 (1)
Related to trial treatment	–	18 (6)
Abnormal perception (olfactory)	–	1 (1)
Anxiety	–	1 (1)
Decreased appetite	–	1 (1)
Headache	–	2 (2)
Inappropriate behaviour	–	2 (1)
Insomnia	–	1 (1)
Insomnia (exacerbation)	–	1 (1)
Nausea	–	6 (5)
Sleep myoclonus (exacerbation)	–	1 (1)
Suicidal ideation (exacerbation)	–	1 (1)
Urinary incontinence	–	1 (1)

AEs were not systematically assessed during screening. Some individual AEs are linked to both antidepressant withdrawal and trial treatment. Related adverse events here include those categorised as ‘possibly related’, ‘probably related’ and ‘related’.

AEs, adverse events.

One participant (ID 2) reported anxiety, insomnia and suicidal ideation following a very emotionally challenging first dose. Daily phone calls for 1 week from therapists were used to monitor well-being and provide support. This participant also received support from external mental healthcare providers and commenced psychiatric medications. These adverse events resolved by long-term follow-up, except for anxiety, which persisted and required ongoing treatment. A second participant (ID 5) received an additional integration session conducted via video call between doses, due to well-being concerns.

All participants consented to both participant- and therapist-initiated therapeutic touch, and five of the seven participants received touch. No changes to therapeutic touch consent were made between dose 1 and dose 2. All participants receiving touch reported it to be helpful.

## Discussion

This open-label pilot clinical trial examined the effectiveness, feasibility and safety of psilocybin with psychotherapeutic support for treatment-resistant depression (TRD). Consistent with prior research, a significant and clinically meaningful reduction in depressive symptoms was observed. Response and remission rates of 43% at the primary endpoint are comparable to other TRD psilocybin therapy studies. These results are particularly noteworthy given that for each participant, this was at least their third treatment attempt within the current depressive episode (and up to their seventh). Conventional treatments at this illness stage typically show response and remission rates of only 17% and 14%, respectively.^5,57^ Regarding long-term outcomes, sustained reductions in depression were observed at 20 weeks post-dose 2, in two participants. Previous research found a time to relapse of 11–20 weeks in similar populations, suggesting these results represent stable, enduring effects.^6^

The treatment protocol used here differed from previous research, incorporating two moderate-to-high doses and more intense preparation and integration.^59^ Although our results do not indicate that these modifications improved treatment outcomes, the small sample size and other differences make it difficult to assess their potential benefit.

Another factor to consider when interpreting these results is the use of the QIDS-SR as the primary outcome measure. While the QIDS-SR was selected for its brevity and use in similar trials,^12,13^ self-report measures are more susceptible to response bias, and tend to show greater improvements than clinician-rated measures.^60^ Also, concerns have been raised regarding the suitability of the QIDS-SR for psychedelic research specifically.^61^

Finally, over half of the participants reported lifetime use of a psychedelic or MDMA. Although these instances were not recent, this proportion is higher than those in previous trials and in the general population.^62^ This is noteworthy given recent meta-analytic findings that a higher proportion of participants with prior psychedelic experience is associated with more favourable outcomes following psilocybin therapy,^60^ possibly due to positive expectancy and greater familiarity, which can limit adverse outcomes. Future research could address this issue using a masked recruitment strategy or excluding those with prior psilocybin use.

### Exploratory prediction of treatment trajectory

While response and remission rates at the primary endpoint facilitate comparison with prior studies, categorising participants by overall treatment trajectory provided a fuller picture of response patterns. Two participants showed a sustained response, three experienced relapse after initial improvement and two had no substantial symptom reduction. These distinct patterns were linked to lasting changes in attitude, mood and behaviour, highlighting the importance of understanding patterns of individual responses.

Mindset prior to dosing and aspects of the acute psychedelic experience predicted treatment trajectory, with the latter supported by qualitative data. Our findings suggest that psychological readiness, comfort, rapport and clear intention before dosing (mindset), combined with emotionally intensified, novel or meaningful perceptual changes during dosing, are associated with an initial positive response to psilocybin therapy for TRD. In contrast, a sustained response appears linked to profound spiritual experiences during dosing.

This link between spiritual experience and benefit aligns with previous research,^63^ although other dimensions related to the mystical experience, such as unity of experience or insightfulness, did not reliably predict treatment outcomes in this study. Changed Meaning of Percepts emerged as a novel predictor, supporting the view that therapeutic effects of psychedelics may stem from emotional insights or perspective shifts that come from processing experiences in new ways. The link between Mindset and clinical benefit may occur through these or other changes to acute subjective experience.^39,40^ Subject to confirmation in future research, this finding supports screening of participants prior to dosing to ascertain their level of preparedness and providing additional preparation when indicated.

Treatment expectations did not emerge as a consistent predictor of treatment trajectory. However, positive expectations have been associated with improved outcomes in psychotherapy^64^ and with conventional antidepressant treatments.^65^ In this study, the lack of association may reflect limited variability in expectancy scores, with some subscales showing consistently high ratings across participants. While a prior study^66^ has also reported expectations lacked predictive value, a non-standardised, single-question measure was used. Further research is needed to clarify the relationship between expectations and treatment outcomes and to identify or develop tools capable of capturing meaningful variability in expectancy within this context. All interpretations from the current study regarding exploratory predictors should be considered tentative due to the small sample size.

### Safety, well-being and feasibility

Documented adverse events were largely congruent with previous research. Additional support outside of pre-scheduled trial treatment sessions was required for two participants, highlighting the need for flexibility in trial design and the ability to respond to individual participant needs. Transient suicidal ideation recorded here adds to the emerging understanding of suicidality as a risk in psychedelic trials.^16,19^ Furthermore, persisting anxiety reported by one participant underscores the importance of addressing the potential for long-term adverse psychological reactions following psilocybin therapy in future studies and the need for more consistent reporting on the duration and resolution of adverse events.

This trial monitored AEs in a similar manner to previous trials – using open-ended questions rather than structured questionnaires, and not documenting expected acute effects of psilocybin (e.g. changes in visual perception) as AEs; these methods likely result in underreporting of AEs.^67^ Furthermore, it has been argued that standard monitoring methods may not adequately capture risks specific to psychedelic therapy, such as sociocultural and interpersonal events.^27,68,69^ While this trial used additional questionnaires to assess interpersonal risks related to therapeutic touch, finding no adverse events, future research would benefit from the use of standardised tools such as those recently developed to assess psychedelic-specific harms.^68,70^

These results highlight challenges with antidepressant discontinuation, including withdrawal-related AEs and increased QIDS-SR scores from screening to baseline, likely due to symptom exacerbation during withdrawal, which may inflate baseline scores.^71^ This is important to consider when interpreting symptom change across the trial, as some apparent improvement may reflect the natural resolution of withdrawal effects over time rather than true treatment-related change. While a longer washout period might mitigate this, it is often not feasible due to individuals’ reluctance to remain without treatment. Discontinuation is recommended due to safety concerns and potential dampening of psychedelic effects,^72,73^ however, preliminary findings suggest acute subjective effects,^23,74^ safety and therapeutic outcomes^23^ may remain intact when antidepressants are continued during psilocybin therapy, challenging the necessity of this protocol.

### Limitations

Beyond the small sample size, reliance on a single self-report depression measure and self-referral recruitment, the study’s conclusions are limited by the open-label design. Double-blind randomised controlled trials are the gold standard for efficacy, though psychedelic therapy complicates this design due to functional unblinding. Active placebos and incomplete disclosure have been suggested to address this, though questions remain regarding their effectiveness.^75^ Combining and modifying these methods in novel trial designs may help reduce participant certainty about treatment allocation.^38,76^

A further important limitation of this study is the extensive, highly selective exclusion criteria. Individuals presenting with a variety of comorbid psychiatric conditions, complex clinical profiles or traits considered likely to interfere with the development of therapeutic rapport were excluded from participation. Although this approach is consistent with prior psychedelic research protocols,^14^ it limits the extent to which the findings can be generalised to broader clinical populations, where such complexities are frequently encountered.

## Conclusion

This study contributes to the emerging picture of the feasibility, safety and effectiveness of psilocybin with psychotherapeutic support for treatment-resistant depression. Conducted in an Australian setting, this trial mirrors findings in the USA and Europe, serving as an important replication of previous research with varying contextual factors. A comprehensive Supplemental Method is provided to ensure transparency and reproducibility, detailing a dual-dose approach with extensive preparatory and integration support. Substantial reductions in depressive symptoms were observed, with some participants maintaining these effects at 20 weeks post-treatment, suggesting potential for sustained benefit. Our exploration of individual treatment trajectories revealed possible predictors of therapeutic outcomes, identifying mindset prior to dosing and altered perceptions of meaning during acute dosing as contributors to initial response, and spiritual experiences during acute dosing as a contributor to sustained benefit. The case-series approach facilitates closer examination of patterns within the data, providing a greater depth of understanding of individual treatment trajectories.

## Supplemental Material

sj-docx-1-tpp-10.1177_20451253251377187 – Supplemental material for Psilocybin with psychotherapeutic support for treatment-resistant depression: a pilot clinical trial Supplemental material, sj-docx-1-tpp-10.1177_20451253251377187 for Psilocybin with psychotherapeutic support for treatment-resistant depression: a pilot clinical trial by Sally Meikle, Olivia Carter, Paul Liknaitzky, Lauren Johansen, Ravi Iyer, Nigel Strauss, Martin Williams, David Castle and Susan L. Rossell in Therapeutic Advances in Psychopharmacology

sj-docx-2-tpp-10.1177_20451253251377187 – Supplemental material for Psilocybin with psychotherapeutic support for treatment-resistant depression: a pilot clinical trial Supplemental material, sj-docx-2-tpp-10.1177_20451253251377187 for Psilocybin with psychotherapeutic support for treatment-resistant depression: a pilot clinical trial by Sally Meikle, Olivia Carter, Paul Liknaitzky, Lauren Johansen, Ravi Iyer, Nigel Strauss, Martin Williams, David Castle and Susan L. Rossell in Therapeutic Advances in Psychopharmacology

sj-docx-3-tpp-10.1177_20451253251377187 – Supplemental material for Psilocybin with psychotherapeutic support for treatment-resistant depression: a pilot clinical trial Supplemental material, sj-docx-3-tpp-10.1177_20451253251377187 for Psilocybin with psychotherapeutic support for treatment-resistant depression: a pilot clinical trial by Sally Meikle, Olivia Carter, Paul Liknaitzky, Lauren Johansen, Ravi Iyer, Nigel Strauss, Martin Williams, David Castle and Susan L. Rossell in Therapeutic Advances in Psychopharmacology

## Declarations

**Ethics approval and consent to participate:** All data were collected in accordance with approvals obtained from The Swinburne University Human Research Ethics Committee (#20231367-15628). All participants completing the screening survey were provided with the Participant Information and Consent Form, and consent was implied by the completion of the survey. At the next stage of screening, a formal written consent process is completed with study staff.

**Consent for publication:** Written informed consent to publish this data was obtained from all participants for whom individual-level data are presented in this submission.

**Competing interests:** SM declared no potential conflicts of interest with respect to the research, authorship and/or publication of this article. OC has received funding from The Perception Restoration Foundation. The organisation was not involved in any aspect of this paper, including the decision to write it, drafting the paper or its publication. PL has received research funding from Incannex Healthcare Ltd, the Multidisciplinary Association for Psychedelic Studies, and Beckley Psytech, is a member of the Medical Advisory Board of Incannex Healthcare Ltd, the Scientific Advisory Board of the MIND Foundation and a co-founder of Clarion Clinics. These organisations were not involved in any aspect of this paper, including the decision to write it, drafting the paper or its publication. LJ declared no potential conflicts of interest with respect to the research, authorship, and/or publication of this article. RI declared no potential conflicts of interest with respect to the research, authorship, and/or publication of this article. NS declared no potential conflicts of interest with respect to the research, authorship, and/or publication of this article. MW is Executive Director of the Australian charity, Psychedelic Research in Science & Medicine, Ltd. The organisation was not involved in any aspect of this paper, including the decision to write it, drafting the paper, or its publication. DC has received grant monies for research from NHMRC, MRFF, Barbara Dicker Foundation, CIHR (Canada), Brain Canada, Eli Lilly, Janssen Cilag, Roche, Allergen, Bristol-Myers Squibb, Pfizer, Lundbeck, Astra Zeneca, Hospira, Boehringer Ingelheim, iNova; Travel Support and Honoraria for Talks and Consultancy from and is a past Advisory Board Member for Lu AA21004: Lundbeck; Varenicline: Pfizer; Asenapine: Lundbeck; Aripiprazole LAI: Lundbeck; Lisdexamfetamine: Shire; Lurasidone: Servier; Brexpiprazole: Lundbeck; Treatment Resistant Depression: LivaNova; Cariprazine: Seqirus; Boeringher Ingelheim: Cognition in Schizophrenia; Risvan: Servier. He is a founder of the Optimal Health Program (OHP) and holds 50% of the IP for OHP; and is part owner (8%) of Clarity Healthcare. DC receives royalties for books from Allen and Unwin, Elsevier, Oxford University Press and Cambridge University Press. DC is an Advisory Board Chair of Psychae, a not-for-profit institute specialising in psychedelic medicines research; and is an advisor to Tryp, a for-profit psychedelics company. He does not knowingly have stocks or shares in any pharmaceutical company. The organisations were not involved in any aspect of this paper, including the decision to write it, drafting the paper, or its publication. SR is on the advisory board of Psychae Therapeutics, a commercial company developing psychedelic therapies and has received funding from Woke Pharmaceuticals for another psilocybin-assisted psychotherapy trial. She is also on the advisory board of Boehringer Ingelheim Ltd. These organisations were not involved in any aspect of this paper, including the decision to write it, drafting the paper, or its publication.

**Availability of data and materials:** De-identified individual participant data will be made available on a case-by-case basis at the discretion of the Coordinating Principal Investigator, Susan Rossell. Data will be available for all study outcomes; however, some demographic information may be withheld to protect participant confidentiality given the small sample size.
