# Supplementary material for: Psilocybin with psychotherapeutic support for treatment-resistant depression: a pilot clinical trial
Source: Ther Adv Psychopharmacol. 2025 Oct 2;15:20451253251377187. doi: 10.1177/20451253251377187 (PMC12491823; doi:10.1177/20451253251377187)
Supplement: sj-docx-1-tpp-10.1177_20451253251377187 – Supplemental material for Psilocybin with psychotherapeutic support for treatment-resistant depression: a pilot clinical trial [file sj-docx-1-tpp-10.1177_20451253251377187.docx]

**Supplementary Methods**

**Psilocybin with Psychotherapeutic Support for Treatment-Resistant Depression: A Pilot Clinical Trial (PsiloTRD)**

Contents

[Study Overview 5](#_Toc179628494)

[Rationale 5](#_Toc179628495)

[Study design 5](#_Toc179628496)

[Research objectives 5](#_Toc179628497)

[Primary objectives 5](#_Toc179628498)

[Secondary objectives 5](#_Toc179628499)

[Exploratory objectives 6](#_Toc179628500)

[Recruitment and screening 6](#_Toc179628501)

[Sample size 6](#_Toc179628502)

[Recruitment 6](#_Toc179628503)

[Screening procedures 6](#_Toc179628504)

[Screening survey (online) 6](#_Toc179628505)

[Screening interview (video-call) 6](#_Toc179628506)

[Medical exam (in-person) 7](#_Toc179628507)

[Treating healthcare professional consultation and antidepressant withdrawal 7](#_Toc179628508)

[Screening assessments 7](#_Toc179628509)

[Eligibility criteria 8](#_Toc179628510)

[Inclusion criteria 8](#_Toc179628511)

[Medical exclusion criteria 9](#_Toc179628512)

[Psychiatric exclusion criteria 13](#_Toc179628513)

[Informed consent 15](#_Toc179628514)

[Trial Intervention 15](#_Toc179628515)

[Treatment overview 15](#_Toc179628516)

[Therapists and training 16](#_Toc179628517)

[Preparatory psychotherapy sessions 17](#_Toc179628518)

[Session 1: The participant and the approach 17](#_Toc179628519)

[Session 2: The psychedelic experience and tool for navigating it 18](#_Toc179628520)

[Session 3: Outline dosing day practicalities 19](#_Toc179628521)

[Dosing sessions 19](#_Toc179628522)

[Psilocybin 19](#_Toc179628523)

[Dosing session procedures 19](#_Toc179628524)

[Vital signs monitoring 20](#_Toc179628525)

[Rescue medications 21](#_Toc179628526)

[Video recording 21](#_Toc179628527)

[Setting 21](#_Toc179628528)

[Music 22](#_Toc179628529)

[Therapeutic touch 23](#_Toc179628530)

[Support person 24](#_Toc179628531)

[Integrative psychotherapy sessions 24](#_Toc179628532)

[Monitoring and reviewing eligibility 25](#_Toc179628533)

[Participant information and instructions 26](#_Toc179628534)

[Additional support during the treatment phase 27](#_Toc179628535)

[Post-treatment phase support 27](#_Toc179628536)

[Participant reimbursement 27](#_Toc179628537)

[Trial Assessments 27](#_Toc179628538)

[Schedule 27](#_Toc179628539)

[Measures 31](#_Toc179628540)

[Primary Measures 31](#_Toc179628541)

[Secondary Measures 31](#_Toc179628542)

[Safety and Ethics 36](#_Toc179628543)

[Safety measures 36](#_Toc179628544)

[Adverse events 37](#_Toc179628545)

[Definitions 37](#_Toc179628546)

[Reporting procedures 39](#_Toc179628547)

[Follow-up of AEs 40](#_Toc179628548)

[Risks and risk mitigation 40](#_Toc179628549)

[Psychological risks 40](#_Toc179628550)

[Cardiovascular and sympathomimetic effects 41](#_Toc179628551)

[Reproductive and Developmental Risks 42](#_Toc179628552)

[Drug Dependence and Abuse Potential 42](#_Toc179628553)

[Therapeutic boundaries 42](#_Toc179628554)

[Data management 42](#_Toc179628555)

[Data collection and secure storage 42](#_Toc179628556)

[Monitoring, audits and documentation 43](#_Toc179628557)

[Data Entry and Tracking 43](#_Toc179628558)

[Protocol Deviations 43](#_Toc179628559)

[Monitoring 44](#_Toc179628560)

[Audits 44](#_Toc179628561)

[Reporting 44](#_Toc179628562)

[Data disposal 44](#_Toc179628563)

[Data Safety Monitoring Board (DSMB) 44](#_Toc179628564)

[Ethical approval 45](#_Toc179628565)

[References 45](#_Toc179628566)

# Study Overview

## Rationale

Major depressive disorder (MDD) poses a major unmet clinical need as one of the most prevalent and debilitating mental health disorders worldwide. Although treatments are available, such as antidepressant medications, only approximately 50% of patients see adequate improvements (Cipriani et al., 2018; Undurraga & Baldessarini, 2012). There is an urgent need for novel interventions to improve outcomes for people suffering with MDD, particularly in treatment-resistant cases.

Psilocybin therapy is a novel, experimental intervention for a variety of mental health conditions that has shown promising results in a small number of research trials conducted outside of Australia. This pilot trial investigated whether psilocybin with psychotherapeutic support improves the symptoms of MDD, as well as assess the feasibility of this research in an Australian context. Additionally, the study explored potential mechanisms of psilocybin therapy.

## Study design

The study was an open-label, single-arm, pilot trial. Participants with treatment-resistant depression were recruited. Participants received two 25 mg doses of psilocybin (second dose optional) with psychotherapeutic support, 6 weeks apart, accompanied by preparatory and integration psychotherapy sessions, all conducted by a co-therapist dyad. The primary endpoints were 3 weeks and 20 weeks following the second dose.

## Research objectives

### Primary objectives

- To determine whether psilocybin with psychotherapeutic support significantly reduces depressive symptoms.
- To determine whether any changes in depression symptoms are maintained at long-term follow-up.

### Secondary objectives

- To determine whether participants’ quality of life and symptoms of anxiety are impacted by psilocybin with psychotherapeutic support.
- To determine how certain aspects of the acute psilocybin experience relate to changes in depressive symptoms.
- To measure safety and feasibility outcomes for psilocybin with psychotherapeutic support (recruitment and retention rates as well as examining how many participants agree to the second dosing session).

### Exploratory objectives

- To explore participants’ subjective experience and perspectives about their depression pre- and post-treatment and examine how psilocybin with psychotherapeutic support may have impacted them using qualitative assessment.

## Sponsor

Investigator-initiated - Professor Susan Rossell, srossell@swin.edu.au.

## Funding

Funding provided by Barbara Dicker Brain Sciences Foundation and Usona Institute (provision of study drug only). Funders had no role in study design, data collection, or publication of results.

## Trial registration

This trial will be conducted under the Clinical Trial Notification (CTN) scheme and registered on Australian New Zealand Clinical Trials Registry (ANZCTR).

## Trial sites

Centre for Mental Health, School of Health Sciences, Swinburne University, John St, Hawthorn, Vic 3122.

# Recruitment and screening

## Sample size

We aimed to enrol 15 participants in this study. The target sample size was based upon the primary outcome measure of QIDS score. Using QIDS as the primary outcome, Carhart-Harris and colleagues (2018) established marked reductions in depressive symptoms at 1-week (Cohen's *d* = 2.2) and 5-weeks post treatment (Cohen's *d* = 2.3). Using an effect size of *d* = 2 and 1% significance will require *n* = 9 completers at 20 weeks post-dose 2 to achieve 95% power. This calculation was for a single-arm design (2-sided) assuming a Wilcoxon Signed Rank Test.

## Recruitment

Participants were recruited using several methods. Study advertisements (see Appendix A) and information were placed on social media, at university campuses, and on clinical trial registries (trial ID: ACTRN12621001097831). Information regarding the trial was also provided to interested medical practitioners and therapists (see Appendix B). Additionally, information was sent directly to a mailing list of individuals interested in psychedelic therapy, provided by the non-profit research charity PRISM (Psychedelic Research in Science and Medicine, Ltd).

## Screening procedures

### Screening survey (online)

All individuals interested in participating were provided with a link to the participant information and consent form, and an online screening survey. This survey asked participants for their contact details and briefly assessed key eligibility criteria using self-report questions. Ineligible participants were informed via email and were provided with relevant mental health resources. Those who were potentially eligible were informed that there were additional screening assessments (as below).

### Screening interview (video-call)

Interviews were conducted via zoom. At the start of the interview, verbal and written consent will be obtained as well as consent to record the screening interview. All eligibility criteria were assessed with custom self-report questions and standardised assessments (Drug Abuse Screening Test, DAST-10; Alcohol Use Disorders Identification Test, AUDIT; Level of Personality Functioning Scale – Brief Form 2.0, LPFS-BF 2.0; Adverse Childhood Experiences scale, ACE; Quick Inventory of Depressive Symptomatology – Self Report, QIDS-SR) and a structured clinical interviews (Montgomery–Åsberg Depression Rating Scale, MADRS; Diagnostic Interview for Anxiety, Mood, OCD and related Neuropsychiatric Disorders, DIAMOND). Interviews took approximately 2 hours. Interviews were terminated early if a participant clearly did not meet eligibility criteria. Results were reviewed by a screening review panel, including the interviewer, the trial psychiatrist, the coordinating principal investigator and the trial coordinator.

### Medical exam (in-person)

Participants determined to be potentially eligible after the screening interview were invited to a screening medical exam conducted by the trial nurse at Swinburne University. The following assessments were conducted: height; weight; body temperature, 12-lead electrocardiogram (ECG), blood pressure (x2), heart rate (x2), and a blood sample. The blood sample was used to run a full blood examination, liver, kidney and thyroid tests.

### Treating healthcare professional consultation and antidepressant withdrawal

Participants determined to be eligible after the screening medical exam were contacted and asked to consult with their treating mental healthcare professional about their possible participation. For participants taking antidepressant medications consultation with their prescribing physician occurred to taper them off their contraindicated medications under supervision. Once contraindicated antidepressant medications had been ceased, participants were enrolled in the trial.

## Screening assessments

- Diagnostic Interview for Anxiety, Mood, and OCD and related Neuropsychiatric Disorders (DIAMOND; Tolin et al., 2016). The DIAMOND is a semi-structured clinical interview designed to diagnose as assess a range of DSM-5 disorders. The interview was conducted by a trained clinician and consists first of screening questions to detect possible disorders, with follow-up questions confirming diagnosis and assessing severity.
- Montgomery–Åsberg Depression Rating Scale (MADRS; Montgomery & Åsberg, 1979). The MADRS is a clinician rated measure of severity of depressive symptoms. It consists of 10 items rated from 0 to 6.
- The Drug Abuse Screening Test (DAST-10; Skinner, 1982). This widely used measure consists of 10 items to which participants circle ‘yes’ or ‘no’ and screens for drug use problems, excluding alcohol.
- Alcohol Use Disorder Identification Test (AUDIT; Saunders, Aasland, Babor, De la Fuente, & Grant, 1993). This widely used measure consists of 10 self-rated items and screens for alcohol use problems.
- Adverse Childhood Events (ACE; Felitti et al., 1998) scale. This widely used measured consists of 10 items, each one an adverse childhood experience, to which the individual is asked to indicate if this happened to them or not. The ACE will be used to assess the extent of childhood trauma.
- Level of Personality Functioning Scale-Brief Form 2.0 (LPFS-BF 2.0; Weekers, Hutsebaut, & Kamphuis, 2019). This 12-item self-report questionnaire measures self- and interpersonal-functioning and is used to screen for possible personality disorders.
- 16-item Quick Inventory of Depressive Symptomatology – Self-rated (QIDS; Rush et al., 2003). The QIDS is a self-rated measure of depression that offers the advantages of brevity, increasingly widespread use, and validity at 1-week intervals. This measure assesses nine domains: sad mood, concentration, self-outlook, suicidal ideation, involvement, energy/fatiguability, sleep disturbance, appetite/weight increase/decrease, and psychomotor agitation/retardation. It consists of 16 items rated from 0-3. This scale will be used as the primary outcome and is administered at screening to allow for tracking of changes in symptoms of depression between screening and baseline assessment timepoints.
- Custom questions. A wide range of custom questions will be used to assess screening criteria in both the screening survey and interview.

## Eligibility criteria

### Inclusion criteria

Table 1. Inclusion criteria

| **Criteria** | **Method for assessment and cut-offs** |
| --- | --- |
| Aged 18-65 | Self-report |
| Proficient in English | Self-report Screening staff judgement during screening interview |
| Lives in Victoria | Self-report |
| Experiencing severe unipolar depression | MADRS (score equal to or above 30) |
| Under the care of a psychiatrist, psychologist, GP or other healthcare professional | Self-report and confirmed by treating healthcare professional |
| Diagnosed with major depressive disorder | Self-report previous diagnosis  Diagnosis confirmed by the screening doctor using the DIAMOND and other relevant information during the screening interview |
| Depression is ‘treatment-resistant’. Depressive symptoms have not responded to two or more separate pharmacological interventions during the current depressive episode. | Assessed via discussion during screening interview. The duration and dose of each antidepressant was recorded and screening team checked these were acceptable to expect a response. Lack of response but judged by screener with probing questions. |
| Participant agrees to be abstinent from illicit or extra-medical drug and alcohol use for at least 7 days prior to each psilocybin dosing session, and expects to be able to do so without any withdrawal issues | Self-report |
| Participant agrees to have their drug dosing sessions recorded to video and audio for treatment fidelity and clinical supervision within the study team | Self-report |
| Participant is fully vaccinated against COVID-19 or has an approved medical exemption to the vaccination | Self-report |

### Medical exclusion criteria

Table 2. Medical exclusion criteria

| **Criteria** | **Method for assessment and cut-offs** |
| --- | --- |
| Enrolled in another clinical trial involving any other investigational product or any other mental health treatment | Self-report |
| Any contraindicated disorder with known CNS involvement or other major CNS disease | Self-report |
| Hepatic dysfunction | Self-report and blood test.  Guidelines used to determine hepatic dysfunction:  GGT > 3 x ULN (upper limit of norm)  AST > 3 x ULN  ALT > 3 x ULN  Tot Bili > 3.0 mg/dL |
| Known conditions that put the participant at risk for hypercalcaemia. These include Cushing's syndrome, hypoglycaemia, syndrome of inappropriate antidiuretic hormone secretion, or carcinoid syndrome. | Self-report |
| Insulin-dependent diabetes; if taking oral hypoglycemic agents only excluded if they also have a history of hypoglycaemia. | Self-report |
| Contraindicated cardiovascular conditions. Includes uncontrolled hypertension, angina, a clinically significant ECG abnormality (e.g., atrial fibrillation), transient ischemic attack (TIA) in the last 6 months, stroke, or cerebrovascular disease, peripheral or pulmonary vascular disease (no active claudication) | Self-report, heart rate and blood pressure recordings, resting ECG.  Guideline for diagnosing uncontrolled hypertension: systolic >140 and diastolic >90 |
| Epilepsy or previous seizures | Self-report |
| Renal insufficiency | Self-report and creatinine clearance tested with blood sample.  Guideline for creatinine clearance: < 40 mL/min using the Cockraft and Gault equation |
| Pregnant, nursing, not using sufficient means of contraception *Only applies to women of childbearing potential  Adequate birth control methods are required for women of childbearing potential include:  Non-oral hormonal methods, including injected, intravaginal, implanted, transdermal (for example, intrauterine device (IUD), intrauterine hormone-releasing system (IUS))  Oral hormones plus a barrier contraception (condom, diaphragm, or spermicide)  Double barrier method (at least two of the following: condom, diaphragm, and spermicide)  Vasectomized partner(s) only  Abstinence from penile-vaginal intercourse | Self-report and pregnancy test prior to dosing sessions |
| Current hypothyroidism | Self-report |
| Weight < 40kg | Weight taken during medical exam |
| Taking opioid pain medications that cannot be ceased for 6 hours before and after the psilocybin dosing session  Long-acting opioid pain medications (e.g., oxycodone sustained release, morphine sustained release -- which are usually taken at 12-hour intervals) will be allowed if the last dose occurred at least 6 hours before psilocybin administration; such medication will not be taken again until at least 6 hours after psilocybin administration | Self-report and urine drug test (prior to dosing sessions) |
| Past 12-month use of macrodose hallucinogen or psychedelic  Includes psilocybin, MDMA, LSD, mescaline, DMT and other similar hallucinogenic compounds Does not include ketamine. | Self-report |
| Past 1-month microdose of any hallucinogen of psychedelic  Includes psilocybin, MDMA, LSD, mescaline, DMT and other similar hallucinogenic compounds Does not include ketamine. | Self-report |
| Illicit or extra-medical drug and alcohol use for at least 2 days prior to each psilocybin dosing session | Self-report and urine drug test (prior to dosing sessions) |
| Taking a contraindicated antidepressant medication (SSRIs, SNRIs, MAOIs), and 1) do not wish to be tapered off this medication, or 2) it is deemed by trial psychiatrists or the participant’s prescribing physician that tapering the participant off their current medication would be inappropriate, or 3) were unable to successfully taper off this medication completely. | Self-report use of antidepressant medications and willingness to taper. Absence of antidepressant medications or successful tapering is confirmed with participants’ treating healthcare professional.  Contraindicated medications determined by trial psychiatrist.  Urine drug tests (prior to dosing sessions) |
| Currently taking any contraindicated potent metabolic inducers or inhibitors and are unable or unwilling to cease these for the duration of the trial.  Inducers - Rifamycin (rifampin, rifabutin, rifapentine), anticonvulsants (carbamazepine, phenytoin, phenobarbital), nevirapine, efavirenz, paclitaxol, St John's Wort; Inhibitors - all HIV protease inhibitors, itraconazole, ketoconazole, erythromycin, clarithromycin, troleandomycin. | Self-report |
| Medically required to take any drugs with a low therapeutic index within 12 hours after psilocybin dosing sessions.  This includes, but is not limited to: ergot alkaloids, pimozide, midazolam, triazolam, lovastatin, simvastatin, fentanyl. | Self-report |
| Taking any other contraindicated medication | All current medications are self-reported, and the list carefully checked for any possible contraindications. If the participant cannot cease any possibly contraindicated medications for the duration of the trial, they will be excluded. |
| Significant, uncorrected visual impairment that would affect ability to view material presented on a computer screen. | Self-report |
| Participant is unable to swallow pills | Self-report |

### Psychiatric exclusion criteria

Table 3. Psychiatric exclusion criteria

| **Criteria** | **Method for assessment** |
| --- | --- |
| Severe symptoms of anxiety, depression warranting immediate hospitalisation or severe or sudden increased suicidality | Severe anxiety or depression warranting immediate hospitalisation is determined at the clinicians’ discretion, based on screening interview, including the QIDS and the DIAMOND.  Suicidality warranting exclusion from entering the trial, or exclusion from the dosing sessions is defined as follows:  A score of 3 on the QIDS suicidality question or an increase of 2 or more points on this question throughout the trial. |
| Current or past history of meeting DSM-5 criteria for Schizophrenia, Psychotic Disorder (unless substance-induced or due to a medical condition), or Bipolar I or II Disorder | Diagnosed during screening interview using the DIAMOND. |
| First degree relative with diagnosed Schizophrenia, Psychotic Disorder (unless substance-induced or due to a medical condition) or Bipolar I or II disorder | Self-report |
| Current or past history within the last 5 years of meeting DSM-5 criteria for alcohol or drug dependence (excluding caffeine and nicotine) | Self-report, AUDIT, DAST-10 and clinical interview using the DIAMOND. |
| Currently meets DSM-5 criteria for Dissociative Disorder, Anorexia Nervosa or Bulimia Nervosa | Self-report and clinical interview using the DIAMOND |
| Currently meets DSM-5 criteria for any psychiatric conditions (including personality disorders) judged to be incompatible with establishment of rapport or safe exposure to psilocybin. | LPFS-BF-2.0 to screen for personality disorders.  Clinical interview using a shortened version of the DIAMOND is also used to screen for social anxiety disorder, panic disorder, agoraphobia, generalised anxiety disorder, specific phobia,  separation anxiety, persistent depressive disorder, bipolar disorder I/II, major depressive disorder, cyclothymic disorder, premenstrual dysphoric disorder, acute stress disorder, post-traumatic stress disorder, adjustment disorder, schizophrenia and schizophreniform disorder, schizoaffective disorder, delusional disorder, anorexia nervosa, bulimia nervosa, binge eating disorder, avoidant/restrictive food intake disorder, substance use disorder, and suicidality  Clinician discretion is to be used in determining whether an identified psychiatric condition is incompatible with the establishment of rapport or safe exposure to psilocybin. |
| Any current personal of situational factors that, in the opinion of the investigators or study doctors, might interfere with participation (for example, lacking social support, lacking a stable living situation, current domestic violence, or other ongoing or recent trauma) | Custom questions included in video-call interview, however any material brought up during the course of the interview may be relevant in determining if participants meet this criteria. |
| Presence of factors leading to a ‘complex case’ of depression (for example: childhood trauma, multiple or complex psychiatric or medical comorbidities, or comorbidities where depression appears to be secondary) | Childhood trauma assessed with the ACE questionnaire. Information regarding comorbidities is assessed using self-report direct questions and the DIAMOND assessment tool, as well as through request for relevant information from primary healthcare provider. This criterion will be assessed at the clinician’s discretion using all information gathered during the interview. |
| Unable to give adequate informed consent | To be determined at the clinician’s discretion using information gathered throughout the informed consent process (e.g. ability to understand information provided) |

## Informed consent

All participants will receive a copy of the participant information and consent form (Appendix C) prior to completing the screening survey with consent implied by the completion of the following survey. At the start of the screening interview, an in-depth verbal explanation of the study will be given, and participants given the chance to ask questions and discuss any aspect of the study with trial coordinator and screening doctor. They will then sign the consent form, which will be co-signed by the trial coordinator.

# Trial Intervention

## Treatment overview

There are three distinct phases of the treatment: (1) Preparation: 60-90 minutes, psychotherapy sessions focused on building therapeutic alliance; taking the patient history; educating the participant about the effects of psilocybin, what to expect during the course of treatment, and the importance of set and setting; teaching skills and strategies that may be utilised during dosing sessions (anxiety management, non-avoidance training, etc.) and intention formation. (2) Dosing session: all-day, the participant administered 25 mg dose of psilocybin under the supervision of their therapist dyad, who provide non-directive support throughout the experience. (3) Integration: 60-90 minutes, psychotherapy sessions which focused on exploring the content of the dosing experience, integrating the experience into a wider context, sustaining any positive changes, along with a number of possible therapeutic approaches that may be tailored to the individual.

The trial intervention consisted of two dosing sessions, 4-6 weeks apart, with the second being optional, 3 preparatory psychotherapy (PPT) sessions preceded the first dosing session and 3 integrative psychotherapy (IPT) sessions followed each dosing session (see figure 1). PPT sessions occurred approximately 1 week apart in the 3-4 weeks prior to dose 1. IPT sessions 1 and 4 occurred the day after dose 1 and 2, respectively. IPT session 2 occurred within 3 weeks after dose 1, and IPT session 3 occurred within 1 week prior to dose 2. IPT sessions 5 and 6 occurred within the 4 weeks following dose 2.

The second dosing session was optional but encouraged. It was assumed that all participants would complete both doses, unless there was a clinical contraindication revealed during dose 1 (i.e., adverse medical of psychiatric reaction). If there was a suspected contraindication, this was discussed in IPT sessions with the participant, and amongst the trial team until a consensus was reached about the appropriate clinical decision. Participants could also choose not to complete the second dose for any reason, and they still received all other psychotherapy sessions.

Psychotherapy sessions were conducted face-to-face on site at Swinburne University. Exceptions were made where the participant or therapists were unwell and unable to reschedule the session to an appropriate time, in this case, sessions were conducted via zoom. Both members of the therapist dyad attended all sessions. Preparation and integration sessions were conducted in the same room as dosing to facilitate a sense of familiarity, safety and continuity. Exceptions were made for a maximum of 1 preparation and 1 integration session.

**
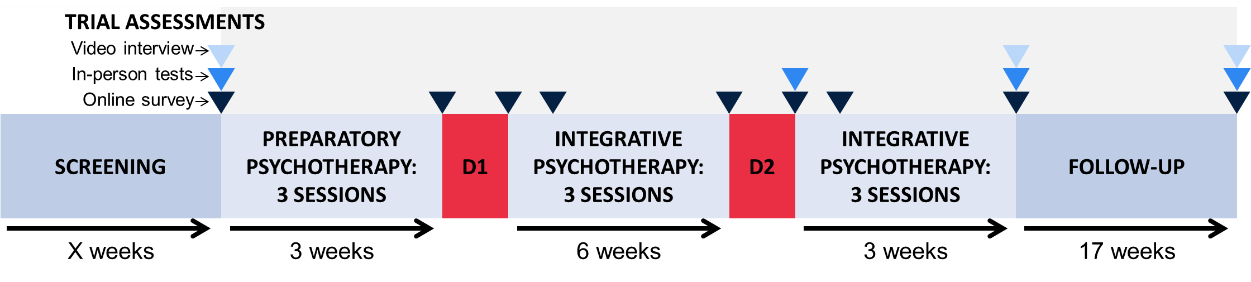
**

*Figure 1*. Outline of treatment phases and assessment timepoints

## Therapists and training

All participants were seen by the same two therapists from baseline through to the completion of the study. The therapist dyad consisted of either a male and female therapist or two therapists of the same gender as the participant. In every dyad, at least one therapist was a consultant psychiatrist or psychiatry registrar, ensuring appropriately qualified staff were present to administer trial medication, rescue medication if required, and manage any adverse events.

The trial therapists were qualified and experienced mental health professionals (including psychiatrists, psychologists, and psychotherapists) with specialty training in empirically based therapeutic modalities specific to clinical depression. Therapists were recruited by word of mouth. Therapists were required at minimum to hold a Masters level qualification in a relevant mental health area, have at least 1000 hours professional clinical time, and have some level of psychotherapy training and experience.

Trial therapists underwent a professional development training through online video training modules and 4-day in-person training intensive. These were delivered by local and international experts in clinical psychedelic practice and included talks, role play, group discussion and readings. Treatment procedures were not manualised, instead therapists were provided with a treatment guide to follow which is outlined in the sections below. Checklists were also created based on the procedures for each session outlined below. Therapists were asked to check off each item on the checklist before proceeding to the next phase.

There were two components to the therapists’ supervision arrangements:

1. Regularly scheduled group sessions with an expert supervisor with extensive experience in psychedelic therapies. All therapists were required to attend these sessions.
2. Dyad supervision sessions between PPT2 and 3 for each participant, with a principal investigator of the study with extensive clinical and supervision experience. These sessions focussed specifically on review of eligibility criteria and assessed participant safety to proceed to dosing, although they could also cover other topics. Additional sessions with this supervisor were available to therapists throughout the trial.

Additionally, therapists were encouraged to meet with their dyad partner to review each case, as necessary.

## Preparatory psychotherapy sessions

The broad goals for therapists across the three preparation sessions were:

- Build trust and rapport with the participant. Create a space in which the participant feels safe and supported, and able to freely share their history and current relationship with depression.
- Explore potential exclusionary factors. See ‘Monitoring and reviewing eligibility’ section for more details.
- Discuss all physical and psychological safety measures and dosing day instructions; confirm the participant understands the nature of the treatment and that they are committed to following all safety instructions.
- Address key opportunities and challenges of the psychedelic experience and the central role of trust, openness and curiosity.
- Outline common psychedelic experiences, including sensory, emotional, autobiographical and transpersonal/metaphorical/archetypal.
- Indicate that it can be difficult to distinguish between the literal and metaphorical within the psychedelic experience and highlight the benefit of a functional over a literal approach to interpreting experiences and insights.
- Document any adverse events and case notes using data collection tool (RedCap).

Additionally, each preparatory session had specific aims:

### Session 1: The participant and the approach

- Introductions - Brief therapist introductions, create a safe, warm, and supportive space. Encourage questions, openness, and honesty.
- Participant information - Take patient history, explore the nature of their current mental health and their journey to this point.
- Description and reasons for set-setting support
- Treatment overview – Brief description of broad treatment approach
- Holistic preparation – Discuss benefits of holistic approach, particularly prior to the dosing session, including sleep, diet, exercise, as well as broadly cultivating daily openness, curiosity, or mindfulness to one’s inner life.
- Additional support – Inform participant of additional supports available (see ‘Additional support during the treatment phase’ section for more details)
- Five participant agreements – Outline each one, the reasoning, and ask them to agree to follow all.
  1. Follow guidance: I will follow safety instructions from my therapists or other study staff.
  2. Await discharge: I will remain within the treatment facility during dosing day until discharged by my study therapists.
  3. No harm: I will cause no harm to myself or anyone else throughout the study.
  4. No damage: I will cause no damage to any property at the treatment facility throughout the study.
  5. Seek help: I will immediately call my study therapist, the Trial Coordinator, or emergency (dial 000) if I experience any suicidal thoughts or feelings throughout the study.
- Support person – Outline the reasons for this and ask them to nominate a support person (for more details see ‘Support person’ section).
- Safety and support priority – Remind the participant that their safety is of primary importance, above any data collection or other research-related activities, and that the team is there to support them.
- Provide the participant with a copy of the Participant information sheet (Appendix D).
- Give the participant the gift diary and pen to use throughout the trial if they wish.

### Session 2: The psychedelic experience and tool for navigating it

- Psychedelic experiences: Educate the participant on common experiences with psilocybin. Briefly outline the possible changes to physical sensation, perception, cognition, affect and basic state of consciousness. Note that this can be highly variable across people and that the experience can feel very unusual, including changes to fundamental experiences like their sense of time, space, and self. Note that the experience can be uplifting but also very challenging.
- Experiential avoidance: Introduce the importance of openness and trust, regardless of how the psychedelic experience unfolds (“trust, let go, be open”)
- Anxiety management strategies: Teach anxiety management strategies, such as mindfulness-based techniques for grounding. Inform the participant that, while it may at times feel like the experience is too much, and some people worry they will not return, know that this is safe and they will return.
- Intentions: Explain the idea of intention setting. Explore one or a few possible intentions. Lightly suggest two intentions: one relating to the theme of trust in the psychedelic process; the other relating to their experience of depression or related struggles. Good intentions are a) internalised, b) specific, c) positive, and d) open. Once clarified, ask the participant to write down their intention(s) and state the intentions out loud if willing.
- Music: Let the participant know there is a music playlist that will be used for the duration of the dosing session and two back-up options. Outline these options and explain the role of music in the process. Highlight that it is important not to attempt to control one’s experience through the music selection or otherwise. Let participants trial the headphones and listen to a section of the playlist.

### Session 3: Outline dosing day practicalities

- Role of the therapists: Remind the participant of the role of the therapists; to monitor and provide support to the participant, to ensure treatment is safe and opportunities for benefit are maximised.
- Dosing day plan: Provide the participant with a hardcopy of the ‘Dose Day Participant Instruction Sheet’ (Appendix E) and walk them through each point, including what to do in the days leading up to the dosing session (sleep, stress minimisation, use of drugs and alcohol), what to wear and bring to the dosing session, the procedures on the day of dosing (COVID-19 test, drug and pregnancy tests, blood pressure monitoring, rescue medication options, support person, etc.), and what to do in the days following the dosing session (driving, time off work, etc.).
- Therapeutic touch: Discuss the purpose and use of therapeutic touch with the participant. Practise therapeutic touch and ‘stop’ phrases/gestures. Document consent using data collection tool (REDCap). See ‘Therapeutic touch’ section for more details.

## Dosing sessions

### Psilocybin

Psilocybin was provided by Usona Institute (Madison, Wisconsin, USA.) in the form of opaque, size 0 gelatine capsules each containing 25 mg psilocybin with lactose excipient.

Psilocybin was obtained and stored under a Licence to Import Substances Subject to Schedule 4 of the Customs (Prohibited Imports) Regulations 1956 and Permit to Import Substances Subject to Regulation 5 of the Customs (Prohibited Imports) Regulations 1956 from the Australian Government, Office of Drug Control, and a Permit to Purchase or Otherwise Obtain Poisons or Controlled Substances for Industrial, Educational or Research Purposes from the Victorian State Government. Psilocybin was **administered under Permits to Administer, Supply or Prescribe Schedule 9 Poisons by a Registered Medical Practitioner for a Clinical Trial, and after July 1st 2023, under the Schedule 8 Notification Scheme with the Victorian State Government.**

### Dosing session procedures

- Therapist will set up the room prior to participant arriving, ensuring furniture is appropriately positioned, all equipment is present (study drug, rescue medications, blankets and pillows, headphones and sound system, eyeshades, water and snacks, emesis container, vital signs monitor), and video camera is on and recording.
- The participant will arrive at approximately 9.00am and be greeted by their therapists in the building foyer. A picture of their COVID-19 rapid antigen test results will be checked (participants are instructed to take a test the day prior and take a photo of the result).
- Therapists will check in with the participant regarding how they are feeling about the session and if they are ready to proceed. They will review the elements of the preparatory phase (e.g., grounding practices, intentions, safety procedures)
- Any adverse events from the period prior to dosing will be recorded.
- Participant will sign a drug use declaration, confirming they have not used any contraindicated substances or any medications outside of their approved medications.
- Participant will provide a urine sample. The medically trained therapist will conduct a drug test and a pregnancy test.
- The medically trained therapist will take two blood pressure and heart rate baseline readings.
- The therapists will review the participant’s eligibility and complete an eligibility reassessment form in the data collection system (REDCap), only proceeding if the participant meets all criteria.
- The participant completes a brief survey.
- The medically trained therapist will provide the participant with one 25 mg capsule of psilocybin at approximately 10.00am with a glass of water.
- The participant will be encouraged to lie comfortably on the couch, wearing the eyeshades and listing to the playlist through the headphones. They will be directed to attend to their internal experience although they may speak to the therapists whenever they wish. Throughout the session, participant-led, non-directive support will be provided as needed.
- After approximately 1 hour has passed, if the participant has not spoken, the therapists may check in with them about the nature of their experience. For the rest of the session, as appropriate, an internal focus will be encouraged.
- If hardly any effects are experienced after 90 minutes, the participant may be offered a small snack.
- During the later part of the session, the participant will be encouraged to eat some food.
- At approximately 3.00-4.00pm, when the psychoactive effects of the psilocybin have mostly subsided, the therapists will facilitate a final conversation to close the session. This will not include integrative discussions. The participant will be reminded to remain in a supportive, calm and quiet environment, to reflect on their experiences and to do some journaling about their experience. They will also be reminded of the phone number they may use to contact the therapists if needed, over the next 24 hours.
- The session will be concluded when the participant is ready to leave and the therapists determine they are psychologically and medically stable.
- The participant’s support person will enter the room to collect them.
- Any adverse events occurring during the dosing session and brief case notes will be recorded.

### Vital signs monitoring

An automatic blood pressure and heart rate monitor will be used to monitor participant vital signs throughout the dosing sessions. Two manual recordings will be taken prior to administration of the study drug. After the study drug is administered, recordings will be automatically taken every 30 minutes for the first two hours. Further recordings may be taken after 2 hours if deemed clinically relevant. If a recording falls outside of the following thresholds, additional recordings will be taken every 5 minutes, until the recordings fall within the thresholds.

- Systolic:
  - High: 160
  - Low: 90
- Diastolic
  - High: 110
  - Low: 60
- Heart rate
  - High: 110
  - Low: 40

### Rescue medications

A small amount of lorazepam will be kept in the dosing room during all dosing sessions for use as a ‘rescue medication’, in the event that a participant has an adverse psychological reaction to the study drug that does not respond to other management strategies (i.e., verbal reassurance and implementation of techniques taught to participants in preparatory psychotherapy sessions). The participant will be informed about the possible use of rescue medications in the preparatory psychotherapy sessions. Only the medically trained therapist will administer the rescue medication and will be responsible for the decision to do so. A single dose may be between 0.5-2 mg, with a total maximum of 2 mg, and is to be determined at the clinician’s discretion.

### Video recording

All dosing sessions will be recorded to video. The use of video recording will be discussed with participants during the informed consent procedure and during preparatory psychotherapy sessions. The camera used will be small and placed discreetly, as to not interfere with the session.

These recordings will be used within the study team to assess treatment fidelity. They may be called on during therapist group supervision sessions to assess and analyse specific moments of the dosing session. The recordings will also viewed by either the trial coordinator or the principal investigator in the event of a participant or therapist complaint or safety concern.

### Setting

The therapy room is a moderate sized carpeted room in a university building with a large non-opening window through which trees can be seen with no ability for the public to look in. The building mostly contains staff offices and is generally quiet with little foot traffic. The room is in a small sectioned-off area with other offices and clinical rooms which requires staff card swipe access, further limiting the foot traffic in the immediate area. The room has a large, frosted glass door which will be blocked from view from the therapy area with a bamboo room divider, providing further privacy. The bathroom is a short walk away on the same floor and mirrors will be covered in this bathroom before dosing sessions.

The room will be furnished and decorated to create a living-room-like environment. This includes a large L shaped couch for participants (with enough space to sit or lay down comfortably), two soft armchairs for therapists, a variety of small coffee tables, vases with flowers, candles, pot plants, and large nature-related and abstract artwork. Any clinical equipment (vital signs monitor, computer, drug storage trolley, camera) will be kept to a minimum and concealed where possible. A variety of blankets and pillows will be provided for the participant to make themselves comfortable during dosing sessions. Meditation floor cushions will be available for therapists to sit closer to the participant during dosing sessions. Provided decorations will remain non-religious and participants will be encouraged to bring in any objects or photos that are personally meaningful for them. The window has partial and full block out blinds. A number of lamps will be used for additional lighting with adjustable light temperature and brightness to create a relaxing environment. The room has centrally controlled air conditioning and generally remains at a stable pleasant temperature. A fan will be used for added ventilation when necessary and a microwaveable heat pack will be available for participant use.

### Music

The participant will be given wireless noise-cancelling headphones to listen to a preprepared music playlist. Music is also played from a speaker in the room at the same time, so therapists can hear what the participant is listening to.

There were four playlists that could be used during dosing session in this trial. Two primary playlists were created by Sean O’Carroll, one of the trial therapists. These primary playlists contain evocative music that has been chosen to enhance and work with the dosing experience. These playlists are the preferred music choice and participants were encouraged to use these playlists. However, there were two additional playlists, one contained only mellow/ambient music and another only nature sounds. These could be used if necessary, for example, if it is felt that the music was obstructing/impeding the experience or if the music was negatively overwhelming.

The two main playlists consisted of a range of music including classical, new age, ambient, folk, meditative, chanting, overtone singing, and Icaros. Overall, the music contained few lyrics and very few lyrics in English. Songs were chosen to be unfamiliar to participants; avoiding popular western music that participants would have likely encountered and may carry memories and associations.

The playlists were arranged into four phases to follow the typical experience:

1. Ascent: A phase in which the participant goes from experiencing no effect to the gradual emergence of physical, perceptual, and affective changes. Music during this phase is spacious and gently stirring, often involving nature sounds.
2. Peak: A phase in which the participant is experiencing the full effect of the psilocybin. Music during this phase is focused, heartful, supportive and encouraging.
3. Descent: A phase in which the full effects of the psilocybin begin to ease, and do not return to full intensity.
4. Return: A phase in which the participant is obviously beginning to return to ordinary waking consciousness, and perhaps interacting with therapists more lucidly.

Both playlists had over 7.5 hours of music and were created to cover long experiences; if a participant had a faster experience or were proceeding through the phases in a different manner, adjustments were made by therapists during the session.

[Swinburne Psychedelic Trial Playlist #1](https://open.spotify.com/playlist/3aPsJ3cqSHuJRJRXlJ2kDn?si=750c65a323a24c5e) https://open.spotify.com/playlist/3aPsJ3cqSHuJRJRXlJ2kDn?si=750c65a323a24c5e&nd=1

[Swinburne Psychedelic Trial Playlist #2](https://open.spotify.com/playlist/74j5c9Rnup2UkZmJauzXRn?si=bee46c3737d64526) 
https://open.spotify.com/playlist/74j5c9Rnup2UkZmJauzXRn?si=bee46c3737d64526&nd=1

[Stress Relief (calm, ambient music)](https://open.spotify.com/playlist/37i9dQZF1DWXe9gFZP0gtP?si=808f384c14e44ed3) 
https://open.spotify.com/playlist/37i9dQZF1DWXe9gFZP0gtP?si=808f384c14e44ed3&nd=1

[Nature Sounds](https://open.spotify.com/playlist/37i9dQZF1DX4PP3DA4J0N8?si=c05afea566f84d6b) 
https://open.spotify.com/playlist/37i9dQZF1DX4PP3DA4J0N8?si=c05afea566f84d6b&nd=1

### Therapeutic touch

Therapeutic touch is the use of gentle and reassuring touch, for example, through holding the participant’s hand or placing one’s hand on the participant’s shoulder. Touch in this context is used only to reassure, comfort and ground the participant; it is not a central focus of the intervention as in touch-based therapeutic techniques where the aim of touch is to achieve deeper therapeutic goals such as releasing trauma. Thus, minimal use of therapeutic touch was permitted within this trial, with therapeutic touch being optional for both the participants and therapists. Therapeutic touch was limited to dosing sessions and practised during preparation sessions. Therapists were advised to consider both their own intentions and the possible perception of touch by the participant. Touch could never be sexual or sensual.

During preparatory psychotherapy sessions, therapists went through an in-depth discussion of therapeutic touch with the participant, they explained the purpose/utility of touch and its limitations, enquired how the participant feels about therapeutic touch and encouraged them to ask any questions they may have. Therapists were advised to be cautious of implicit coercion during consent. The therapists asked the participant (and documented in the data collection tool) if they consented to therapeutic touch, any specificities or limitations to touch (i.e., only hand holding), which therapists they consented to receive therapeutic touch from, whether touch was therapist- and/or participant-initiated, and which verbal and non-verbal methods would be used to decline touch. These forms of therapeutic touch and ‘stop’ words and gestures were practised during the preparatory phase. Participants were reminded that they can withdraw their consent to therapeutic touch at any time. Participants were provided with a copy of the ‘PsiloTRD Participant Information Sheet’ (Appendix D) which included an outline of the therapeutic touch protocol.

Any use of therapeutic touch during dosing sessions was documented in the data collection tool. Prior to the second dosing session, therapists revisited therapeutic touch with the participant. and enquired if they would like anything to be done differently or if they wished to amend their consent.

All therapists were asked to sign an agreement document which outlined the use and strict limitations on therapeutic touch in this trial prior to commencing work (Appendix F).

### Support person

Participants were asked during preparatory psychotherapy to designate a close friend or family member to be their ‘support person’. This person escorted them home after dosing sessions and stayed with them for the following 24 hours. This support person could also escort them to and from data collection and psychotherapy sessions if desired. Therapists had a phone conversation with the designated support person prior to dosing sessions to ensure they were suitable and informed them on how to best support the participant after the dosing session.

The support person was advised they should provide a sensitive and caring environment free from stress, media, need to speak, or unwanted visitors, and encourage journaling or quiet reflection. They were informed that one of the therapists would be on call for 24 hours after dosing, and the Trial Coordinator can be contacted outside of this time, if needed. This information was summarised in a Support Person Information Sheet (Appendix G).

The support person was also be asked if they wish to participate in the study by responding to three 10-minute surveys throughout the trial. These surveys contained standardised questionnaires that asked them about the functioning of their friend/family member. They were informed that their participation was voluntary and separate to their role as the support person, that is, they can choose not to participate and still fulfill their role as support person. If they wished to participate, they were emailed links to the surveys, the first of which began with a Support Person Participant Information and Consent Form (Appendix H). This was electronically signed prior to proceeding to the survey itself.

## Integrative psychotherapy sessions

Therapist guidelines for the conduct of the integrative psychotherapy sessions:

- Start and finish the session with a brief process that supports therapeutic trust, reconnection with psychedelic experience, and psychotherapeutic work.
- Explore the psychedelic experience: describing details of the journey, written reflections since the experience, exploring intentions prior to dosing, and any new insights, shifts in values and priorities with a focus on letting go of unhelpful old stories where relevant. Highlight the need to hold interpretations lightly and remind the participants that it can be difficult to distinguish between the metaphoric and literal during a psychedelic experience.
- Make suggestions for ongoing integration and supportive practice: can include mindfulness practises; time in nature; journaling; reflecting; creative arts, dance, music; participating in community events; volunteering time to those in need.
- Discuss sharing of the experience with family, friends, coworkers: Discuss trusting and supportive environments for sharing and that experiences of non-ordinary states of consciousness may not be understood, accepted, or welcomed by others, which can lead to feelings of rejection, isolation, and ostracism in the person attempting to share.
- Assess any protracted effects of psychedelics, and provide support where needed
  - Persisting hallucinations
  - Intensification of depressive symptoms
  - Suicidal ideation
  - Heightened anxiety or paranoia
  - Depersonalization
  - Any other issues of clinical concern
- Discuss how experience of depression and quality of life have changed as well as any persistent or emergent challenges with mental health.
- Develop post-study plan for continued integration if participant is willing: structure around participant’s intentions and context explored throughout the integration sessions. Examples include reconnecting with family or friends, reigniting old hobbies/work or exploring new ones, shifting unhelpful behaviour or thinking patterns, becoming more involved in family, friendships, or community. Post-study plan must also include referral(s) to relevant mental healthcare professionals for continued integration and support.
- As the final session approaches, explore feelings around the end of the trial, remembering that it can be challenging to draw the therapeutic relationship to a close for both parties. Consider an intentional closing process in the final sessions.

## Monitoring and reviewing eligibility

Throughout preparatory psychotherapy sessions, therapists were required to tactfully explore potential exclusionary factors. This included assessing psychological preparedness and any substantial difficulty with trust and rapport (either from therapist or from participant) that emerged during preparation. Therapists were required to have good knowledge and recall of all relevant exclusionary criteria. If any exclusionary factors seemed probable, they discussed in therapist supervisions sessions and with the trial coordinator prior to dosing.

On the morning of each dosing session, a final check of eligibility for dosing was conducted, including a drug and pregnancy urine test, blood pressure and heart rate assessments, and a discussion with the participant. Therapists were required to complete and sign a final eligibility checklist prior to dosing.

Table X: Key eligibility criteria reviewed prior to dosing

| **Criteria** | **Methods for assessment and cut-offs** |
| --- | --- |
| No contraindicated medication or drug use, including:   - Any illicit or psychoactive drug, or alcohol in the past 2 days - Any antidepressant medication since the start of the trial - Opioid pain medications within 6 hours of dosing - Psychedelics of MDMA at any point during the trial | A urine drug test will be performed on the morning of each dose to test for recent use of the following substances: THC, Opiates, Amphetamines, Methamphetamine, Cocaine, Benzodiazepine, Methadone, Oxycodone, Buprenorphine, Barbiturates, MDMA, Tramadol, Phencyclidine, Tricyclic antidepressants, Fentanyl, Alcohol.  All participants are also asked to sign a form which outlines the drugs they are not permitted to use within certain time frames prior to dosing and asking them to declare the absence of such drug use.  In the event of a positive drug test result, priority will be given to self-reported use, given the window of detection for some substances is greater than the window of prohibited use. |
| Absence of current pregnancy | Urine pregnancy test conducted on the morning of each dosing session for WOCBP |
| Absence of any contraindicated physical/medical or psychiatric conditions | This criterion is fulfilled by the absence of any relevant symptoms or changes reported spontaneously or during routine questioning, both documented in the adverse event forms. Staff must check these prior to dosing sessions. If the participant has possibly developed the condition, consultation with their treating physician may be necessary before the session can proceed. |
| Heart rate and blood pressure within healthy range prior to dosing | Heart rate and blood pressure measurements taken on the morning of each dosing session.  Guidelines:  HR <100 BPM  BP <140/90 mmHg |
| Adequate therapeutic alliance has been developed and maintained | Information gathered throughout the psychotherapy sessions.  Determined at the clinician’s discretion. |
| Participant appears adequately psychologically stable and prepared | Information gathered on the day of the dosing session.  Determined at the clinician’s discretion. |

## Participant information and instructions

In addition to the information contained in the PICF, participants were also provided with a Participant Information sheet (Appendix D) during preparatory psychotherapy. This information sheet provided treatment details, including an outline of the three phases, details of the use of therapeutic touch in this treatment, the 24-hour phone line support following each dose, availability of additional therapist support outside of schedule sessions, and the role of the support person. It also contained instructions regarding the use of external treatments (psychotherapy and medications), additional details regarding trial assessments, COVID-19 regulations, their general responsibilities as participants in this trial, contact information for key staff and the HREC, and additional/emergency support lines.

Participants were also emailed a Dosing Day Information Sheet (Appendix E) at both 1 week and 1 day prior to each dosing session. This sheet contained instructions for the days leading up to and the morning of their dosing session, as well as a reminder of what will happen on the dosing day.

This information was a repetition of information provided in the PICF and preparatory psychotherapy, but in a succinct written form.

## Medications and drug use throughout the study

Participants were asked to provide a full list of all medications and supplements they take at screening which was reviewed by our study doctor. Participants were requested to cease any contraindicated medications prior to enrolment or for a certain number of days prior to dosing, depending on the medication, and if unable to cease taking a contraindicated medication they were deemed ineligible for participation. Participants were instructed to avoid changing medications during the trial and if necessary, inform the trial coordinator of any medication changes. Participants were requested to remain off antidepressant medication until the primary endpoint (3-weeks post-dose 2). Participants were requested to abstain from illicit drugs and alcohol for 7 days prior to and 2 days following each dosing session.

## Additional support during the treatment phase

Throughout the intervention, and up until discharge from the study after the long-term follow-up, participants could request, or therapists may suggest, further support including additional psychotherapy sessions or phone/video-call support. All direct therapist-participant contact outside of scheduled sessions was pre-approved by the trial coordinator and documented.

Participants were encouraged to continue to see any existing mental healthcare professionals throughout the trial, however they were requested not to start any new structured therapies or begin seeing any new mental healthcare professionals for the duration of the active treatment phase of the trial.

## Post-treatment phase support

For all participants, a tailored discharge plan was devised prior to completion of the treatment and typically involved referral to a mental healthcare provider with knowledge of psychedelic therapy or other relevant skills.

## Participant reimbursement

Upon completion of the study, participant were provided with $200 for travel costs related to study participation. If requested, further compensation for taxi fares was provided.

# Trial Assessments

## Schedule

Data was collected at multiple timepoints throughout the trial (see Table 1). Baseline assessments were conducted 3-5 weeks prior to dose 1, and prior to treatment commencing. One-day pre-dose 1/2 assessments were conducted 1 day prior to dosing, while 1-day post-dose 1/2 assessments were conducted 1-day post-dose, although this could have been up to 2 days post-dose, if necessary. One-week post-dose 1 or 2 assessments were conducted 1-week post-dose, however these were sometimes up to 10 days post-dose, if necessary. Three-weeks post-dose 2 assessments were conducted 3-5 weeks post-dose 2, and only after all IPT is completed. Long-term follow-up assessments were conducted within 26-27 weeks post-dose 1.

Table 1. Data collection schedule

| Data Collection Instrument | Baseline | P-PT 1 | P-PT 2 | P-PT 3 | 1-D Pre-D1 | Dose 1 | 1-D Post-D1 | I-PT 1 | 1-W Post-D1 | I-PT 2 | 3-W Post-D1 | I-PT 3 | 1-D Pre-D2 | Dose 2 | 1-D Post-D2 | I-PT 4 | 1-W Post-D2 | I-PT 5 | I-PT 6 | 3-W Post-D2 | 20-W Post-D2 |
| --- | --- | --- | --- | --- | --- | --- | --- | --- | --- | --- | --- | --- | --- | --- | --- | --- | --- | --- | --- | --- | --- |
|  |  |  |  |  |  |  |  |  |  |  |  |  |  |  |  |  |  |  |  |  |  |
| Demographics | x |  |  |  |  |  |  |  |  |  |  |  |  |  |  |  |  |  |  |  |  |
| PATHEV | x |  |  |  | x |  |  |  |  |  |  |  |  |  |  |  |  |  |  |  |  |
| TIPI | x |  |  |  |  |  |  |  |  |  |  |  |  |  |  |  |  |  |  | x | x |
| Blood test | x |  |  |  |  |  |  | x |  |  |  |  |  |  |  |  |  |  |  | x |  |
| Qualitative Interview | x |  |  |  |  |  |  |  |  |  |  |  |  |  |  |  |  |  |  | x | x |
| QIDS-SR | x |  |  |  | x |  |  |  | x |  | x |  | x |  |  |  | x |  |  | x | x |
| WHO-Qol-Bref | x |  |  |  |  |  |  |  |  |  | x |  |  |  |  |  |  |  |  | x | x |
| GAD-7 | x |  |  |  |  |  |  |  |  |  | x |  |  |  |  |  |  |  |  | x | x |
| BEAQ | x |  |  |  |  |  |  |  |  |  | x |  |  |  |  |  |  |  |  | x | x |
| RSES | x |  |  |  |  |  |  |  |  |  |  |  |  |  |  |  |  |  |  | x | x |
| WCS | x |  |  |  |  |  |  |  |  |  |  |  |  |  |  |  |  |  |  | x | x |
| Mind(SET) |  |  |  |  |  | x |  |  |  |  |  |  |  | x |  |  |  |  |  |  |  |
| 11D-ASC |  |  |  |  |  |  | x |  |  |  |  |  |  |  | x |  |  |  |  |  |  |
| MEQ |  |  |  |  |  |  | x |  |  |  |  |  |  |  | x |  |  |  |  |  |  |
| EBI |  |  |  |  |  |  | x |  |  |  |  |  |  |  | x |  |  |  |  |  |  |
| PIQ |  |  |  |  |  |  | x |  |  |  |  |  |  |  | x |  |  |  |  |  |  |
| Custom questions | x |  |  |  |  |  | x |  |  |  |  |  |  |  | x |  |  |  |  | x | x |
| JHU questions |  |  |  |  |  |  |  |  |  |  |  |  |  |  |  |  |  |  |  | x |  |
| PEQ |  |  |  |  |  |  |  |  |  |  |  |  |  |  |  |  |  |  |  |  | x |
| Cognitive Test Battery | x |  |  |  |  |  |  |  |  |  |  |  |  |  | x |  |  |  |  | x | x |
| Social processing tasks | x |  |  |  |  |  |  |  |  |  |  |  |  |  | x |  |  |  |  | x | x |
| CFS | x |  |  |  |  |  |  |  |  |  |  |  |  |  | x |  |  |  |  | x | x |
| VDQ | x |  |  |  |  |  |  |  |  |  |  |  |  |  | x |  |  |  |  | x | x |
| TAS20 | x |  |  |  |  |  |  |  |  |  |  |  |  |  | x |  |  |  |  | x | x |
| IIP-32 SR | x |  |  |  |  |  |  |  |  |  |  |  |  |  |  |  |  |  |  | x | x |
| IIP-32 PR |  | x |  |  |  |  |  |  |  |  |  |  |  |  |  |  |  |  |  | x | x |
| External support questionnaire |  |  |  |  |  |  |  |  |  |  |  |  |  |  |  |  |  |  |  |  | x |
| Participant feedback |  |  |  |  | x |  | x |  | x |  | x |  | x |  | x |  | x |  |  | x | x |
| Recent drug use form |  |  |  |  |  | x |  |  |  |  |  |  |  | x |  |  |  |  |  |  |  |
| HR/BP |  |  |  |  |  | x |  |  |  |  |  |  |  | x |  |  |  |  |  |  |  |
| Physical contact consent form |  |  |  | x |  |  |  |  |  |  |  | x |  |  |  |  |  |  |  |  |  |
| HPPD-Q |  |  |  |  |  |  |  |  |  |  |  |  |  |  |  |  |  |  |  |  | x |
| Physical contact questionnaire |  |  |  |  |  |  |  |  |  |  |  |  |  |  |  |  |  |  |  | x |  |
| Adverse Events Form | x | x | x | x | x | x |  | x |  | x |  | x | x | x | x | x |  | x | x | x | x |

P-PT = Preparatory psychotherapy. I-PT = Integrative psychotherapy. 1-D = 1-day. 1-W = 1-week. 3-W = 3-weeks. 20-W = 20-weeks.
D1/D2 = dose 1/dose 2.

## Measures

### Primary Measures

- 16-item Quick Inventory of Depressive Symptomatology – Self-rated (QIDS; Rush et al., 2003). The QIDS is a self-rated measure of depression that offered the advantages of brevity, increasingly widespread use, and validity at 1-week intervals. This measure assessed nine domains: sad mood, concentration, self-outlook, suicidal ideation, involvement, energy/fatiguability, sleep disturbance, appetite/weight increase/decrease, and psychomotor agitation/retardation. It consisted of 16 items rated from 0-3. This scale was administered at baseline, and throughout the study.

### Secondary Measures

- Demographics: a custom series of questions was used to collect basic demographic information including sex, employment, education, marital status and ethnicity/cultural background. Demographics questions were administered at baseline.
- Patients’ Therapy Expectations and Evaluation (PATHEV; Schulte, 2008) was used to measure participants’ expectations at baseline and 1 day prior to the first dosing session. The questionnaire consisted of 11 statements each rated on a Likert scale from 1 (absolutely wrong) to 5 (absolutely right). Three factor analytically derived subscales were assessed: Hope of Improvement, Fear of Change, and Suitability. The PATHEV was administered at baseline and again at 1-day pre-dose 1 to assess for any changes in expectations after completing the preparatory psychotherapy component of the treatment.
- The Ten Item Personality Inventory (TIPI; Gosling, Rentfrow, & Swann Jr, 2003). This very brief self-report questionnaire assessed the big five personality dimensions: extraversion, agreeableness, conscientiousness, neuroticism, and openness to experience. This scale consisted of 10 items, each rated on a 1-7 Likert-scale (disagree strongly to agree strongly) and was administered at baseline and at 3- and 20- weeks post-dose 2.
- Plasma BDNF levels were collected for use as a marker of neuroplasticity. A 10 mL blood sample was taken by a qualified staff member at baseline, 1-day post-dose 1 and 3-weeks post-dose 2. Samples were collected in Ethylenediaminetetraacetic acid (EDTA) tubes, processed and aliquoted into 3 microtubes for storage in an -80 ˚C freezer. All samples were analysed after study completion.
- Semi-structured qualitative interviews were conducted three times for each participant via video-call at baseline, 3-weeks and 20-weeks post-dose 2. The baseline semi-structured interview focused on exploring the participant’s subjective experience of their depression and how this has impacted their life, as well as their expectations and hopes regarding the study treatments. The post-intervention interview focused on the participant’s subjective experience of the study treatment and any effects it had on their depression and other aspects of their life. The long-term follow-up interview focused on the lasting effects the study treatment had on their depression and other aspects since the previous interview. The semi-structured interviews were designed to enable the participant to explore these ideas in their own words and are based on those used by Belser and colleagues (2017). Interviews were conducted by study staff not involved in other aspects of the trial, with training in qualitative interviewing.
- World Health Organization Quality of Life Questionnaire - Brief Version (WHO-QoL-Bref; WHO, 1998). This 26-item, self-report measure was developed by the WHO to assess quality of life in the following areas: physical, psychological, level of independence, social relationships, environment, and spirituality/religion/personal beliefs. Responses were rated on a 5-point Likert scale ranging from 1 = (not at all, very poor, very dissatisfied, never) through to 5 = (very good, very satisfied, an extreme amount, completely, always). Quality of life was assessed at baseline, 3-week post-dose 1 and 2, and 20-weeks post-dose 2.
- Generalized Anxiety Disorder 7-item scale (GAD-7; Spitzer, Kroenke, Williams, & Löwe, 2006). The GAD-7 is a brief self-report measure of generalised anxiety, it consisted of 7 items rated from 0 (‘not at all sure’) to 3 (‘nearly every day’). It has good psychometric properties and is a widely used research instrument in assessing adult anxiety. The GAD-7 was administered at baseline, 3-weeks post-dose 1 and 2, and 20-weeks post-dose 2.
- Brief Experiential Avoidance Questionnaire (BEAQ; Gámez et al., 2014). This measure assessed unwillingness to remain in contact with distressing thoughts, emotions, and physical sensations, even when such avoidance leads to adverse outcomes in the long-term. The BEAQ consisted of 15 items rated on a Likert scale ranging from 1 to 6 (strongly disagree to strongly agree) and covers six dimensions: behavioural avoidance, distress aversion, procrastination, distraction/suppression, repression/denial, distress endurance. The BEAQ was administered at baseline, 3-weeks post-dose 1 and 2, and 20-weeks post-dose 2.
- Two subscales from the Revised Self-Efficacy Scale (RSES; McDermott, 1995) were used to measure everyday living and social self-efficacy. In accordance with the use of these subscales by Cardenas et al. (2013), the 19-item Everyday Living and 16-item Social subscale items were rated on a 5-point Likert scale (1 = not at all; 5 = extremely) to examine how confident people were in performing everyday living or social behaviours. The RSES subscales was administered at baseline, 3- and 20-weeks post-dose 2.
- Watts Connectedness Scale (WCS; Watts et al., 2022). This measure consisted of 19 items that probed feelings of connectedness to self, other, and the world over the past two weeks. Items were rated on a 0–100 visual analogue scale (VAS), where 0 corresponded to ‘not at all’ and 100 to ‘entirely’. The WCS was administered at baseline, 3- and 20-weeks post-dose 2.
- Mind(SET) Questionnaire (Haijen et al., 2018). This self-report questionnaire assessed the respondent’s mindset prior to drug intake. It consisted of 11 statements, each rated on a VAS of 0 – ‘strongly disagree’ to 100 – ‘strongly agree’. This measure was administered just prior to dosing. The Mind(SET) was administered on the morning of each dosing session prior to psilocybin administration.
- 11 Dimension Altered States of Consciousness scale (11D-ASC; Studerus, Kometer, Hasler, & Vollenweider, 2011). This measure evaluated 11 dimensions of altered states of consciousness: experience of unity, spiritual experience, blissful state, insightfulness, disembodiment, impaired control and cognition, anxiety, complex imagery, elementary imagery, audio-visual synaesthesia, and changed meaning of percepts. It consisted of 42 items, with responses made by placing marks on horizontal VAS with ‘no, not more than usual’ on the left (0) and as ‘yes, very much more than usual’ on the right (100). The 11D-ASC was administered 1-day post-dose 1 and 2.
- 30-item Mystical Experiences Questionnaire (MEQ; MacLean, Leoutsakos, Johnson, & Griffiths, 2012). It assessed 4 empirically derived factors: mystical experiences (including ‘internal unity’, ‘external unity’, ‘noetic quality’ and ‘sacredness’), positive mood (awe, joy, peace and tranquillity); transcendence of time and space (sense of being outside of time, in a realm of no space boundaries, sense of timelessness) and ineffability (e.g. inability to adequately describe experience in words). Responses were rated on a 6-point Likert scale ranging from 0 (none, not at all), through to 5 (extreme, more than ever before in my life). The MEQ was administered 1-day post-dose 1 and 2.
- Emotional Breakthrough Inventory (Roseman et al., 2019). The EBI measured emotional breakthrough which occurred during the psilocybin experience with 6 items each rated on a VAS of 1-100 with incremental units of one, with zero defined as ‘no, not more than usually’ and 100 defined as ‘yes, entirely or completely’. The EBI was administered 1-day post-dose 1 and 2.
- Psychological Insight Questionnaire (PIQ; Davis, Barrett, & Griffiths, 2020). This unidimensional self-report questionnaire was developed to assess the intensity of insight experienced during psychedelic dosing session. It consisted of 28 items rated on a 6-point Likert scale, ranging from ‘not at all’ to ‘extremely’. The PIQ was administered 1-day post-dose 1 and 2.
- A set of custom questions assessing 1) respondents’ appraisal of the verisimilitude or 'felt reliability' of their psychedelic experience, 2) key facets of commonly reported high-dose experiences: novelty, particularisation, exogenous absorption, curiosity, and meaningfulness, and 3) relationality (e.g., intimacy, empathy, association) concerning groups with varying 'proximity' (e.g., family, friends, all people, non-human animals). Administered at baseline, 1-day post-dose 1 and 2, 3-weeks post-dose 2, and 20-weeks post-dose 2.
- JHU+ Questions (Griffiths, Richards, McCann, & Jesse, 2006). These questions were based on research by Griffiths and colleagues with two added additional questions. These items asked the respondent to rate, on Likert scales, how (1) personally meaningful, (2) psychologically challenging, (3) psychologically insightful, (4) spiritually significant the psychedelic experience was, and (5) the extent to which they believed the experience improved their wellbeing or life satisfaction. The JHU+ questions was administered at 3-weeks post-dose 2.
- Persisting Effects Questionnaire (PEQ; Griffiths et al., 2006). This measure was used to assess changes in attitudes, moods, behaviours, and spiritual experiences and was developed to be sensitive to the longitudinal effects of psilocybin administration. The PEQ consisted of 60 items, which assessed eight construct domains: positive attitudes about life/self, negative attitudes about life/self, positive mood changes, negative mood changes, altruistic/positive social effects, antisocial/negative social effects, positive behaviour changes, and negative behaviour changes. Items were rated on a six-point scale (0=none, not at all; 1=so slight, cannot decide; 2=slight; 3=moderate; 4=strong; and 5=extreme, more than ever before in your life and stronger than 4). The PEQ were administered at 20-weeks post-dose 2.
- A brief cognitive test battery was used to examine processing speed, verbal fluency, and verbal/visual learning and memory. The battery was administered at baseline, 1-day, 3-weeks and 20-weeks post-dose 2. The battery included the following tasks from the MATRICS (Nuechterlein et al., 2008):
  - Hopkins verbal learning task (HVLT)
  - Brief visuospatial memory test (BVMT)
  - Wechsler memory scale: spatial span III (WMS:SS III)
  - Symbol coding test
  - Verbal fluency test
  - Letter number span

As well as three additional measures of executive function:

- The Stroop Colour-word Interference task – D-KEFS version (Delis, 2001)
  - The Trail-Making Test (Army, 1944)
  - The Brixton spatial Anticipation Test (Burgess & Shallice, 1997)

For consistency, all cognitive tasks were administered by a single trial student with relevant training.

- Cognitive Flexibility Scale (CFS; Martin & Rubin, 1995). This self-report measure assessed three components of cognitive flexibility: awareness of options, willingness to be flexible, and self-efficacy in being flexible. The measure consisted of 12 items rated on a 6-point Likert scale ranging from ‘strongly disagree’ to ‘strongly agree’. The CFS was administered at baseline, 1-day, 3-weeks and 20-weeks post-dose 2.
- Social processing tasks. A short battery of three tasks were used to assess various aspects of social processing, these were administered at baseline, 1-day, 3-weeks and 20-weeks post-dose 2.
  - Multifaceted Empathy Task (MET; Foell, Brislin, Drislane, Dziobek, & Patrick, 2018). A modified version of this widely used task was used to assess the two main components of empathy, cognitive and emotional empathy. It consisted of 40 photographs depicting people in emotionally charged situations. To assess cognitive empathy, respondents were required to select one of four emotion words that matches the emotion in the picture. To assess emotional empathy, respondents were required to rate on a scale from 1-9 ‘How much do you share this person’s emotion right now’.
  - Dynamic emotional Expression Recognition Task (DEER-T; Platt, Kamboj, Morgan, & Curran, 2010). In this task, participants were presented with dynamic face stimuli based on the NimStim Face Stimulus Set, that morph from a neutral expression to extreme emotion over 3000ms. Participants were asked to press one of six keys corresponding to the emotion displayed. Stimuli cover six basic emotions: happiness, neutrality, sadness, anger, disgust, and fear. The primary variable of interest was reaction time.
  - Automatic Imitation Task (AIT; Sowden & Catmur, 2015). This computer based task measured self-other differentiation. In this task, participants were asked to hold down computer keys using their middle and index fingers. The computer screen displayed cues indicating which finger they should raise off the keyboard, as quickly and accurately as possible. Task irrelevant stimuli were also displayed, these were a hand making a movement of some kind. There were 6 types of stimuli used here, these were either baseline stimuli, imitative compatible/incompatible, and spatially compatible/incompatible. Reaction time on successful trials and error rates were the primary outcomes.
- The Vicarious Distress Questionnaire (VDQ; Grynberg, Heeren, & Luminet, 2012). This 12-item self-report questionnaire measured feelings of distress, and behavioural responses of approach and withdrawal in response to encountering another person expressing distress. Statements were rated on a 5-point Likert scale from ‘totally disagree’ to ‘totally agree’. The VDQ was administered at baseline, 1-day, 3-weeks and 20-weeks post-dose 2.
- The Toronto Alexithymia Scale (TAS-20; Bagby, Taylor, & Parker, 1994). This 20-item self-report questionnaire is the most commonly used measure of alexithymia. Each item was rated on a 5-point Likert scale from ‘strongly disagree’ to ‘strongly agree’. Three subscales were derived: difficulties in identifying feelings, difficulties in describing feelings, and externally oriented thinking. The TAS-20 was administered at baseline, 1-day, 3-weeks and 20-weeks post-dose 2.
- Inventory of Interpersonal Problems – short version (IIP-32; Barkham, Hardy, & Startup, 1996). This measure has been widely used to assess a wide range of interpersonal difficulties in studies of therapy-based interventions. It consists of 32 items which required participants/peers to report the extent to which the participant experiences a range of interpersonal problems on a 5-point scale. Eight subscale scores were derived from this: hard to be assertive; hard to be sociable; hard to be supportive; too caring; too dependent; too aggressive; hard to be involved; too open. The IIP-32 self-report was administered at baseline, 3- and 20-weeks post-dose 2. The IIP-32 peer-rated was administered during the preparatory psychotherapy phase once the support person has been assigned, and at 3- and 20-weeks post-dost 2.
- External support questionnaire. A custom set of questions were used to assess the extent and type of mental health support participants resumed and began utilising after the end of the trial treatment up to the long-term follow up. The questions asked whether participants had sought treatment for their mental health from a healthcare professional, the type of healthcare professional (i.e., GP, therapist, psychiatrist, etc.), and the number of times they have seen this person, as well as details around any new medications they have started for the treatment of their mental health disorder(s). The external support questionnaire was administered at 20-weeks post-dose 2.
- Participant feedback. At the end of every online survey, a box was provided for participants to leave any feedback they wished to provide about any aspect of the trial. They were informed that their responses may not be read by trial staff until the end of the trial and if they have any immediate issues, they should contact staff directly. This feedback box was used to gather key information about participant experience of the trial and treatment that may not be capture with other measures. Participant feedback was collected at 1-day pre dose 1 and 2, 1-day, 1-week, and 3-weeks post-dose 1 and 2, and 20-weeks post-dose 2.

Permission for use of all copyrighted questionnaires was obtained where required.

# Safety and Ethics

## Safety measures

These assessments monitored participant safety and well-being throughout the trial and evaluated the intervention's overall risk profile.

- Recent drug use form: All participants were asked to sign a form on the morning of their dosing sessions confirming their abstinence from alcohol and illicit or psychoactive drugs in the last 2 days, antidepressant medication since the start of the trial, and any prescription of non-prescription medication or supplement, other than those already recorded and approved for use during the trial, in the past 7 days.
- Blood tests were conducted at baseline and 1-day post-dose 1 to check for changes in key blood markers of general health in response, in order to ensure participants were healthy and ready to proceed to dosing, and that no adverse physiological response to psilocybin has occurred. A qualified staff member collected an 8.5 mL sample in a serum separating tube (SST) for analysis of electrolytes, urea, creatinine, calcium, magnesium and phosphate, as well as a 10 mL sample in an EDTA tube for a full blood examination. Samples were pre-processed at Swinburne University and then transported to a pathology provider for complete analysis.
- Heart rate and blood pressure measurements. An automatic blood pressure and heart rate monitor was used to monitor participant vital signs throughout dosing sessions. Two recordings were taken prior to administration of psilocybin with the average used as a guide as to whether a participant was safe to proceed with the dosing session. After administration of psilocybin, recordings were taken every 30 minutes for the first two hours. Further recordings were taken if a recording fell outside pre-defined safety thresholds (90/60 mmHg – 160/110 mmHg, 40-110 bpm) or if otherwise clinically indicated.
- Physical contact consent form. . The form documents whether the participant consent to physical contact, whether this can be therapist initiated, the type of physical contact consented to (e.g., hand on shoulder), any specific exceptions (e.g., no hand holding), which therapists may provide therapeutic touch (it may be only one or both), and any specific ‘stop’ words or gestures that will be used to indicate that therapeutic touch should not be provided or should be stopped.
- Physical contact questionnaire. A short set of custom questions was used to gather participant perceptions of therapeutic touch during dosing sessions. Participants will be asked if they consented to therapeutic touch, whether they received touch, whether they thought it was helpful or not, and to provide any feedback they wish about the therapeutic touch they received during dosing sessions. This questionnaire was administered at 3-weeks post-dose 2.
- Hallucinogen Persisting Perceptual Disorder Questionnaire (HPPD-Q; Baggott, Coyle, Erowid, Erowid, & Robertson, 2011). This short questionnaire screens for possible symptoms of Hallucinogen Persisting Perceptual Disorder (HPPD). This measure was administered at the long-term follow-up at 20-weeks post-dose 2. If participants showed symptoms of HPPD, further assessments were made.
- Adverse event (AE) assessment. At every trial event in which participants interacted with trial staff (via phone, video-call or in person), they were asked about adverse events using the following question: *"Have you experienced any new physical or psychological problems since the last assessment/therapy visit, or a worsening of existing problems?".* Further, adverse events that were spontaneously observed by trial staff or reported by participants were also documented. All adverse events were documented using the same form. This form included a full description of the AE, whether the AE related to a pre-existing condition, whether it was solicited or spontaneously reported, and the start/finish time and date of the AE, to be filled out by the staff member who initially discovered the AE. All adverse events were named using the Medical Dictionary for Regulatory Activities (MedRA). Within 24 hours of the initial report, a trial investigator, coordinator or therapist provided the following assessments of the AE: severity, seriousness, relationship to study drug, and expectedness, using definitions outlined in by the Therapeutic Goods Administration (2000). Finally, all actions taken in response to the AE were documented, along with whether the AE resolved, and if unresolved/chronic, the cause of the AE.

## Adverse events

### Definitions

- An **Adverse Event (AE)** is defined as any untoward or unfavourable medical occurrence in a clinical research study participant, including any abnormal sign (e.g. abnormal physical exam or laboratory finding), symptom, or disease, temporally associated with the participant’s involvement in the research, whether or not considered related to participation in the research. This definition includes concurrent illnesses or injuries and exacerbation of pre-existing conditions.
- **Severity**: All adverse events (AE) are to be assessed by a trial investigator for severity and given a rating of ‘mild’, ‘moderate’ or ‘severe’, according to the following
  - *Mild*: No limitation in normal daily activity
  - *Moderate*: Some limitation in normal daily activity
  - *Severe*: Unable to perform normal daily activity
- **Seriousness**: An AE is serious if it meets any of the following criteria:
  - - Results in death
    - Is life-threatening (i.e., the participant was, in the opinion of the investigator, at immediate risk of death from the event as it occurred); it does not refer to an event which hypothetically might have caused death if it were more severe
    - Requires or prolongs inpatient hospitalisation*
    - Results in persistent or significant disability/incapacity (i.e., the event causes substantial disruption of a person’s ability to conduct normal life functions)
    - Results in a congenital anomaly/birth defect
    - Requires intervention to prevent permanent impairment or damage
    - Is an important and significant medical event that may not be immediately life-threatening or resulting in death or hospitalisation, but based upon appropriate medical judgment, may jeopardise the patient/participant or may require intervention to prevent one of the other outcomes listed above.
  - *Hospitalisation for cosmetics, non-emergency prophylaxis or abortion does not result in an SAE report unless, in the view of the investigator, hospitalisation for these procedures was prolonged due to participation in the clinical trial.
  - Based on this assessment, the event is then categorised as either an Adverse Event (AE) or a Serious Adverse Event (SAE).
- **Causality**: The relationship of the study treatment to an AE/SAE is determined based on the following definitions:
  - *Not Related:* The AE is not related if
    - exposure to the investigational product has not occurred; or
    - the occurrence of the AE is not reasonably related in time; or
    - the AE is considered unlikely to be related to use of the investigational product, i.e. there are no facts (evidence) or arguments to suggest a causal relationship; or
    - the AE is more likely related to the participant’s pre-existing condition.
  - *Possibly Related:* The administration of the investigational product and AE are considered reasonably related in time and the AE could be explained by causes other than exposure to the investigational product.
  - *Probably Related:* Exposure to the investigational product and AE are reasonably related in time and the investigational product is more likely than other causes to be responsible for the AE, or is the most likely cause of the AE.
  - If the AE/SAE is determined to be ‘possibly related’ or ‘probably related’, it is then categorised as an Adverse Reaction (AR) or Serious Adverse Reaction (SAR).
- **Expected/unexpected**: All ARs and SARs are to be categorised as expected or unexpected
  - An *unexpected adverse event* is one that is not listed in the current Investigator’s Brochure or an event that is more specific or more severe than a listed event.
    *The Usona Institute Investigator’s Brochure versions 2.0-5.0 were used in this trial.
  - If determined to be unexpected, an AR is then categorised as an Unexpected Adverse Drug Reaction (UADR), and an SAR is then categorised as a Suspected Unexpected Serious Adverse Reaction (SUSAR)

### Reporting procedures

Reporting to the Sponsor

- As an investigator-initiated trial, the sponsor is Professor Susan Rossell at Swinburne University of Technology. All serious adverse events should be reported immediately to the sponsor. Immediate reports should be followed promptly by detailed, written reports.

Reporting to Data Safety Monitoring Board (DSMB)

- All SAEs, SARs, and SUSARs must be reported to the DSMB as soon as possible. A summary of all AEs will be provided to the DSMB at regular meetings.

Reporting to Swinburne HREC:

- All SAEs, SARs, and SUSARs must be reported to the Human Research Ethics Committee (HREC) at Swinburne University as soon as possible. An annual safety report, summarising all AEs will also be submitted to the HREC.

Reporting to the TGA:

- SUSARs must be reported to the Therapeutic Goods Administration (TGA) according to the following:
  - If the SUSAR is fatal or life-threatening, it must be reported to the TGA within 7 calendar days
  - If the SUSAR is *not* fatal or life-threatening, it must be reported to the TGA within 15 calendar days
- The TGA should also be notified of other information that might influence the conduct of the clinical investigation. For example: an increase in the rate of occurrence of an expected SAE which is judged to be clinically important.

Reporting to Usona Institute

- All SAEs, SARs and SUSARs must be reported to USONA within 24 hours of becoming aware of the SAE.
- All adverse events are to be reported in the Annual Safety Update Form.

Reporting to primary physician

- All SAEs, SARs and SUSARs will be reported to the participant's primary physician.

### Follow-up of AEs

- All AEs are to be followed to resolution, or if the AE becomes chronic, a cause identified.
- If an AE is unresolved at the conclusion of the trial, a clinical assessment will be made by the investigator and/or Medical Monitor as to whether continued follow-up of the AE is warranted.
- SAEs are to be followed up to resolution, death or where further information is no longer possible to obtain.

## Risks and risk mitigation

### Psychological risks

Psilocybin given at the 25 mg dose is expected to alter mood, cognition, and perception. Common psychological and adverse effects of psilocybin can include transient anxiety, changes in thought form or thought speed (experiencing thinking as speeding up or slowing down), depersonalisation, derealisation, inattention, impaired concentration, labile mood, altered perception of time, altered visual perception, mild paranoid ideas and unusual thoughts. These are largely transient and reported instances have resolved within six hours (Hasler, et al., 2004; Griffiths, et al., 2016; Ross et al., 2016; Grob, et al., 2011). The likelihood of severe anxiety and distress occurring during a clinically controlled study is much lower than during non-medical or recreational use (Halpern & Pope, 1999; Hasler et al., 2004). Several aspects of the study design minimise the potential for adverse psychological reactions:

- Therapists underwent a professional development training in clinical psychedelic practice.
- Therapists discussed possible effects of psilocybin with participants during preparatory psychotherapy sessions and immediately prior to the dose session to reduce the likelihood of a panic or distressing response. This includes normalising possible sensory changes and possible ‘hallucinogenic’ experiences.
- Therapists taught strategies to manage any anxiety that may arise. Therapists used guided experiential processes to facilitate participants’ rehearsal of non-avoidant responding to potentially distressing experiences. Therapists instructed participants in basic mindfulness interventions to facilitate tolerability and management of intense emotional experiences and reduce subsequent anxiety.
- Therapists conducted a thorough developmental history and formulation prior to the drug dose session. Whilst obtaining a thorough developmental history is usual in psychotherapy practice and important to establish trust and rapport, it also makes trial therapists aware of any past experiences of the participant that may emerge during the psilocybin dose session, or any experiences that may render the participant too psychologically vulnerable to continue participation in the trial.
- The participants were asked to elect a support person to escort them home from dosing sessions. Therapists had a phone conversation with the elected support person to ensure they are an appropriate support person and informed them of how best to support the participant following the dosing session. The procedure was anticipated to protect against psychological adverse events following discharge.
- Participants who were vulnerable to adverse psychological reactions (such as people diagnosed with psychotic disorders or Bipolar I or II) were excluded from study participation.
- Participants who were unable to build adequate levels of rapport and trust with therapists prior to dosing sessions (i.e., those with personality disorders), or who had complex psychiatric histories (i.e., childhood trauma, multiple psychiatric comorbidities, etc.) were excluded from study participation.
- Participants were closely monitored through the duration of the dosing sessions and the rest of the study.
- Trial therapists were available outside of treatment sessions for additional support, by arrangement.
- All participants were required to be currently seeing a healthcare provider regarding their mental health. Contact was made with this healthcare provider prior to participant enrolment in the trial. In the event of an adverse psychological reaction, additional support was provided by this external healthcare provider, where appropriate.

In addition to these measures designed to minimise the risk of psychological harms, the following information were collected throughout the trial to monitor psychological wellbeing:

- Self-report measures of depression and anxiety
- Suicidality – an alert was automatically sent to the trial coordinator and Coordinating Principal Investigator if a participant’s suicidality ratings (QIDS-SR item 12) increased by two or more points or if they reached a score of 3.
- While no cases of Hallucinogen Persisting Perception Disorder (HPPD) have been observed following participation in a clinical trial, participants were screened for HPPD symptoms following trial participation.
- At all points of contact between participants and trial investigators, participants were asked about any possible adverse events.

These safety outcomes were monitored by core trial staff and the Data Safety Monitoring Board at regular intervals during the trial.

### Cardiovascular and sympathomimetic effects

Psilocybin produces slight sympathetic system activation. Physiological effects can include pupillary dilation and detectable but moderate increases in blood pressure or heart rate (Griffiths, et al, 2006; Hasler et al., 2004) and transient nausea, paraesthesia, dizziness, fatigue, and headache. Risks posed by elevated blood pressure were addressed by excluding people with uncontrolled hypertension (high blood pressure) from the trial. Blood pressure (BP) and heart rate (HR) were monitored regularly during the dose sessions using an automatic BP and HR monitor equipped with data logging and alarm functionality. If the automatic BP and HR monitor indicated increasing blood pressure, trial therapists (one of whom was a psychiatrist or psychiatry registrar with medical training) monitored the participant for signs or symptoms of a developing hypertensive or other cardiovascular emergency and took more frequent observations of vital signs.

It was predetermined that in the case of a medical emergency, an ambulance would be called and the participant would be transferred to the Emergency Department of the closest hospital to receive immediate care. Participants would be accompanied by a trial therapist and the coordinating Principal Investigator would be notified.

### Reproductive and Developmental Risks

Pregnant women were excluded from participation. Women who were actively trying to conceive will be excluded from the study. Although there is no evidence that psilocybin is teratogenic or mutagenic, the exclusion from this study of women who can become pregnant is a general ethical commitment. Women of child-bearing potential were asked to use a preapproved method of contraception for the duration of the trial and this was discussed during screening.

### Drug Dependence and Abuse Potential

Findings in human and non-human studies indicate that psilocybin has little to no abuse potential (Fantegrossi et al., 2004). A recent population study indicated that the classical serotonergic psychedelics including psilocybin are not considered to be addictive (Krebs & Johansen, 2013).

### Therapeutic boundaries

Given reports of inappropriate and harmful use of therapeutic touch, as well as boundary violations in a trial of MDMA-assisted psychotherapy (https://www.psymposia.com/powertrip-2/), and the known vulnerability of individuals under the influence of psychedelic substances, a number of precautions were taken in this trial to mitigate the risk of similar issues. These include:

- Therapists training includes thorough discussion of therapeutic touch and therapeutic boundaries.
- All dosing sessions are audio and video recorded. Recordings will be viewed by either the trial coordinator or the principal investigator in the event of a participant or therapist complaint or safety concern.
- All therapists are required to read and sign a physical contact protocol, detailing the use and limitations of therapeutic touch to be used in this trial (Appendix F).
- Participants are provided with a written document outlining the optional use and limitations of therapeutic touch in this trial and who to contact if they have concerns or complaints.
- All direct participant-therapist contact (phone calls, extra sessions, etc.) will be pre-approved and documented by the trial coordinator. Therapists and participants were informed that no contact was to be made outside of the bounds of this trial.

# Data management

## Data collection and secure storage

- Softcopy material:
  - Two REDCap projects were set up, one for screening data, the other for post-enrolment trial data. These contain identifying information. Staff access was limited, such that staff were only granted specific access to the data viewing and entry that they required to carry out their trial responsibilities. Staff accessed REDCap through their personal password protected accounts. Participants accessed online REDCap surveys through personalised links sent to their email. REDCap data is stored on a University of Melbourne virtual server, secured by a firewall, VPN access, and encrypted https connection. REDCap data is centrally backed up nightly.
  - Additionally, a participant scheduling log was created in an excel file. This log contained no identifying information. This file was stored on Google drive with access restricted to necessary staff.
  - Other sources of electronic data such as blood analysis results, screening interview and dosing session recordings were stored on a Swinburne University Cloudstor account. Cloudstor is a secure, password protected cloud service.
  - All electronic data was regularly backed up on a single password-protected solid-state drive which was stored at all times in a locked cabinet.
- Hardcopy material
  - Some cognitive assessments required data to be collected on paper. This data was only labelled with participant ID numbers and contained no identifying information. Any data collected in this manner was immediately entered into REDCap. Hardcopy source files are retained in a locked cabinet.
  - Blood samples were collected and pre-processed at Swinburne University by trial staff. Samples were labelled with participant ID numbers and participant date of birth as a precaution, in case the ID number is smudged or obscured in the process of sample handling. Samples were either stored in a restricted access freezer at Swinburne University or collected by an external pathology provider for analysis. Results from the external pathology provider were sent via a secure portal, accessed only by the trial coordinator, then stored on REDCap/Cloudstor.

## Monitoring, audits and documentation

### Data Entry and Tracking

- Staff accessed REDCap through personal accounts, enabling tracking of data entry. REDCap logs all data entries and edits, automatically maintaining an audit trail.
- Any hardcopy data were initialled by the staff collecting the data. Any changes to source data were traceable without obscuring the original entry. On paper, changes required staff initials and a line through the error.

### Protocol Deviations

- Protocol deviations (e.g., missed assessments) were documented in REDCap with the reason, actions taken, and the staff member’s initials.
- The Trial Coordinator was notified of any deviations. Depending on the severity of deviations, the issue was reported to the coordinating principal investigator (CPI) who investigated the issue and took necessary actions. All protocol deviations were reported to the SUHREC and DSMB.

### Monitoring

- Regular meetings were held with trial investigators to monitor study progress, recruitment rates, follow-up, and any adverse events or protocol issues.
- Trial therapists met regularly with research staff to debrief and ensure the intervention protocol were followed.
- A Data Safety Monitoring Board met regularly to monitor the trial – see section below for more information.
- Participant well-being was continuously monitored, with automatic alerts to staff triggered if there are sudden or severe changes in measures of participant safety and wellbeing.

### Audits

- SUHREC or regulatory authorities could request audits during or after the study, with access to all source documents and protocol files.
- The CPI arranged access for auditors either through a temporary login or by providing printed documents. The CPI had to be available to provide further documents as needed during the audit.

### Reporting

- Annual and final reports were provided to SUHREC and Usona Institute.

### Data disposal

- Once the study was completed, all data was entered and errors resolved, the databases were closed to any further changes. Data was downloaded as the final dataset and sent to the study statistician and relevant staff. At this point, data was removed from REDCap. All materials will be kept for at least 7 years from the publication of results, as per national requirements, and then disposed of by secure destruction methods such as shredding of paper data and overwriting/erasure of computer−generated data.

## Data Safety Monitoring Board (DSMB)

- An independent DSMB was created to oversee the safety monitoring for this study. The DSMB consisted of the three voting members who collectively have experience in the clinical area of interest, statistics and clinical trials. A quorum required at least two members. Trial investigators were not members of the DSMB. The clinical trial coordinator represented the treatment monitoring group (TMG) and was present at all open sessions to take notes and provide meeting minutes/open reports to the DSMB members. In addition, the TMG representative provided meeting minutes and open reports to the Principal Investigator and the rest of the TMG.
- The first DSMB meeting occurred within 12 months of the first participant being enrolled in the trial. Subsequent meetings were conducted every 6 to 12 months, with increased meeting frequency if requested by the DSMB.
- Full details of the DSMB were outlined in the DSMB charter document.
- Reports provided prior to each meeting included:
  - Trial number and title.
  - Brief summary of the trial design and progress.
  - Details of any protocol amendments since the previous report
  - Status of accrual (actual vs target recruitment)
  - If accrual is slower than expected include a plan for increasing enrolment
  - Summary of baseline characteristics
  - Summary of adverse events
  - Summary of serious adverse events
  - Summary of suspected unexpected serious adverse reactions
  - Summary of urgent safety measures
  - Summary of significant safety issues
  - Details of serious breaches
  - Number of protocol deviations requiring exclusion from the per-protocol analysis (such as study treatment discontinuations and, where applicable, withdrawals from study procedures and follow up)
  - Summary of the primary efficacy endpoint
- The DSMB
  - Monitored and reviewed participant safety
  - Reviewed participant recruitment, retention, treatment discontinuation, trial withdrawal, and protocol deviations.
  - Evaluated emerging literature, which may have an impact on the safety or scientific need for the trial
  - Advised on protocol modifications
  - Monitored compliance with the protocol by participants and investigators
  - Monitored compliance with previous DSMB recommendations
- At the end of each meeting the DSMB provided their recommendations regarding continuing, modifying or stopping the trial.

## Ethical approval

This clinical study was designed and was implemented and reported in accordance with the (ICH Harmonised) Australian Therapeutic Goods Administration annotated ‘Note for Guidance on Good Clinical Practice’ (CPMP/ICH/135/95) and with the ethical principles laid down in the Declaration of Helsinki (2000). The protocol and the proposed Participant Information and Consent Form were reviewed and approved by SUT Human Research Ethics Committee before study commencement.

#

# References

(TGA), T. G. A. (2000). *Note for guidance on clinical safety data management: definitions and standards for expedited reporting*.

Army, U. (1944). Army individual test battery. *Manual of directions and scoring*.

Bagby, R. M., Taylor, G. J., & Parker, J. D. (1994). The twenty-item Toronto Alexithymia Scale—II. Convergent, discriminant, and concurrent validity. *Journal of psychosomatic research, 38*(1), 33-40.

Baggott, M. J., Coyle, J. R., Erowid, E., Erowid, F., & Robertson, L. C. (2011). Abnormal visual experiences in individuals with histories of hallucinogen use: A web-based questionnaire. *Drug and alcohol dependence, 114*(1), 61-67.

Barkham, M., Hardy, G. E., & Startup, M. (1996). The IIP‐32: A short version of the Inventory of Interpersonal Problems. *British Journal of Clinical Psychology, 35*(1), 21-35.

Belser, A. B., Agin-Liebes, G., Swift, T. C., Terrana, S., Devenot, N., Friedman, H. L., . . . Ross, S. (2017). Patient experiences of psilocybin-assisted psychotherapy: an interpretative phenomenological analysis. *Journal of Humanistic Psychology, 57*(4), 354-388.

Burgess, P. W., & Shallice, T. (1997). Brixton spatial anticipation test. *Cortex: A Journal Devoted to the Study of the Nervous System and Behavior*.

Carhart-Harris, R., Bolstridge, M., Day, C., Rucker, J., Watts, R., Erritzoe, D., . . . Pilling, S. (2018). Psilocybin with psychological support for treatment-resistant depression: six-month follow-up. *Psychopharmacology, 235*(2), 399-408.

Cipriani, A., Furukawa, T. A., Salanti, G., Chaimani, A., Atkinson, L. Z., Ogawa, Y., . . . Higgins, J. P. (2018). Comparative efficacy and acceptability of 21 antidepressant drugs for the acute treatment of adults with major depressive disorder: a systematic review and network meta-analysis. *Focus, 16*(4), 420-429.

Davis, A. K., Barrett, F. S., & Griffiths, R. R. (2020). Psychological flexibility mediates the relations between acute psychedelic effects and subjective decreases in depression and anxiety. *Journal of Contextual Behavioral Science, 15*, 39-45.

Delis, D. (2001). Delis-Kaplan Executive Function System. *The Psychological Corporation*.

Felitti, V. J., Anda, R. F., Nordenberg, D., Williamson, D. F., Spitz, A. M., Edwards, V., & Marks, J. S. (1998). Relationship of childhood abuse and household dysfunction to many of the leading causes of death in adults: The Adverse Childhood Experiences (ACE) Study. *American journal of preventive medicine, 14*(4), 245-258.

Foell, J., Brislin, S. J., Drislane, L. E., Dziobek, I., & Patrick, C. J. (2018). Creation and validation of an english-language version of the multifaceted empathy test (MET). *Journal of Psychopathology and Behavioral Assessment, 40*, 431-439.

Gámez, W., Chmielewski, M., Kotov, R., Ruggero, C., Suzuki, N., & Watson, D. (2014). The brief experiential avoidance questionnaire: development and initial validation. *Psychological assessment, 26*(1), 35.

Gosling, S. D., Rentfrow, P. J., & Swann Jr, W. B. (2003). A very brief measure of the Big-Five personality domains. *Journal of Research in personality, 37*(6), 504-528.

Griffiths, R. R., Richards, W. A., McCann, U., & Jesse, R. (2006). Psilocybin can occasion mystical-type experiences having substantial and sustained personal meaning and spiritual significance. *Psychopharmacology, 187*(3), 268-283.

Grynberg, D., Heeren, A., & Luminet, O. (2012). Development and validation of the Vicarious Distress Questionnaire. *Canadian Journal of Behavioural Science/Revue canadienne des sciences du comportement, 44*(2), 138.

Haijen, E. C., Kaelen, M., Roseman, L., Timmermann, C., Kettner, H., Russ, S., . . . Lorenz, R. (2018). Predicting responses to psychedelics: a prospective study. *Frontiers in pharmacology, 9*, 897.

MacLean, K. A., Leoutsakos, J. M. S., Johnson, M. W., & Griffiths, R. R. (2012). Factor analysis of the mystical experience questionnaire: a study of experiences occasioned by the hallucinogen psilocybin. *Journal for the scientific study of religion, 51*(4), 721-737.

Martin, M. M., & Rubin, R. B. (1995). A new measure of cognitive flexibility. *Psychological reports, 76*(2), 623-626.

McDermott, B. E. (1995). Development of an instrument for assessing self‐efficacy in schizophrenic spectrum disorders. *Journal of clinical psychology, 51*(3), 320-331.

Montgomery, S. A., & Åsberg, M. (1979). A new depression scale designed to be sensitive to change. *The British Journal of Psychiatry, 134*(4), 382-389.

Nuechterlein, K. H., Green, M. F., Kern, R. S., Baade, L. E., Barch, D. M., Cohen, J. D., . . . Gold, J. M. (2008). The MATRICS Consensus Cognitive Battery, part 1: test selection, reliability, and validity. *American Journal of Psychiatry, 165*(2), 203-213.

Platt, B., Kamboj, S., Morgan, C. J., & Curran, H. V. (2010). Processing dynamic facial affect in frequent cannabis-users: evidence of deficits in the speed of identifying emotional expressions. *Drug and alcohol dependence, 112*(1-2), 27-32.

Roseman, L., Haijen, E., Idialu-Ikato, K., Kaelen, M., Watts, R., & Carhart-Harris, R. (2019). Emotional breakthrough and psychedelics: Validation of the Emotional Breakthrough Inventory. *Journal of Psychopharmacology*, 0269881119855974.

Rush, A. J., Trivedi, M. H., Ibrahim, H. M., Carmody, T. J., Arnow, B., Klein, D. N., . . . Keller, M. B. (2003). The 16-item Quick Inventory of Depressive Symptomatology (QIDS), clinician rating (QIDS-C), and self-report (QIDS-SR): A psychometric evaluation in patients with chronic major depression. In (Vol. 54, pp. 573-583).

Saunders, J. B., Aasland, O. G., Babor, T. F., De la Fuente, J. R., & Grant, M. (1993). Development of the alcohol use disorders identification test (AUDIT): WHO collaborative project on early detection of persons with harmful alcohol consumption‐II. *Addiction, 88*(6), 791-804.

Schulte, D. (2008). Patients’ outcome expectancies and their impression of suitability as predictors of treatment outcome. *Psychotherapy Research, 18*(4), 481-494.

Skinner, H. A. (1982). The drug abuse screening test. *Addictive behaviors, 7*(4), 363-371.

Sowden, S., & Catmur, C. (2015). The role of the right temporoparietal junction in the control of imitation. *Cerebral cortex, 25*(4), 1107-1113.

Spitzer, R. L., Kroenke, K., Williams, J. B., & Löwe, B. (2006). A brief measure for assessing generalized anxiety disorder: the GAD-7. *Archives of internal medicine, 166*(10), 1092-1097.

Studerus, E., Kometer, M., Hasler, F., & Vollenweider, F. X. (2011). Acute, subacute and long-term subjective effects of psilocybin in healthy humans: a pooled analysis of experimental studies. *Journal of Psychopharmacology, 25*(11), 1434-1452.

Tolin, D., Bowe, W., Davis, E., Hannan, S., Springer, K., Worden, B., & Steinman, S. (2016). Diagnostic Interview for anxiety, mood, and OCD and related neuropsychiatric disorders (DIAMOND). *Hartford, CT: The Institute of Living, Hartford Healthcare Corporation*.

Undurraga, J., & Baldessarini, R. J. (2012). Randomized, placebo-controlled trials of antidepressants for acute major depression: thirty-year meta-analytic review. *Neuropsychopharmacology, 37*(4), 851-864.

Watts, R., Kettner, H., Geerts, D., Gandy, S., Kartner, L., Mertens, L., . . . Nutt, D. (2022). The Watts Connectedness Scale: a new scale for measuring a sense of connectedness to self, others, and world. *Psychopharmacology, 239*(11), 3461-3483.

Weekers, L. C., Hutsebaut, J., & Kamphuis, J. H. (2019). The Level of Personality Functioning Scale‐Brief Form 2.0: Update of a brief instrument for assessing level of personality functioning. *Personality and Mental Health, 13*(1), 3-14.

WHO. (1998). Development of the World Health Organization WHO-QoL-BREF quality of life assessment. *Psychological medicine, 28*(3), 551-558.

# APPENDIX A: Study flyer


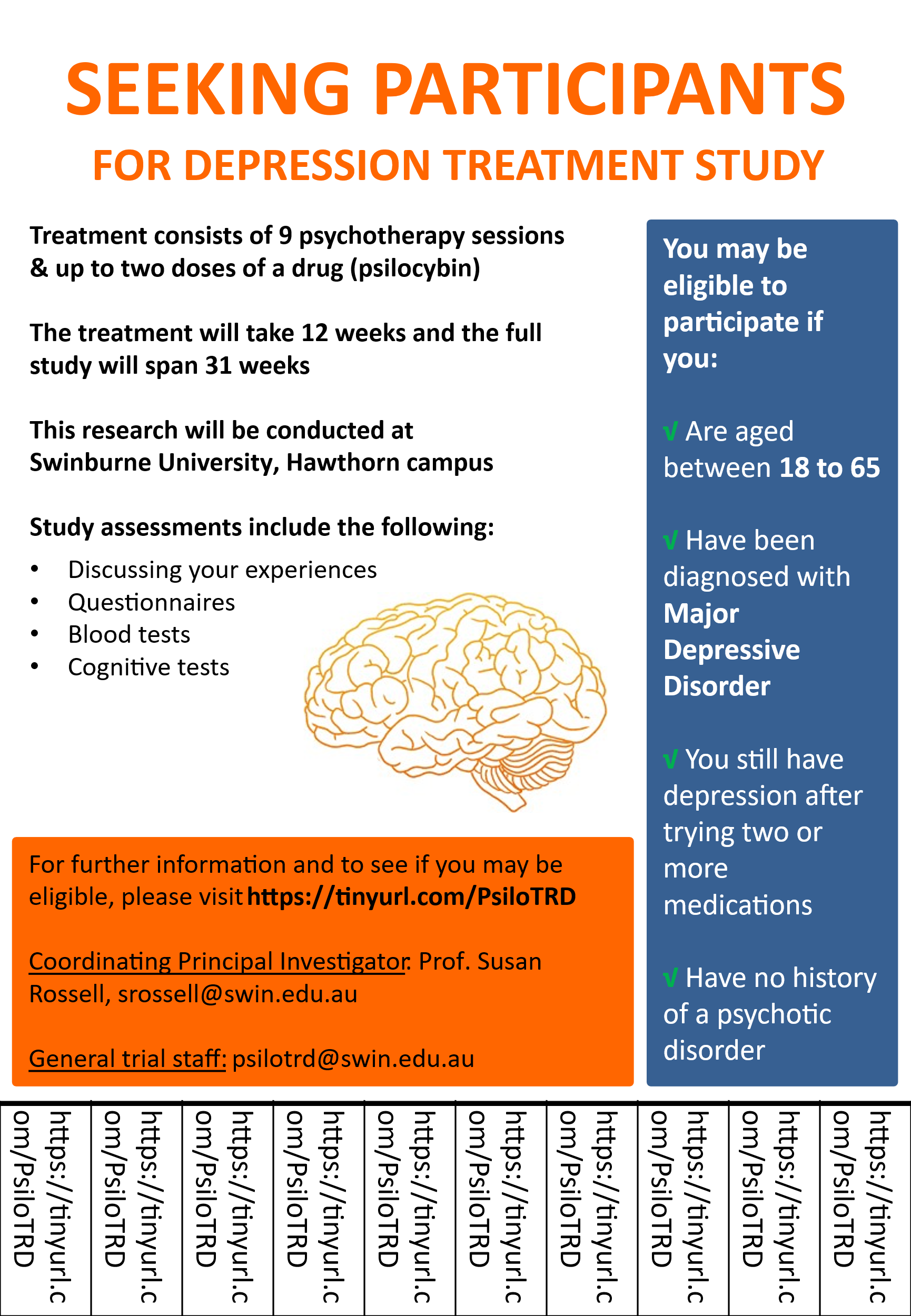


# APPENDIX B: Info for referring practitioners

# APPENDIX C: Participant Information and Consent Form


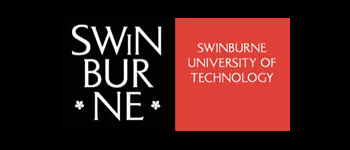


**Participant Information Sheet/Consent Form**

**Interventional Study** - *Adult providing own consent*

Swinburne University, Melbourne

| **Title** | An Open-label Proof of Concept Study of Psilocybin-Assisted Psychotherapy for Treatment-resistant Depression |
| --- | --- |
| **Short Title** | PsiloTRD |
| **Protocol Number** | 20231367-13911 |
| **Project Sponsor** | Investigator-Initiated |
| **Coordinating Principal Investigator/ Principal Investigator** | Prof Susan Rossell |
| **Location** | Swinburne University, VIC |

**Part 1 What does my participation involve?**

**1 Introduction**

You are invited to take part in this research project. This is because you experience depression that has not responded sufficiently to treatments that your clinical team have recommended. This includes trying at least two different medications that are typically prescribed for depression. This project is testing a new treatment for depression. The new treatment requires you to participate in a treatment program that consists of multiple sessions of a specialised form of psychotherapy and one or two doses of a drug called psilocybin. This treatment approach, called ‘psilocybin-assisted psychotherapy’, is described in more detail below.

This Participant Information Sheet/Consent Form tells you about the research project. It explains the tests and treatments involved. Knowing what is involved will help you decide if you want to take part in the research.

Please read this information carefully. Ask questions about anything you don’t understand or want to know more about. Before deciding whether or not to take part, you might want to talk about it with a relative, friend, or your doctor.

Participation in this research is voluntary. If you don’t wish to take part, you don’t have to. You will receive the best possible care whether or not you take part.

If you decide you want to take part in the research project, you will be asked to sign the consent section. By signing it you are telling us that you:

• Understand what you have read

• Consent to take part in the research project

• Consent to have the tests and treatments that are described

• Consent to the use of your personal and health information as described

You will be given a copy of this Participant Information and Consent Form to keep.

**2 What is the purpose of this research?**

Up to one-third of adults experiencing depression have symptoms that do not respond to treatment with available antidepressant medications. These patients may be referred to as having ‘treatment-resistant’ depression (MDD-TR). Psilocybin-assisted psychotherapy is an experimental intervention that could improve the symptoms of depression. This study will determine whether this new treatment is safe and effective in treating MDD-TR. In addition, the study will take a number of measures to see how this treatment works.

Psilocybin, the drug used in psilocybin-assisted psychotherapy, is not approved for MDD-TR, and therefore it is an experimental treatment.

The study Chief Investigator is Professor Susan Rossell. This research has been funded by the Barbara Dicker Brain Sciences Foundation and Usona Institute.

Data collected in this trial will contribute to the PhD projects of Ms Sally Meikle and Ms Lauren Johansen and the honour project of Ms Tess Douglass.

**3 What does participation in this research involve?**

You will be participating in a single-arm open-label research project. This means all participants receive the same treatment and are made aware of what this treatment is.

If you agree to participate in this study, you will be asked to sign this consent form before any study assessments are performed. All of this research will take place at Swinburne University of Technology, Hawthorn.

Screening

Prior to the completion of any assessments, the study investigators will make sure you are eligible to participate. To do this, we will invite you to complete an online survey, and if eligible, to attend an on-site clinical interview with a study doctor. In this interview the doctor will ask about your history of depression, any substances you use, other mental health details, as well as perform a basic physical exam that includes questions, and measurement of height, weight, heart rate, blood pressure, electrocardiogram (ECG), and a blood draw to assess liver and kidney function. The doctor will also contact your regular treating physician to discuss your potential participation and request any relevant medical history/records. After these assessments, the study investigators will assess whether you may eligible to participate in this study. To be eligible you will need to a) have a confirmed diagnosis of Treatment Resistant Major Depressive Disorder, b) currently experience depression, and c) not have one of a number of conditions which would prevent participation. If you are not eligible, one of our study investigators will explain this to you.

Washout

If you are currently taking any antidepressant medications, you are likely to be required to stop taking these to participate in this study. This is because it may not be safe to take medications in combination with the study drug. If you are taking one of these medications, this will be identified during the initial phase of screening, prior to your participation in the study. If you have not been deemed ineligible during the initial phase of screening and would like to stop taking this medication and participate, and your prescribing physician determines it would be safe to do so, you will be given a timetable to reduce and eventually stop taking this medication. This will be achieved under the supervision of your prescribing physician. You will not be enrolled in the study unless you have withdrawn from these medications.

Psilocybin-assisted psychotherapy

In this study, you will receive a combination of a drug (psilocybin) and psychotherapy. This drug is predicted to work differently from other drugs used to treat depression. Rather than being taken every day, it is used in combination with psychotherapy on a couple of occasions.

You will receive three psychotherapy sessions before your first psilocybin dose to prepare you to get the most out of your psilocybin session. On the dosing day, you will take a 25 mg dose of psilocybin at the beginning of the session, which will last approximately 6-8 hours. Psilocybin is a psychoactive drug that usually produces substantial changes to your perception, feelings, and thinking that will last for several hours. The same two trial therapists will facilitate the treatment program, including the psychotherapy and psilocybin sessions. Both therapists will remain with you throughout the entire psilocybin session and will monitor your safety and wellbeing. You will also receive three psychotherapy sessions after the initial psilocybin session, during which you can talk through your experiences and any effects it had on you and your condition. Six weeks after your first psilocybin session you will be invited to come to a second psilocybin session, which will follow the same format as the first session. You can choose whether or not to have this second psilocybin session at that time. Whether you do or do not attend a second psilocybin-assisted psychotherapy session, you will receive a further three psychotherapy sessions.

On the days that you receive psilocybin, you will be asked to have a support person / family member accompany you home. If they do not have their own transportation, a taxi voucher will be provided.

Assessments

Assessments of your symptoms and other measurements will be taken at different time points across the study. Given the nature of this study, multiple assessments are required. For ease of understanding, this is presented in the table below. Each assessment will be explained to you in detail by our team and will be scheduled at a suitable time for all parties within the trial requirements. Please consider the time commitment carefully before you volunteer to take part.

| **Study phase** | **What?** | **Time requirement** |
| --- | --- | --- |
| Screening | Clinical interview; questionnaire; general physical exam; blood sample | 2.5 hours |
| Baseline | Online survey; cognitive tasks; blood sample; qualitative interview | 4 hours |
| Preparatory psychotherapy | - | 3 x 1.5-hour session |
| 1 day before dose 1 | Online survey | 5 minutes |
| Dose 1 | Administration of psilocybin under therapist supervision | 6-8 hours |
| 1 day after dose 1 | Online survey | 30 minutes |
| Integrative psychotherapy | - | 3 x 1.5-hour session |
| 3 weeks after dose 1 | Online survey | 30 minutes |
| 1 day before dose 2 | Online survey | 5 minutes |
| Dose 2 | Administration of psilocybin under therapist supervision | 6-8 hours |
| 1 day after dose 2 | Online survey; cognitive tasks; blood sample | 1 hour |
| Integrative psychotherapy | - | 3 x 1.5-hour session |
| 9 weeks after dose 1 | Online survey; cognitive tasks; blood sample; qualitative interview | 3.5 hours |
| 26 after dose 1 (long-term follow up) | Online survey; cognitive tasks; blood sample; qualitative interview | 4 hours |

During the baseline assessment you will be asked questions about your condition in an interview with a trial investigator, and you will complete a number of questionnaires about your symptoms, as well as cognitive tasks (simple attention and memory measures). You will also be asked to provide a blood sample and participate in a qualitative interview. These assessments are repeated at 9 and 26 weeks after the first psilocybin dose. There will also be several online surveys through the study that you will fill out at home.

Each psilocybin dosing session will be recorded on video. These recordings may be used to monitor the way therapists are conducting therapy, and (subject to your written approval) to teach future therapists how to conduct this type of therapy. Qualitative interviews will also be recorded, to ensure all the information given during these interviews is captured. These recordings will not be used outside the context of this research without your written approval.

In total, the duration of your participation in this study is estimated to be about 33 weeks.

There are no costs associated with participating in this research project. All medication, tests and medical care required as part of the research project will be provided to you free of charge. You will be reimbursed $200 for your time and travel expenses.

If you decide to participate in this research project, the study researcher will inform your primary care team.

**4 What do I have to do?**

To participate in this study, you will be required to cease taking your antidepressant medications, if you are currently being prescribed any. If you consent to participate, supervised reductions of your antidepressant will be conducted until you are no longer taking this medication. This may take several weeks depending on the dose and type of medication you are taking. You will not be able to take any antidepressants prior to the psilocybin sessions, and for the duration of the treatment program (12 weeks). If you change your medication at any point during the study, you are required to let the study investigators know. You will not be able to take any herbal supplements or any additional prescription or non-prescription medications outside of your usual and allowed prescribed medications and dose, for the week before the psilocybin session. You will be required to refrain from using any other psychoactive drug within 48 hours of the session. You will be unable to smoke during the psilocybin dosing sessions.

In relation to the COVID -19 pandemic, the University needs to comply with Government requirements in relation to vaccination and contact tracing.

This study involves several face-to-face appointments on campus. Therefore, to participate in this study, individuals will need to be fully vaccinated, or have a government approved medical exemption. During campus visits, the University may need to (a) disclose your presence on campus and (b) sight information relating to your vaccination status. Please discuss any concerns about this disclosure process with the researchers hosting your visit on campus as you make your decision to participate in research activities at Swinburne.

**5 Other relevant information about the research project**

15 adults diagnosed with Major Depressive Disorder that is considered treatment-resistant will take part in the study. All participants will be recruited from Melbourne. All participants will receive the trial intervention – psilocybin-assisted psychotherapy.

**6 Do I have to take part in this research project?**

Participation in any research project is voluntary. If you do not wish to take part, you do not have to. If you decide to take part and later change your mind, you are free to withdraw from the project at any stage.

Your decision whether to take part or not to take part, or to take part and then withdraw, will not affect your routine treatment, your relationship with those treating you or your relationship with your treatment service.

**7 What are the alternatives to participation?**

You do not have to take part in this research project to receive treatment. Other options are available; these include remaining on any anti-depressant medications you are being prescribed or continuing with any psychotherapy or treatments you are receiving. Your study researcher will discuss these options with you before you decide whether or not to take part in this research project. You can also discuss the options with your primary care team.

**8 What are the possible benefits of taking part?**

We cannot guarantee or promise that you will receive any benefits from this research; however, possible benefits may include an improvement in your symptoms and quality of life.

**9 What are the possible risks and disadvantages of taking part?**

**Psilocybin**

Psilocybin is a psychoactive drug. A 25 mg dose is expected to cause substantial changes in your thinking, feeling and perception. These changes can sometimes feel strange or unpleasant. Adverse psychological effects can include temporary anxiety or psychological distress, changes in thoughts, impaired concentration, altered perception of time, altered visual perception, and mild paranoia. Trial therapists will be available to assist in minimising any distress caused by the psilocybin. If these side effects do occur, they usually last for a short period of time.

Psilocybin may also cause physical side effects, including minor increases in blood pressure and heart rate. Both blood pressure and heart rate will be monitored throughout the session.

Many side effects go away after the drug wears off. However, very rarely side effects can be serious, long lasting, or permanent. If a severe side effect occurs, the trial investigators may need to administer a medication to reverse or counteract the effects of the psilocybin or transfer a participant to a hospital for further medical care.

If any long-lasting psychological adverse effects occur, these will be managed in the psychotherapy sessions after the psilocybin session. If participants wish, they may schedule additional sessions with the trial therapists. Trial therapists may provide extra sessions if necessary, and subject to availability. They may also refer participants to other relevant mental health care services.

**Blood Taking**

Having blood taken may cause some discomfort, bruising, minor infection or bleeding. If this happens, it can be easily treated.

**Pregnancy**

The effects of psilocybin on the unborn child and on the newborn baby are not known. Because of this, it is important that research project participants are not pregnant or breast-feeding and do not become pregnant during the course of the research project. You must not participate in the research if you are pregnant or trying to become pregnant, or breast-feeding. If you are female and childbearing is a possibility, you may be required to undergo a pregnancy test prior to each drug dosing session.

If you do become pregnant whilst participating in the research project, you should advise your study researcher immediately. Your study researcher will withdraw you from the research project and advise on further medical attention should this be necessary, and you will be asked to provide us with some medical information about the pregnancy. You must not continue in the research if you become pregnant.

**10 What will happen to my test samples?**

Blood samples will be collected from all participants. The purpose of these blood samples is to check key organ function prior to psilocybin dosing, and to investigate how psilocybin works. By consenting to participate in this study, you are consenting for your blood samples to be taken, stored, tested and analysed for research purposes.

During the study, your blood samples will be labelled with your individual participant code and your date of birth. This ensures samples are not mixed up. Privacy and confidentiality will be maintained by keeping samples in a secure storage facility. At the completion of the study, the samples will be entered into a databank in a de-identified manner, and stored indefinitely. De-identified meaning that the data can no longer be traced back to the study participant who provided the sample. If you do not wish for your data to be stored indefinitely in the databank, you will need to indicate this by ticking the appropriate box in the consent form.

Samples of your blood obtained for the purpose of this research project will be stored at Swinburne University.

**11 What if new information arises during this research project?**

Sometimes during the course of a research project, new information becomes available about the treatment that is being studied. If this happens, your study researcher will tell you about it and discuss with you whether you want to continue in the research project. If you decide to withdraw, your study researcher will make arrangements for your regular health care to continue. If you decide to continue in the research project you will be asked to sign an updated consent form.

Also, on receiving new information, your study researcher might consider it to be in your best interests to withdraw you from the research project. If this happens, he/ she will explain the reasons and arrange for your regular health care to continue.

**12 Can I have other treatments during this research project?**

While you are participating in this research project, you may not be able to take some or all of the medications or treatments you have been taking for your condition or for other reasons. It is important to tell your study researcher and the study staff about any treatments or medications you may be taking, including prescription and non-prescription medications, vitamins or herbal supplements. You should also tell trial investigators about any changes to these during your participation in the research project. Your study researcher will explain which treatments or medications need to be stopped for the time you are involved in the research project. During this trial you will be able to see your regular mental healthcare providers for support.

**13 What if I withdraw from this research project?**

If you decide to withdraw from the project, please notify a member of the research team before you withdraw. This notice will allow that person or the research supervisor to discuss any health risks or special requirements linked to withdrawing.

If you do withdraw your consent during the research project, the study researcher and relevant study staff will not collect additional personal information from you, although personal information already collected will be retained to ensure that the results of the research project can be measured properly and to comply with law. You should be aware that data collected up to the time you withdraw will form part of the research project results. If you do not want them to do this, you must tell them before you join the research project.

If you withdraw from the study for any reason after drug administration, the investigator will review your medications and record any adverse events, where possible.

**14 Could this research project be stopped unexpectedly?**

This research project may be stopped unexpectedly for a variety of reasons. These may include reasons such as:

• Unacceptable side effects

• The drug/treatment being shown not to be effective

**15 What happens when the research project ends?**

A summary of the general findings of this research project will be made available to all participants via post or email at the study conclusion, if they have indicated consent to receive such further communication.

**Part 2 How is the research project being conducted?**

**16 What will happen to information about me?**

By signing the consent form you consent to the study researcher and relevant research staff collecting and using personal information about you for the research project, this includes accessing your personal medical records. Any information obtained in connection with this research project that can identify you will remain confidential.

In the handling of data, all references to personal information will be removed and replaced by a code, so that participants will not be able to be individually identified. Blood samples will be the only exemption from this, as they will be labelled with each participant’s individualised code and their date of birth. This ensures that samples are not mixed up, in case the participant code is incorrectly labelled or smudged. All data will be stored securely, under lock-and-key, or via password protection, at the research venue. Access to the data will only be available to the principal researchers and the supervised trial coordinators/research assistants responsible for this study.

Your information will only be used for the purpose of this research project and it will only be disclosed with your permission, except as required by law.

It is anticipated that the results of this research project will be published and/or presented in a variety of forums. In any publication and/or presentation, information will be provided in such a way that you cannot be identified, except with your permission. All participants will remain anonymous.

Results may be presented both in aggregate form and at an individual level.

Where information is presented in aggregate form, it will be summarised, for example, as an average or a range of scores, for the whole sample.

Where information is presented at an individual level, a number of pieces of information (such as demographic information or scores on an assessment) for each individual participant will be tied together and presented as an individual participant profile. Identifying personal information (such as name, date of birth, and contact information) will not be used. Each participant will be given a pseudonym or a separate participant ID number to protect participant identity. The reporting of information will be limited to what is deemed necessary for presenting each case in enough detail to be clinically useful. Personal and sensitive information, where possible, will be obscured, for example through using ranges or approximations (i.e., reporting age as 30-40 years, rather than 37 years). Information that may be presented individually includes sex; age; depression history; demographics; psychiatric comorbidities; scores on key outcome measures; short quotes from qualitative interviews with each participant; and short descriptions of the effects of the treatment based on the data collected.

In accordance with relevant Australian and/or State privacy and other relevant laws, you have the right to request access to your information collected and stored by the research team. You also have the right to request that any information with which you disagree be corrected. If you would like to review your individualised data before it is published, please tick the relevant box when signing the consent sheet at the end of this document. We will then send you a draft version of these results before they are published; you will be able to review this draft and provide feedback, correct information you disagree with, or redact information you do not want published.

Any information obtained for the purpose of this research project and for the future research described in this section that can identify you will be treated as confidential and securely stored. It will be disclosed only with your permission, or as required by law.

All of the data collected as part of this study (including blood samples) will be stored indefinitely in a databank. By signing the consent form, you are consenting to participate in this study and for your data to be used in a de-identified format in future research. If you do not wish for your data to be stored indefinitely in the databank, you will need to indicate this by ticking the appropriate box in the consent form.

**17 Complaints and compensation**

If you suffer any injuries or complications as a result of this research project, you should contact the study team as soon as possible and you will be assisted with arranging appropriate medical treatment. If you are eligible for Medicare, you can receive any medical treatment required to treat the injury or complication, free of charge, as a public patient in any Australian public hospital.

In the event of loss or injury, the parties involved in this research project have agreed to assist you with arranging appropriate medical treatment.

**18 Who is organising and funding the research?**

This research project is being conducted by a team of clinical scientists and doctors from Melbourne, Australia and London, United Kingdom. The Chief Principal Investigator is Prof. Susan Rossell.

This research project is being funded by Barbara Dicker Brain Sciences Foundation and Usona Institute.

**19 Who has reviewed the research project?**

All research in Australia involving humans is reviewed by an independent group of people called a Human Research Ethics Committee (HREC). The ethical aspects of this research project have been approved by the HREC of Swinburne University, Melbourne.

This project will be carried out according to the *National Statement on Ethical Conduct in Human Research (2007)*. This statement has been developed to protect the interests of people who agree to participate in human research studies.

**20 Further information and who to contact**

The person you may need to contact will depend on the nature of your query. If you want any further information concerning this project or if you have any medical problems which may be related to your involvement in the project (for example, any side effects), you can contact any of the following people:

**Coordinating Principal Investigator**

| Name | Prof Susan Rossell |
| --- | --- |
| Telephone | XXX |
| Email | XXX |

**Trial Coordinator**

| Name | Sally Meikle |
| --- | --- |
| Telephone | XXX |
| Email | XXX |

**Clinical contact person**

| Name | Peter Bosanac |
| --- | --- |
| Telephone | XXX |
| Email | XXX |

When you enrol in the study, we will also provide you with a contact number to call your treating therapist during the 24hours following each psilocybin session, should this be needed.

If you have any complaints about any aspect of the project, the way it is being conducted or any questions about being a research participant in general, then you may contact:

**Reviewing HREC approving this research** **and HREC Executive Officer details**

| Position | HREC Executive Officer |
| --- | --- |
| Telephone | +61 3 9214 3845 or +61 3 9214 8145 |
| Email | [resethics@swinburne.edu.au](mailto:resethics@swinburne.edu.au) |

**Consent Form -** *Adult providing own consent*

| **Title** | An Open-label Proof of Concept Study of Psilocybin-Assisted Psychotherapy for Treatment-resistant Depression |
| --- | --- |
| **Short Title** | PsiloTRD |
| **Protocol Number** | 20201367-3726 |
| **Project Sponsor** | Investigator-initiated |
| **Coordinating Principal Investigator/**  **Principal Investigator** | Prof Susan Rossell |
| **Location** | Swinburne University, VIC |

**Declaration by Participant**

I have read the Participant Information Sheet or someone has read it to me in a language that I understand.

I understand the purposes, procedures and risks of the research described in the project.

I give permission for my doctors, other health professionals, hospitals or laboratories outside this hospital to release information to Swinburne University concerning my condition and treatment for the purposes of this project. I understand that such information will remain confidential.

I have had an opportunity to ask questions and I am satisfied with the answers I have received.

I freely agree to participate in this research project as described and understand that I am free to withdraw at any time during the study without affecting my future health care.

I understand that, if I decide to discontinue the study treatment, I may be asked to attend follow-up visits to allow collection of information regarding my health status. Alternatively, a member of the research team may request my permission to obtain access to my medical records for collection of follow-up information for the purposes of research and analysis.

I understand that I will be given a signed copy of this document to keep.

Please tick this box if you want your data used ONLY for the purpose of this project,

And DO NOT want your data stored indefinitely in a databank.

Please tick this box if you would like to review your individualised data before it is published.

|  | | | | | | |
| --- | --- | --- | --- | --- | --- | --- |
|  | Name of Participant (please print) | |  |  |  |  |
|  | | | | | | |
|  | Signature |  | | Date |  |  |
|  | | | | | | |

**Declaration by Study Doctor/Senior Researcher^†^**

I have given a verbal explanation of the research project, its procedures and risks and I believe that the participant has understood that explanation.

|  | | | | | | |
| --- | --- | --- | --- | --- | --- | --- |
|  | Name of Study Doctor/  Senior Researcher^†^ (please print) | |  | | |  |
|  | | | | | |  |
|  | Signature |  | | Date |  |  |
|  | | | | | | |

^†^ A senior member of the research team must provide the explanation of, and information concerning, the research project.

Note: All parties signing the consent section must date their own signature.

**Form for Withdrawal of Participation -** *Adult providing own consent*

| **Title** | An Open-label Proof of Concept Study of Psilocybin-Assisted Psychotherapy for Treatment-resistant Depression |
| --- | --- |
| **Short Title** | PsiloTRD |
| **Protocol Number** | 20201367-3726 |
| **Project Sponsor** | Investigator-initiated |
| **Coordinating Principal Investigator/**  **Principal Investigator** | Prof Susan Rossell |
| **Location** | Swinburne University, VIC |

**Declaration by Participant**

I wish to withdraw from participation in the above research project and understand that such withdrawal will not affect my routine treatment, my relationship with those treating me or my relationship with Swinburne University

|  | | | | | | |
| --- | --- | --- | --- | --- | --- | --- |
|  | Name of Participant (please print) | |  |  |  |  |
|  | | | | | | |
|  | Signature |  | | Date |  |  |
|  | | | | | | |

|  |
| --- |

**Declaration by Study Doctor/Senior Researcher^†^**

I have given a verbal explanation of the implications of withdrawal from the research project and I believe that the participant has understood that explanation.

|  | | | | | | |
| --- | --- | --- | --- | --- | --- | --- |
|  | Name of Study Doctor/  Senior Researcher^†^ (please print) | |  | | |  |
|  | | | | | |  |
|  | Signature |  | | Date |  |  |
|  | | | | | | |

^†^ A senior member of the research team must provide the explanation of and information concerning withdrawal from the research project.

Note: All parties signing the consent section must date their own signature.

# **APPENDIX D**: Participant Information

**PsiloTRD Participant Information**

An Open-label Proof of Concept Study of Psilocybin-Assisted Psychotherapy for Treatment-resistant Depression

This document contains information about your participation in the PsiloTRD trial. This information is intended to supplement the information already provided in the Participant Information Sheet/Consent form which outlines what the trial involves and the risks associated with participation.  If you no longer have a copy of this and wish for another one, please ask us.

**Trial treatment**

Therapists:

- All trial treatment will be conducted by a team of two therapists. Both therapists will attend every session with you.

Therapy sessions:

- Preparatory psychotherapy: You will receive three preparatory psychotherapy sessions prior to your first dosing session. These sessions will help you prepare for and get the most out of your dosing session(s).
- Dosing sessions: At each dosing session, you will take a 25 mg dose of psilocybin at the beginning of the session. The effects of the drug will last approximately 6-8 hours. Your therapists will remain with you throughout the entire psilocybin session and will monitor your safety and wellbeing. The trial treatment involves two dosing sessions, six weeks apart. The second session is optional but encouraged, you can discuss whether you wish to participate in this second dosing session with your therapist team.
- Integrative psychotherapy: You will also receive six integrative psychotherapy sessions. These sessions occur after dosing sessions. During these sessions you can talk through your experiences and any effects it had on you and your condition.

Physical contact with therapists:

- During dosing sessions, some people find it comforting or useful to receive physical contact from their therapists (e.g., having a therapist hold their hand).
- Before the dosing sessions, in the preparatory psychotherapy sessions, your therapists will discuss physical contact with you.
- Your therapists will ask you:
  - Whether or not you would like the option of physical contact during dosing sessions
  - What kind of physical contact you approve (i.e., hand holding or a hand on your shoulder) and any exceptions
  - Whether you would like therapists to initiate physical contact or whether you would only like physical contact to be given after you explicitly request it (i.e., would you like them to hold your hand if you appear distressed, or would you like them to wait for you to ask them to hold your hand)
  - Which therapist you approve of physical contact with (one, both or neither)
  - How you will indicate to the therapists that you do or do not want physical contact during the dosing session (i.e. words or gestures you might use)
- Any physical contact you consent to and the exceptions to this will be practiced and documented in the preparatory psychotherapy sessions.
- Things to remember
  - This physical contact is entirely optional and you can revoke your consent to physical contact at any time, without giving a reason.
  - Physical touch will not occur outside of dosing sessions, or practicing the physical touch in preparatory psychotherapy sessions.
  - This physical touch should never be sexual or sensual. It should not make you feel uncomfortable. Only the touch that you agree to should be provided.

Contact with therapists outside of scheduled sessions:

- Your therapist team will be on-call for 24 hours following each dosing session, should any urgent issues arise that you wish to discuss this them. This number will reach the trial coordinator who will forward the call to your therapist. Please note, this number is only to be used for participant support in the 24 hours following your dosing session.
  - Phone: **XXXX XXXX**
- In rare cases, if a participant requires further support from their therapists outside of the scheduled sessions, additional therapy sessions may be provided, subject to therapist availability.

Support person:

- After each dosing session, we ask that you have a close friend or family member (a ‘support person’) accompany you home and stay with you for 24 hours.
- This support person should be someone who you know well, feel comfortable with and is supportive of your mental health and participation in the trial.
- During one of the preparatory psychotherapy sessions, your therapists will ask you to elect someone to be your support person. They will then contact them to provide them with some information about this therapy and how best to support you following dosing sessions.
- We will also ask your support person to participate in a brief questionnaire as part of this study. This questionnaire will ask about your mental health from their perspective. This will help us validate the effects of this therapy. They do not have to participate in this aspect of the trial if they do not want to, and this will not impact their ability to be your ‘support person’.

**External treatment**

- Participants are encouraged to continue to regularly see their external treating mental healthcare professionals (e.g., your psychologist, psychiatrist, therapist, GP, etc.). These people may provide additional support as you progress through this trial, and upon completion of the trial, should you require further support.
- We ask that you do not start any new therapies (i.e., starting a new form of psychotherapy, such as CBT, that you were not previously undergoing) or start seeing any new mental healthcare professionals (i.e., finding a new therapist) while you participate in this trial.
- We ask that you do not start taking any new medications for the duration of the treatment phase of the trial. If new medications are indicated by your medical team please inform us and we can discuss with you how this might influence your involvement in the trial and dosing sessions.

**Trial assessments**

- There are a range of assessment events throughout this study that you need to attend.
  - Surveys: these are online surveys that will be emailed to you on the day you need to complete than. You will complete these online, on the day they are scheduled.
  - In-person assessments: These are trial assessments, such as cognitive tests, blood tests, etc. that need to be completed in-person with trial staff at Swinburne University.
  - Interviews: These are structured interviews with trial staff that will be conducted via zoom.
- When completing these assessments, it is important that you are honest with us. Sometimes participants feel pressure or responsibility to report what they think the researchers want to hear. However, the best thing you can do, for us and for yourself, is to be completely honest in your reporting. You will not be removed from the trial due to your responses on our assessments, unless it is determined it is unsafe for you to continue participating in the trial.
- Sometimes participants may also answer quickly without reflecting on how they really feel about something or determining what they really think. It can be helpful to take a moment before answering to reflect what a question is asking and how you think/feel before proceeding.

**COVID-19 regulations**

To participate in this trial, you must:

- Complete a rapid antigen test on the afternoon prior to each dosing session. These will be provided to you during a preparation psychotherapy session. If the result is positive, you must inform the trial coordinator immediately via phone call/text. If the result is negative, you must take a photo of the test and present it to the therapists at your dosing session the next day.

To further minimise the risk of any COVID-19 related disruptions we ask that you:

- Monitor yourself for symptoms of COVID-19 prior to any on-site visit. If you develop symptoms, please test yourself. Common symptoms include:
  - Fever
  - Cough or sore throat
  - Tiredness
  - Loss of taste or smell
- Test yourself if you are a close contact of someone who has tested positive for COVID-19.
- Inform us if you test positive to COVID-19 at any point throughout the trial.
- Minimise your risk of contracting COVID-19 throughout the trial. Strategies include:
  - Wearing a mask in crowded, indoor spaces
  - Avoiding contact with people who may have COVID-19
  - Regularly washing your hands
  - Staying up-to-date with your vaccinations

**Your responsibilities**

As a participant in this clinical trial, you are expected to:

- Follow instructions provided to you by trial staff.
- Attend all sessions and complete surveys in a timely manner. If you are unable to do so, you must let us know ASAP. In some cases, adjustments may be made, however this is not guaranteed. It is expected that participants in this trial will make adjustments to their regular activities in order to attend all trial events.
- Communicate with trial staff in a timely manner, check emails regularly and respond within a reasonable time frame. Many important updates and trial information will be provided via email, thus it is important that you regularly check your emails.
- Inform us of any changes to your health or the medications you take throughout the trial. We ask that you do not start taking new medications without informing us, for the duration of the treatment phase of the trial.  If you do take any medication that has not been approved by trial staff, it is important that you inform us. Some medications can interact with the study drug; for your safety it is important we are aware of everything you have taken recently.

**Who to talk to**

Feedback

There will be regular points during the trial that we ask for your feedback. At the end of each online survey there will be a place for you to provide feedback about any aspect of the trial. Please note that this feedback won’t be viewed until after the trial is complete. If you have feedback and wish to discuss it with someone or an immediate concern, please contact the trial coordinator.

Trial coordinator

The trial coordinator, Sally Meikle, is the primary point of contact for any general questions or concerns about any aspect of the trial. Please use email to contact her, unless your query is urgent (i.e., relating to an in-person event in the next 24 hours).

- Email: [psilotrd@swin.edu.au](mailto:psilotrd@swin.edu.au)
- Phone: XXXX XXXX

Coordinating Principal Investigator

If preferred, the coordinating principal investigator, Professor Susan Rossell, may be contacted.

- Email: [xxxxxxxxxxxxxxxx](mailto:srossell@swin.edu.au)
- Phone: XXXX XXXX

Human Research Ethics Committee (HREC)

If you have any complaints about any aspect of the project, the way it is being conducted or any questions about being a research participant in general, then you may contact the HREC.

- Email: [resethics@swinburne.edu.au](mailto:resethics@swinburne.edu.au)
- Phone: 9214 3845 or 9214 8145

**Additional Psychological Support**

- If you require immediate psychological support, outside of the 24 hours after a dosing session, you should contact one of the following:
  - *Beyond Blue*

Phone: 1300 22 4636

Email and chat help: see www.beyondblue.org.au

- *Lifeline*

Phone: 13 11 14

Text: 0477 13 11 14

Online chat: www.lifeline.org.au/crisis-chat

- *Suicide Line*

Phone: 1300 651 251

- **If you are in immediate need of medical help, please dial 000 and ask for an Ambulance.**
- It is important to tell any health practitioner treating you that you are enrolled in this clinical trial.
- If you experience a deterioration in your mental health or make use of these services, we ask that you also inform trial staff.

# APPENDIX E: Dosing day information sheet

Dear XXX,

This is a reminder that your dosing session is [tomorrow/1 week away]. This email contains key information for you to remember and instructions for you to follow in the days leading up to your dose day. The trial staff will have gone over all of this information with you in the preceding sessions but take your time and review the information carefully. Please talk with the study coordinator if you have any questions.

VISIT INFORMATION:

**Visit Date:**XX/XX/XXXX

**Arrival Time:** 9.00am

**Location:** SPW Building, Frederick St, Hawthorn (Swinburne University)

**Coordinating Principal Investigator Contact Information:** Prof Susan Rossell, (03) XXXX XXXX, xxxxxxxxxx

**General Trial staff Contact Information:** (03) XXXX XXXX, psilotrd@swin.edu.au

INSTRUCTIONS BEFORE YOUR DOSE DAY

- **Sleep**: Try to get good quality sleep in the few nights leading up to your dose session.
- **Stress**: Try to minimise stress and have a quiet few days leading up to your session.
- **Alcohol and drugs:** You must refrain from using alcohol or any psychoactive or illicit drug in the 7 days before your dosing session, and for 2 days after your dosing session.
  - *Note, you will be tested for drug use prior to the dose session. If your test is positive, we may have to cancel your dose session.*
- **Medications:**
  - You may take your regular approved medications, these are:

XXX

XXX

XXX

- You should not take any additional prescription or non-prescription medications or herbal supplements, outside of your usual medications, for the week before the dose session (note: over-the-counter painkillers at recommended dosage are fine)
- The following medications must NOT be taken for the week before the dose day:

Long-acting opioid medications (e.g. sustained release oxycodone or morphine)

Antidepressant medications

Rifamycin

Anticonvulsants

Nevirapine

Efavirenz

Paclitaxol

St John's Wort

HIV protease inhibitors

Itraconazole

Ketoconazole

Erythromycin

Clarithromycin

Troleandomycin

Ergot alkaloids

Pimozide

Midazolam

Triazolam

Lovastatin

Simvastatin

Fentanyl

- *Note: if you do take any of these medications in the week before the dose day, we may have to cancel the dose session.*

INSTRUCTIONS FOR THE MORNING OF YOUR DOSE DAY

- **Breakfast**: Please eat a light low-fat breakfast on the morning of the dose session. Foods we recommend include: fruit, toast, yogurt, or low sugar cereals.
- **Caffeine and nicotine**: You should consume your regular amounts of caffeine and/or nicotine before your session.
- **What to wear:** Loose, comfortable clothing.
- **Getting there:** Please arrive on time (9.00am). If possible, your designated ‘support person’ should accompany you to the research site. They should drive you there, or a taxi can be arranged for you by contacting the trial coordinator in advance.

WHAT TO BRING TO YOUR DOSE DAY

- A blanket or pillow if you prefer to use your own.
- Any small personally meaningful items such as photos, images, or objects that you would like to have with you.
- An additional change of clothes and warm clothes.
- A light lunch or snacks. These need to be foods that don’t require reheating or refrigeration and should be light foods, for example a sandwich, crackers, fruit, etc.
- Any regular, approved medications that need to be taken during the day.

WHAT WILL HAPPEN DURING YOUR DOSE DAY

- **Arrival**: Your therapist team will meet you outside the building at 9am and escort you to the therapy room.
- **Before dose:** The therapists will take your blood pressure and heart rate, ask you to provide a urine sample for a drug/pregnancy test and check you haven’t taken any non-allowed medications. The therapists will check that you are ready to proceed with the dose session. You will also complete a brief questionnaire.
- **Dosing**: Once you’re settled and ready (around 10am) you will be given the capsule containing psilocybin with a glass of water.
- **During the session:** You will remain in the therapy room throughout the session; if you need to use the bathroom, one of the therapists will escort you there. At least one therapist will remain with you throughout the session to provide support. Blood pressure and heart rate measurements will be taken regularly.
- **Departing**: When you are ready to leave, the therapists will escort you to back to the foyer of the SPW building, where your support person should meet you. Your support person must escort you home. They can do so in their car, or in a taxi that the trial staff can arrange (if this is required, please contact the Coordinating Principal Investigator). You must not take public transport or walk home.

AFTER YOUR DOSE DAY

- **Support person:** Your support person should stay with you for 24 hours after your dose session.
- **Home environment:** Try to make your home quiet and comfortable for the night of your dose session.
- **Time off work:** If possible, try to take time off work after your dose day, or allow for a restful day.
- **You must not drive a car for 48 hours** after receiving psilocybin.

ON-CALL STUDY PERSONNEL

- Your therapist team will be on-call throughout the night of your dose session and the next day to speak with you should you require urgent support. You can reach them via the study phone number:
  - (03) **XXXX XXXX**
- If there is any medical or psychiatric emergency, please call 000 immediately.

# APPENDIX F: Physical contact protocol

PSILO-TRD Trial: Psilocybin for Treatment Resistant Depression

**PHYSICAL CONTACT PROTOCOL**

*This document is to be read, signed, and returned to the trial coordinator, by all therapists in the PsiloTRD clinical trial. If therapists have any questions or concerns about the contents of this document, they are welcome to discuss them with the Trial Coordinator, Sally Meikle, or the Coordinating Principal Investigator, Prof. Susan Rossell.*

Within the PsiloTRD clinical trial, physical contact between therapists and participants, or ‘therapeutic touch’ is:

- To be used *minimally* – we are not practicing a ‘somatic therapy’, but offering simple touch where supportive or requested, subject to the conditions set out below;
- Limited to dosing sessions, and practice during preparation sessions.
  - Therapeutic touch is not used in non-drug psychotherapy sessions.
  - Therapists should not initiate a hug at the beginning or end of psychotherapy sessions and should use discretion when responding to a participant who has initiated a hug or other touch outside the dosing sessions.
- Not required from therapists - a therapist may not wish to offer therapeutic touch for a variety of reasons. It is not required for therapists to offer therapeutic touch in this trial. Therapists are encouraged to practice within their competency and comfort level/personal boundaries.
- Never sexual or sensual – therapists should consider both their own intentions and the possible perception of touch by the participant.
  - Therapists are not permitted to lie down with or beside participants at any point
  - Therapists are not permitted to provide touch between the waist and knees, or in contact with female participants’ breasts.
- Subject to consent, which can be withdrawn by the participant at any time.
- Only used after therapeutic touch has been explained by therapists, the participant has been given an opportunity to ask questions, the participant’s preferences have been discussed, and methods of verbal and non-verbal requests for touch and declining touch have been discussed and practiced, including:
  - Explain the role of therapeutic touch, give some examples (a gentle and reassuring touch on a shoulder, holding the participant’s hand), why it can be useful, when it might be used, and that it is entirely optional.
    - Be cautious of implicit coercion into consent (i.e., talking up the benefits or necessity of touch may encourage those who do not feel comfortable with it to consent to it in order to fully benefit from the treatment or to please their therapists).
  - Note and explain that therapeutic touch is used minimally or as requested, is never sexual or sensual, and will never be between the waist and knees, or touch female participants’ breasts.
  - Ask how the participant feels about therapeutic touch, if they have questions, what they would find useful/not useful.
  - Explain that therapeutic touch is only done with prior consent during the preparatory sessions, and this consent can be rescinded at any time by asking the therapist to stop or with a hand gesture.
  - Explain that no new type of touch can be requested if this has not been consented to prior to drug ingestion.
  - Note: a participant can consent to any type of permissible therapeutic touch (i.e., they are not required to constrain the type of touch), or can specify limited types of therapeutic touch for therapist-initiated touch alongside all types of permissible therapeutic touch for participant-initiated touch (i.e., there can be a distinction between therapist-initiated and participant-initiated).
  - Explain need to practice, request participant consent, and then establish preferred method of therapeutic touch, and preferred methods to request touch/no touch.
  - Provide participant with the PsiloTRD participant information sheet, which includes an outline of the physical contact protocol.
- Documented in a preparation session. Specifically:
  - Whether the participant consents to any physical contact.
  - Whether the physical contact may be therapist initiated or only participant initiated.
  - The type of touch consented to and any exceptions to this (i.e., hand holding consented to, no touching of knees, etc.).
  - Which therapist(s) form the dyad may provide touch.
  - Which words and gestures may be used by the participant as ‘stop cues’.

Full Name:

Signature:

Date:

# APPENDIX G: Support Person Information

**PsiloTRD Support Person Information**

An Open-label Proof of Concept Study of Psilocybin-Assisted Psychotherapy for Treatment-resistant Depression

This document contains information about your role as a ‘support person’ for a participant in the PsiloTRD trial.

**Trial information**

- Your friend/family member is participating in a clinical trial of an experimental treatment called ‘psilocybin-assisted psychotherapy’. This treatment involves a combination of a drug (psilocybin) and psychotherapy. This drug is predicted to work differently from other drugs used to treat depression. Rather than being taken every day, it is used in combination with psychotherapy on two occasions - called ‘dosing sessions’.
- In this trial, there will be two dosing sessions, 6 weeks apart, with psychotherapy sessions before and after each dosing session. At each dosing session, the participant will be given 25 mg of psilocybin under the supervision of two therapists.  Psilocybin is a psychoactive drug that usually produces substantial changes in perception, feelings, and thinking that will last for several hours. The dosing sessions will last 6-8 hours and will not end until the effects of the psilocybin have subsided.
- Outside of the treatment component of the trial, the participant will be asked to complete several assessments, both in-person and remotely.

**Support Person**

- The experience a participant has during a dosing session can be challenging and intense; the effects of this drug can sometimes be strange or unpleasant. While these effects will subside before the dosing sessions ends, people can sometimes feel unsettled or ‘fragile’ in days following this experience. Therefore, it is important that people are well supported immediately following a dosing session.
- One of the ways we ensure participants are well supported following their dosing session, is by asking them to nominate a ‘support person’. This person is a close friend or family member who the participant feels comfortable with, and who is supportive of their mental health and participation in this clinical trial.

**Role/responsibilities**

As the elected support person for a participant in this clinical trial:

- You must accompany participant home after dosing sessions and stay with them for 24 hours
  - Pick up
    - Dosing sessions will start at 9.00am, the end time will vary from participant to participant. The therapist will call you and let you now what time to come pick up the participant this will likely be some time between 4.00pm-6.00pm. If you are travelling from far away, please let the trial staff know so we can take this into account.
    - The dosing session will occur in the SPW building at Swinburne University of Technology, Hawthorn. For more information about getting there, see the ‘Transport’ section below.
    - When you arrive, please wait in the foyer of the SPW building (enter via the west side of the building, on Frederick St), the therapists will escort the participant from the dosing room down to the foyer to meet you.
  - Going home
    - Preferably, you should drive the participant home after the dosing session. If you do not have a car, you should take a taxi. The participant will receive $200 to cover travel expenses upon completion of the trial. Public transport should not be used to escort the participant home after a dosing session.
    - If possible, you should stay with the participant in their home. It is best for people to be in comfortable, familiar environments following dosing sessions.
- You should support the participant following dosing sessions.
  - This includes:
    - Helping ensure the home environment is quiet and comfortable.
    - Giving them space or being there to talk to if they request it.
  - Please note, if the participant is experiencing distress and requires urgent support, they may call their therapists during the 24 hours following a dosing session using the following phone number: (03) **XXXX XXXX**
  - If there is any medical or psychiatric emergency, please call **000** immediately.
- You may also accompany the participant to the dosing session, and any other psychotherapy session or trial assessment. If you do, you will not be allowed in the room with the participant but you can wait on-site.

**Transport**

- All dosing sessions (as well as all other psychotherapy sessions and in-person trial assessments) are conducted in the Swinburne Place West (SPW) building at Swinburne University of Technology, Hawthorn. See the map below.
  - When you pick-up or drop-off the participant, enter via the west side of the building on Frederick St and meet trial staff in the foyer.
- Parking:
  - Swinburne university has a multistorey paid undercover parking lot located at 15 Wakefield Street. See building marked 19W on the map below. Parking costs $4 for 0-2 hours, $5 for 2-3 hours and $10 for 3+ hours. Payment can be made by cash or credit card.
  - There is also ticket parking available off Wakefield St and Park Street, near Glenferrie Road, and a variety of on-street parking available on the surrounding streets.
- Public transportation:
  - Train: Glenferrie train station (on the Belgrave/Alamein line) is located close to the SPW building.
  - Tram: The route 16 tram will get you to Glenferrie Road, near the SPW building.


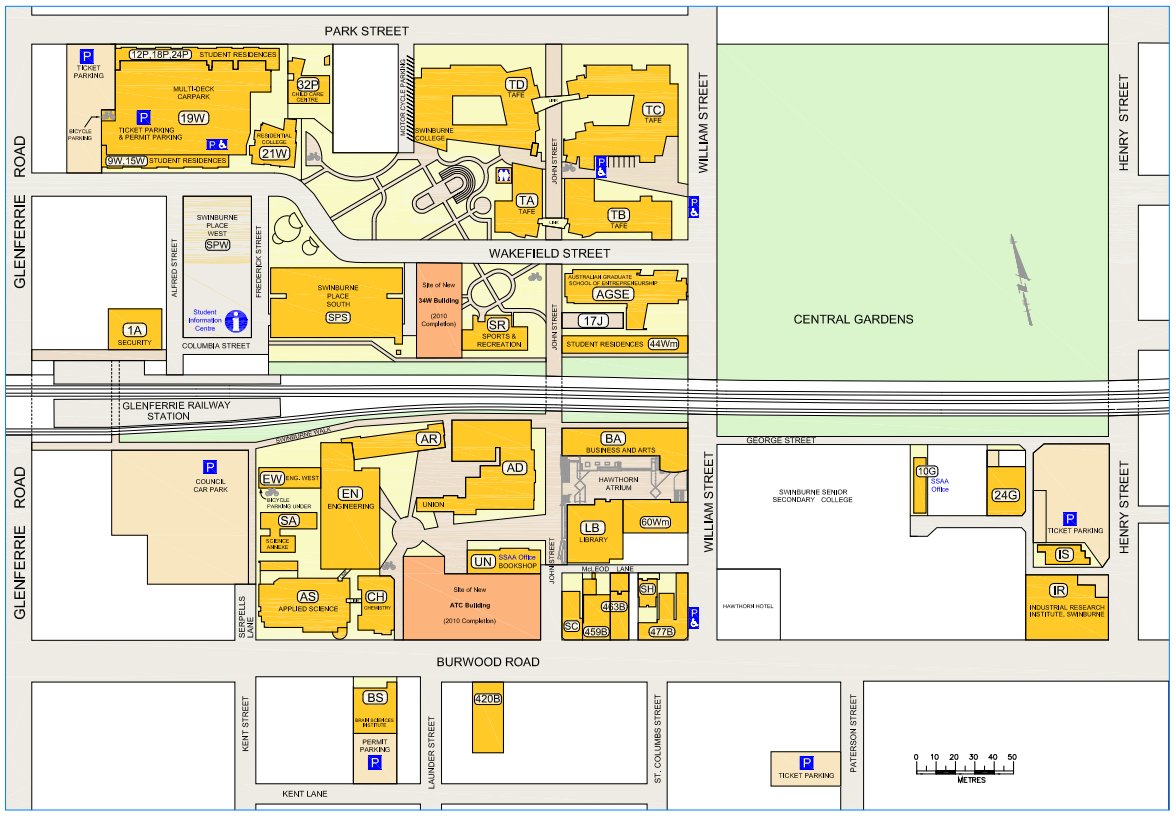


**Who to talk to**

Trial coordinator

The trial coordinator, Sally Meikle, is the primary point of contact for any general questions or concerns about any aspect of the trial.

Email: [psilotrd@swin.edu.au](mailto:psilotrd@swin.edu.au)

Coordinating Principal Investigator

If preferred, the coordinating principal investigator, Professor Susan Rossell, may be contacted.

Email: [XXXXXXXX](mailto:srossell@swin.edu.au)

# APPENDIX H: Support Person PICF

**Participant Information Sheet/Consent Form – Support Person**

**Interventional Study** - *Adult providing own consent*

Swinburne University, Melbourne

| **Title** | An Open-label Proof of Concept Study of Psilocybin-Assisted Psychotherapy for Treatment-resistant Depression |
| --- | --- |
| **Short Title** | PsiloTRD |
| **Protocol Number** | 20201367-3726 |
| **Project Sponsor** | Investigator-Initiated |
| **Coordinating Principal Investigator/ Principal Investigator** | Prof Susan Rossell |
| **Location** | Swinburne University, VIC |

**Part 1 What does my participation involve?**

**1 Introduction**

You are invited to take part in this research project. This is because a friend/family member of yours is participating in our research trial of a new treatment for depression and has nominated you as their ‘support person’.

The new treatment that your friend/family member will receive in this research trial involves multiple sessions of a specialised form of psychotherapy and one or two doses of a drug called psilocybin. This treatment approach is called ‘psilocybin-assisted psychotherapy’.

It is important that your friend/family member is supported throughout their participation in this study, particularly after their psilocybin sessions. You have been nominated by your friend/family member to support them after these sessions by accompanying them home afterwards and staying with them for at least 24 hours. If you have any questions or concerns about your role in supporting your friend/family member, please speak to our research team.

In addition, and separately to your role in supporting your friend/family member, you are invited to participate in this research project. Your participation will only involve completing a survey. This survey will ask you about your friend/family member’s condition, from your perspective. You will be asked to complete the same survey before and after your friend/family member undergoes the psilocybin-assisted psychotherapy.

This Participant Information Sheet/Consent Form tells you about the research project. It explains what your participation involves. Knowing what is involved will help you decide if you want to take part in the research.

Please read this information carefully. Ask questions about anything you don’t understand or want to know more about. Before deciding whether or not to take part, you might want to talk about it with a relative, friend, or your doctor.

Participation in this research is voluntary. If you don’t wish to take part, you don’t have to.

If you decide you want to take part in the research project, you will be asked to sign the consent section. By signing it you are telling us that you:

• Understand what you have read

• Consent to take part in the research project

• Consent to complete the surveys described

• Consent to the use of the information you provide as described

You will be given a copy of this Participant Information and Consent Form to keep.

**2 What is the purpose of this research?**

Up to one-third of adults experiencing depression have symptoms that do not respond to treatment with available antidepressant medications. These patients may be referred to as having ‘treatment-resistant’ depression (MDD-TR). Psilocybin-assisted psychotherapy is an experimental intervention that could improve the symptoms of depression. This study will determine whether this new treatment is safe and effective in treating MDD-TR. In addition, the study will take a number of measures to see how this treatment works.

Psilocybin, the drug used in psilocybin-assisted psychotherapy, is not approved for MDD-TR, and therefore it is an experimental treatment.

The study Chief Investigator is Professor Susan Rossell. This research has been funded by the Barbara Dicker Brain Sciences Foundation and Usona Institute.

Data collected in this trial will contribute to the PhD projects of Ms Sally Meikle and Ms Lauren Johansen.

**3 What does participation in this research involve?**

Your participation involves completing an online survey at three time points: at an initial baseline time, then at 12 and 29 weeks after that. This survey will ask you for your perspective on your friend/family member’s condition. It will take approximately 20 minutes to complete. Your responses will not be disclosed to your friend/family member.

If you agree to participate in this study, you will be asked to sign this consent form before you complete any study surveys. This research is being conducted at Swinburne University of Technology, Hawthorn. However, you will complete your surveys online and do not need to come to the university to complete any assessments.

There are no costs associated with participating in this research project. You will not receive any reimbursements.

**4 Other relevant information about the research project**

All nominated ‘support people’ will be invited to participate in this part of the study.

All participants will be recruited from Melbourne.

**5 Do I have to take part in this research project?**

Participation in any research project is voluntary. If you do not wish to take part, you do not have to. If you decide to take part and later change your mind, you are free to withdraw from the study at any stage.

Your decision whether to take part or not to take part, or to take part and then withdraw, will not affect your friend/family member who is undergoing the treatment part of this research study. If you chose not to take part in the research or chose to withdraw at any time, you can still be the designated support person for your friend/family member. Your role as the support person, and your participation in research by completing a survey, are separate.

**6 What are the alternatives to participation?**

You do not have to take part in this research project. Other options are available; these include remaining in your role as the support person for your friend/family member.

**7 What are the possible benefits of taking part?**

There will be no personal benefits to you in participating in this research; however, possible wider benefits include an improved understanding of treatments for depression.

**8 What are the possible risks and disadvantages of taking part?**

The possible risks or disadvantages of taking part include psychological distress or discomfort while answering questions about your friend/family member’s condition.

**9 What if I withdraw from this research project?**

If you decide to withdraw from the project, please notify a member of the research team.

If you do withdraw your consent during the research project, the study researcher and relevant study staff will not collect additional information from you, although information already collected will be retained to ensure that the results of the research project can be measured properly and to comply with law. You should be aware that data collected up to the time you withdraw will form part of the research project results. If you do not want them to do this, you must tell them before you join the research project.

**10 Could this research project be stopped unexpectedly?**

This research project may be stopped unexpectedly for a variety of reasons. These may include reasons such as:

• Unacceptable side effects

• The drug/treatment being shown not to be effective

**11 What happens when the research project ends?**

A summary of the general findings of this research project will be made available to all participants via post or email at the study conclusion, if they have indicated consent to receive such further communication.

**Part 2 How is the research project being conducted?**

**12 What will happen to information about me and my friend/family member?**

By signing the consent form you consent to the study researcher and relevant research staff collecting and using the information you provide for the research project. Any information obtained in connection with this research project that can identify you or your friend/family member will remain confidential. The information you provide will not be disclosed to your friend/family member.

In the handling of data, all references to personal information will be removed and replaced by a code, so that participants will not be able to be individually identified. All data will be stored securely, under lock-and-key, or via password protection, at the research venue. Access to the data will only be available to the principal researchers and the supervised trial coordinators/research assistants responsible for this study.

Your information will only be used for the purpose of this research project and it will only be disclosed with your permission, except as required by law.

It is anticipated that the results of this research project will be published and/or presented in a variety of forums. In any publication and/or presentation, information will be provided in such a way that you cannot be identified, except with your permission. All participants will remain anonymous.

In accordance with relevant Australian and/or State privacy and other relevant laws, you have the right to request access to your information collected and stored by the research team. You also have the right to request that any information with which you disagree be corrected. Please contact the study team member named at the end of this document if you would like to access your information.

Any information obtained for the purpose of this research project and for the future research described in this section that can identify you will be treated as confidential and securely stored. It will be disclosed only with your permission, or as required by law.

All of the data collected as part of this study will be stored indefinitely in a databank. By signing the consent form, you are consenting to participate in this study and for your data to be used in a de-identified format in future research. If you do not wish for your data to be stored indefinitely in the databank, you will need to indicate this by ticking the appropriate box in the consent form.

**13 Complaints and compensation**

If you suffer any injuries or complications as a result of this research project, you should contact the study team as soon as possible and you will be assisted with arranging appropriate medical treatment. If you are eligible for Medicare, you can receive any medical treatment required to treat the injury or complication, free of charge, as a public patient in any Australian public hospital.

In the event of loss or injury, the parties involved in this research project have agreed to assist you with arranging appropriate medical treatment.

**14 Who is organising and funding the research?**

This research project is being conducted by a team of clinical scientists and doctors from Melbourne, Australia and London, United Kingdom. The Chief Principal Investigator is Prof. Susan Rossell.

This research project is being funded by Barbara Dicker Brain Sciences Foundation and Usona Institute.

**15 Who has reviewed the research project?**

All research in Australia involving humans is reviewed by an independent group of people called a Human Research Ethics Committee (HREC). The ethical aspects of this research project have been approved by the HREC of Swinburne University, Melbourne.

This project will be carried out according to the *National Statement on Ethical Conduct in Human Research (2007)*. This statement has been developed to protect the interests of people who agree to participate in human research studies.

**16 Further information and who to contact**

The person you may need to contact will depend on the nature of your query. If you want any further information concerning this project you can contact any of the following people:

**General research staff**

| Telephone | (03) XXX XXX |
| --- | --- |
| Email | psilotrd@swin.edu.au |

**Coordinating Principal Investigator**

| Name | Prof Susan Rossell |
| --- | --- |
| Telephone | (03) XXXX XXXX |
| Email | xxxxxxxxxxxx |

**Trial Student**

| Name | Sally Meikle |
| --- | --- |
| Telephone | (+61) XXXX XXXX |
| Email | xxxxxxxxxxxxxx |

**Clinical contact person**

| Name | Peter Bosanac |
| --- | --- |
| Telephone | (03) XXXX XXXX |
| Email | xxxxxxxxxxxx |

If you have any complaints about any aspect of the project, the way it is being conducted or any questions about being a research participant in general, then you may contact:

**Reviewing HREC approving this research** **and HREC Executive Officer details**

| Position | HREC Executive Officer |
| --- | --- |
| Telephone | +61 3 9214 3845 or +61 3 9214 8145 |
| Email | [resethics@swinburne.edu.au](mailto:resethics@swinburne.edu.au) |

**Consent Form -** *Adult providing own consent*

| **Title** | An Open-label Proof of Concept Study of Psilocybin-Assisted Psychotherapy for Treatment-resistant Depression |
| --- | --- |
| **Short Title** | PsiloTRD |
| **Protocol Number** | 20201367-3726 |
| **Project Sponsor** | Investigator-initiated |
| **Coordinating Principal Investigator/**  **Principal Investigator** | Prof Susan Rossell |
| **Location** | Swinburne University, VIC |

**Declaration by Participant**

I have read the Participant Information Sheet or someone has read it to me in a language that I understand.

I understand the purposes, procedures and risks of the research described in the project.

I have had an opportunity to ask questions and I am satisfied with the answers I have received.

I freely agree to participate in this research project as described and understand that I am free to withdraw at any time during the study without affecting my future health care or the health care of my friend/family member.

I understand that I will be given a signed copy of this document to keep.

Please tick this box if you want your data used ONLY for the purpose of this project,

And DO NOT want your data stored indefinitely in a databank.

|  | | | | | | |
| --- | --- | --- | --- | --- | --- | --- |
|  | Name of Participant (please print) | |  |  |  |  |
|  | | | | | | |
|  | Signature |  | | Date |  |  |
|  | | | | | | |

**Form for Withdrawal of Participation -** *Adult providing own consent*

| **Title** | An Open-label Proof of Concept Study of Psilocybin-Assisted Psychotherapy for Treatment-resistant Depression |
| --- | --- |
| **Short Title** | PsiloTRD |
| **Protocol Number** | 20201367-3726 |
| **Project Sponsor** | Investigator-initiated |
| **Coordinating Principal Investigator/**  **Principal Investigator** | Prof Susan Rossell |
| **Location** | Swinburne University, VIC |

**Declaration by Participant**

I wish to withdraw from participation in the above research project and understand that such withdrawal will not affect my routine treatment, my relationship with those treating me or my relationship with Swinburne University

|  | | | | | | |
| --- | --- | --- | --- | --- | --- | --- |
|  | Name of Participant (please print) | |  |  |  |  |
|  | | | | | | |
|  | Signature |  | | Date |  |  |
|  | | | | | | |

|  |
| --- |

**Declaration by Study Doctor/Senior Researcher^†^**

I have given a verbal explanation of the implications of withdrawal from the research project and I believe that the participant has understood that explanation.

|  | | | | | | |
| --- | --- | --- | --- | --- | --- | --- |
|  | Name of Study Doctor/  Senior Researcher^†^ (please print) | |  | | |  |
|  | | | | | |  |
|  | Signature |  | | Date |  |  |
|  | | | | | | |

^†^ A senior member of the research team must provide the explanation of and information concerning withdrawal from the research project.

Note: All parties signing the consent section must date their own signature.
