# Supplementary material for: Psilocybin with psychotherapeutic support for treatment-resistant depression: a pilot clinical trial
Source: Ther Adv Psychopharmacol. 2025 Oct 2;15:20451253251377187. doi: 10.1177/20451253251377187 (PMC12491823; doi:10.1177/20451253251377187)
Supplement: sj-docx-2-tpp-10.1177_20451253251377187 – Supplemental material for Psilocybin with psychotherapeutic support for treatment-resistant depression: a pilot clinical trial [file sj-docx-2-tpp-10.1177_20451253251377187.docx]

**Supplementary Results**

Table of Contents

[Data Handling 3](#_Toc202876013)

[Participants 4](#_Toc202876014)

[Table S1: Reasons for ineligibility at each screening stage 4](#_Toc202876015)

[Table S2. Methods through which participants found this trial 6](#_Toc202876016)

[Table S3: Exact paired permutation tests for secondary mental health and wellbeing outcomes 6](#_Toc202876017)

[Primary and secondary health and wellbeing outcomes 7](#_Toc202876018)

[Table S4: Change in primary and secondary health and wellbeing outcomes by treatment trajectory 7](#_Toc202876019)

[Table S5: Persisting Effects Questionnaire (PEQ) scores by treatment trajectory 8](#_Toc202876020)

[Exploring indicators of clinical improvement 9](#_Toc202876021)

[Expectations 9](#_Toc202876022)

[Full multinomial logistic regression results 10](#_Toc202876023)

[Table S6a: Multinomial Logistic Regression Models using the predictor Hope of Improvement (PATHEV - baseline) 10](#_Toc202876024)

[Table S6b: Multinomial Logistic Regression Models using the predictor Fear of Change (PATHEV - baseline) 10](#_Toc202876025)

[Table S6c: Multinomial Logistic Regression Models using the predictor Suitability (PATHEV - baseline) 10](#_Toc202876026)

[Table S6d: Multinomial Logistic Regression Models using the predictor Mindset 11](#_Toc202876027)

[Table S6e: Multinomial Logistic Regression Models using the predictor Unity of Experience (11D-ASC) 11](#_Toc202876028)

[Table S6f: Multinomial Logistic Regression Models using the predictor Spiritual Experience (11D-ASC) 11](#_Toc202876029)

[Table S6g: Multinomial Logistic Regression Models using the predictor Blissful State (11D-ASC) 12](#_Toc202876030)

[Table S6h: Multinomial Logistic Regression Models using the predictor Insightfulness (11D-ASC) 12](#_Toc202876031)

[Table S6i: Multinomial Logistic Regression Models using the predictor Disembodiment (11D-ASC) 12](#_Toc202876032)

[Table S6j: Multinomial Logistic Regression Models using the predictor Impaired Control and Cognition (11D-ASC) 13](#_Toc202876033)

[Table S6k: Multinomial Logistic Regression Models using the predictor Anxiety (11D-ASC) 13](#_Toc202876034)

[Table S6l: Multinomial Logistic Regression Models using the predictor Complex Imagery (11D-ASC) 13](#_Toc202876035)

[Table S6m: Multinomial Logistic Regression Models using the predictor Elemental Imagery (11D-ASC) 14](#_Toc202876036)

[Table S6n: Multinomial Logistic Regression Models using the predictor Audio-Visual Synaesthesia (11D-ASC) 14](#_Toc202876037)

[Table S6o: Multinomial Logistic Regression Models using the predictor Changed Meaning of Percepts (11D-ASC) 14](#_Toc202876038)

[Table S6p: Multinomial Logistic Regression Models using the predictor PIQ 15](#_Toc202876039)

[Table S6q: Multinomial Logistic Regression Models using the predictor EBI 15](#_Toc202876040)

[Feasibility and participant safety and wellbeing 15](#_Toc202876041)

[Table S7: Adverse events during screening 16](#_Toc202876042)

[Table S8: Adverse events by participant reported after enrolment 17](#_Toc202876043)

[Protocol deviations 20](#_Toc202876044)

[REFERENCES 21](#_Toc202876045)

# Data Handling

For 11D-ASC subscales, PIQ, EBI, and Mindset, the average of scores for dose 1 and dose 2 were used in the multinomial logistic regressions, while for the PATHEV subscales, baseline scores were used.

All data analysed here were checked for outliers using z-score calculations with no outliers found. Mindset questionnaire data was missing for 1 participant at 1 dosing session. For analyses involving Mindset data, the single dosing session score was used in place of the average score for this participant.

The following packages in R were used: coin for exact paired permutation tests (Hothorn et al., 2006); effsize for hedges’ g (Torchiano, 2020); nnet for multinomial logistic regressions (Venables, 2002); pscl for model fit statistics (Jackman, 2010). For Hedges’ g, the Cohen’s d function was used, specifying for paired comparisons and the Hedges’ g correction for small sample sizes.

# Participants

## Table S1: Reasons for ineligibility at each screening stage

| **Exclusion Criteria:** | **Ineligible at online survey  (*n* = 98)** | **Ineligible at zoom interview  (*n* = 38) *** | **Ineligible at healthcare consultation  (*n* = 3)** | **Ineligible after enrolment  (*n* = 1)** | **Total**  **(*n* = 133)** |
| --- | --- | --- | --- | --- | --- |
| Over 65 years old | 5 | NA | - | - | **5** |
| Unable to commit to trial visit and assessment requirements | 4 | 0 | - | 0 | **4** |
| No diagnosis of a depressive disorder | 8 | 0 | - | - | **8** |
| Not TRD | 23 | 9 | - | - | **32** |
| Not currently under the care of a psychiatrist, psychologist, GP or other healthcare provider for depression | 13 | 1 | 0 | - | **14** |
| Ever diagnosed with Bipolar I or II disorder | 9 | 0 | 0 | 0 | **9** |
| First degree relative diagnosed with schizophrenia or psychotic disorder (not caused by medication or medical condition) | 4 | 1 | - | 0 | **5** |
| First degree relative diagnosed with bipolar I or II disorder | 8 | 1 | - | 0 | **9** |
| Diagnosed with alcohol or drug dependence (excluding caffeine and nicotine) within the past 5 years | 11 | 2 | 0 | 0 | **13** |
| Current diagnosis of a dissociative disorder | 2 | 0 | 0 | 0 | **2** |
| Current diagnosis of anorexia nervosa or bulimia nervosa | 2 | 1 | 1 | 0 | **4** |
| Ever been diagnosed with epilepsy or seizures | 4 | 0 | 0 | - | **4** |
| Current hypothyroidism | 7 | 1 | 0 | 0 | **8** |
| Taken a macrodose of a psychedelic in the last 12 months | 25 | 2 | - | - | **27** |
| Taken a microdose of a psychedelic within the last month | 6 | 0 | - | - | **6** |
| Not currently living in Victoria | 10 | - | - | - | **10** |
| MADRS < 30 | NA | 20 | - | - | **20** |
| Any psychiatric conditions (including personality disorders) or suicidal ideation judged to be incompatible with establishment of rapport or safe exposure to psilocybin | NA | 1 | 0 | 0 | **1** |
| Any current personal of situational factors that, in the opinion of the investigators or study doctors, might interfere with participation | NA | 5 | 0 | 0 | **5** |
| Presence of factors leading to a ‘complex case’ of depression (for example: childhood trauma, multiple or complex psychiatric or medical comorbidities, or comorbidities where depression appears to be secondary) | NA | 13 | 0 | 1 | **14** |
| Not vaccinated against COVID-19 | NA | 2 | - | - | **2** |
| No longer wishes to participate | NA | 0 | 1 | 0 | **1** |
| Unable to safely taper off antidepressant medication(s) | NA | NA | 1 | - | **1** |

Only the primary reason(s) for participant exclusion included here, interviews may be terminated once exclusionary criteria are discovered and thus not all criteria are assessed. No participants determined ineligible at the medical exam (n=12). Only criteria for which at least 1 participant was excluded due to are included here.

## Table S2. Methods through which participants found this trial

|  | Complete survey responses  (*n* = 206) | Enrolled participants  (*n* = 8) |
| --- | --- | --- |
| Media article | 14 (6.80%) | 0 |
| Australianclinicaltrials.gov.au | 101 (49.03%) | 6 (75%) |
| Friend or family member | 17 (8.25%) | 0 |
| Healthcare professional | 39 (18.93%) | 2 (25%) |
| Other sources | 35 (16.99) | 0 |

## Table S3: Exact paired permutation tests for secondary mental health and wellbeing outcomes

| **Variable** | **Mean difference** | **95% CI**  **Raw scores** | **p-value** | **Hedges’ g** | **95% CI Hedges’ g** |
| --- | --- | --- | --- | --- | --- |
| **Primary outcome** |  |  |  |  |  |
| QIDS (to primary endpoint) | -7.14 | [-10.14, -4.14] | 0.02 | -1.27 | [-2.40, -0.37] |
| QIDS (to long-term follow-up) | -7.14 | [-10.86, -3.00] | 0.02 | -1.16 | [-2.77, -0.46] |
| **Secondary outcomes (to primary endpoint)** | | | | | |
| WHO QoL total | 28.57 | [14.29, 42.86] | 0.03 | 0.83 | [0.21, 2.09] |
| WHO QoL physical | 15.31 | [3.58, 27.04] | 0.06 | 0.68 | [0.22, 1.64] |
| WHO QoL psychological | 20.83 | [8.33, 33.93] | 0.05 | 0.86 | [0.37, 1.65] |
| WHO QoL social | 13.09 | [3.57, 22.62] | 0.13 | 0.58 | [0.11, 1.23] |
| WHO QoL environment | 12.05 | [4.91, 18.75] | 0.05 | 0.69 | [0.27, 1.79] |
| GAD-7 | -3.00 | [-6.71, 0.57] | 0.25 | -0.41 | [-2.04, 0.06] |
| RSES total | 21.71 | [5.14, 37.00] | 0.08 | 0.73 | [0.06, 1.47] |
| BEAQ | -18.43 | [-28.57, -8.86] | 0.02 | -1.32 | [-2.62, -0.66] |
| WCS | 17.61 | [4.08, 30.72] | 0.11 | 0.66 | [0.19, 1.45] |

# Primary and secondary health and wellbeing outcomes

## Table S4: Change in primary and secondary health and wellbeing outcomes by treatment trajectory

|  | **Sustained response**  **(n = 2)** | **Relapsing  (n = 3)** | **Non-response  (n = 2)** | **Total  (n = 7)** |
| --- | --- | --- | --- | --- |
| **Primary outcome** |  |  |  |  |
| QIDS (to primary endpoint) | -11.50 | -7.67 | -2.00 | -7.14 |
| QIDS (to long-term follow-up) | -12.50 | -3.33 | -7.50 | -7.14 |
| **Secondary outcomes (to primary endpoint)** | | | | |
| WHO QoL total | 50.00 | 25.00 | 12.50 | 28.57 |
| WHO QoL physical | 30.36 | 11.91 | 5.36 | 15.31 |
| WHO QoL psychological | 39.59 | 19.45 | 4.17 | 20.83 |
| WHO QoL social | 16.67 | 16.67 | 4.17 | 13.09 |
| WHO QoL environment | 12.50 | 16.67 | 4.69 | 12.05 |
| GAD-7 | -6.50 | -4.33 | 2.50 | -3.00 |
| RSES total | 39.50 | 28.33 | -6.00 | 21.71 |
| BEAQ | -35.00 | -9.00 | -16.00 | -18.43 |
| WCS | 39.62 | 10.28 | 6.59 | 17.61 |

Score ranges: QIDS 0-27; all WHO QoL 0-100; GAD-7 0-21; RSES total 35-175; BEAQ 15-90; WCS 0-100.

## Table S5: Persisting Effects Questionnaire (PEQ) scores by treatment trajectory

|  | **Sustained response**  **(n = 2)** | **Relapsing  (n = 3)** | **Non-response  (n = 2)** | **Total  (n = 7)** |
| --- | --- | --- | --- | --- |
| Positive Attitudes about Life or Self | 75.88 | 31.76 | 20.59 | 42.75 |
| Negative Attitudes about Life or Self | 0.00 | 4.71 | 16.47 | 7.06 |
| Positive Mood Changes | 80.00 | 26.67 | 3.33 | 36.67 |
| Negative Mood Changes | 0.00 | 16.67 | 25.00 | 13.89 |
| Altruistic/Positive Social Effects | 65.00 | 8.33 | 21.25 | 31.53 |
| Antisocial/Negative Social Effects | 6.25 | 10.00 | 0.00 | 5.42 |
| Positive Behavioural Changes | 90.00 | 33.33 | 30.00 | 51.11 |
| Negative Behavioural Changes | 0.00 | 6.67 | 10.00 | 5.56 |

PEQ scores expressed as a percentage of total possible score.


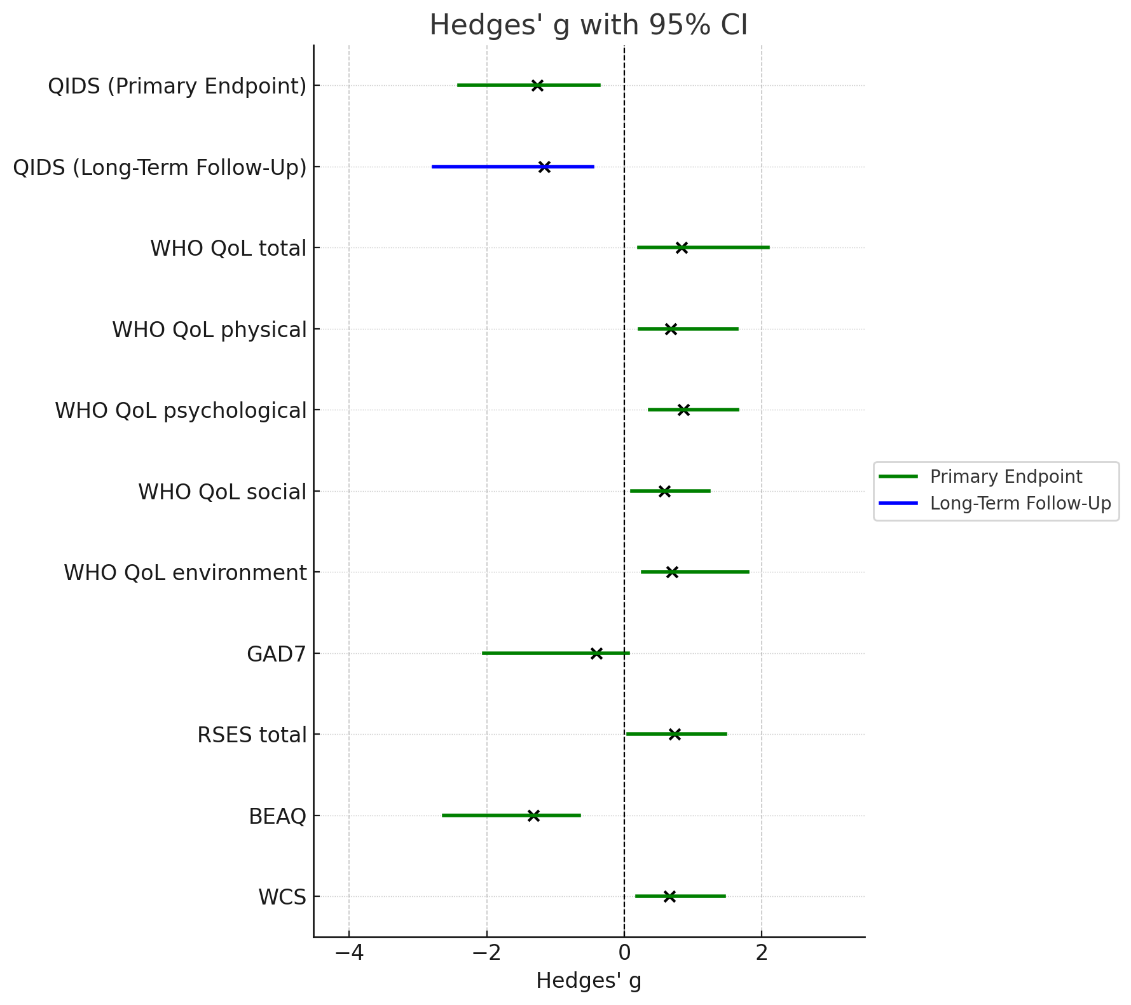


Figure S1: Hedge’s g and 95%CI for secondary mental health and wellbeing outcomes

# Exploring indicators of clinical improvement

## Expectations

A paired t-test revealed treatment expectations (PATHEV) did not change significantly between baseline and 1-day pre-dose 1, after the 3 preparatory psychotherapy sessions (confidence: t(6) = 0.55, p = .60, 95% CI [−1.49,2.34]; fear of change: t(6) = −1.00, p = .36, 95% CI [−0.98,0.41]; suitability: t(6) = 0.81, p = .45, 95% CI [−1.73,3.44]).

## Full multinomial logistic regression results

Bonferroni’s correction for multiple comparisons was used to adjust the alpha level for the 17 regression models. P-values in bold where p < 0.0029.

### Table S6a: Multinomial Logistic Regression Models using the predictor Hope of Improvement (PATHEV - baseline)

| **Model** | **Reference Category** | **Outcome Category** | **Coefficient (B)** | **Standard Error (SE)** | **z-value** | **p-value** | **95% CI** | **Model Fit** | |
| --- | --- | --- | --- | --- | --- | --- | --- | --- | --- |
|  |  |  |  |  |  |  |  | **Log-Likelihood** | **McFadden's R²** |
| **1** | **Sustained** | Relapsing | -0.42 | 0.53 | -0.80 | 0.4261 | [-1.45, 0.61] | **14.33** | **0.05** |
|  |  | Non-response | -0.14 | 0.54 | -0.27 | 0.7906 | [-1.21, 0.92] |  |  |
| **2** | **Relapsing** | Sustained | 0.42 | 0.53 | 0.79 | 0.4267 | [-0.61, 1.45] | **14.33** | **0.05** |
|  |  | Non-response | 0.27 | 0.50 | 0.55 | 0.5803 | [-0.70, 1.24] |  |  |
| **3** | **Non-response** | Sustained | 0.14 | 0.54 | 0.27 | 0.7902 | [-0.92, 1.21] | **14.33** | **0.05** |
|  |  | Relapsing | -0.27 | 0.50 | -0.55 | 0.5797 | [-1.25, 0.70] |  |  |

### Table S6b: Multinomial Logistic Regression Models using the predictor Fear of Change (PATHEV - baseline)

| **Model** | **Reference Category** | **Outcome Category** | **Coefficient (B)** | **Standard Error (SE)** | **z-value** | **p-value** | **95% CI** | **Model Fit** | |
| --- | --- | --- | --- | --- | --- | --- | --- | --- | --- |
|  |  |  |  |  |  |  |  | **Log-Likelihood** | **McFadden's R²** |
| **1** | **Sustained** | Relapsing | -0.19 | 0.41 | -0.47 | 0.6401 | [-0.98, 0.61] | **10.79** | **0.29** |
|  |  | Non-response | 0.74 | 0.75 | 0.99 | 0.3211 | [-0.72, 2.21] |  |  |
| **2** | **Relapsing** | Sustained | 0.19 | 0.41 | 0.47 | 0.6407 | [-0.61, 0.98] | **10.79** | **0.29** |
|  |  | Non-response | 0.93 | 0.78 | 1.19 | 0.2343 | [-0.60, 2.47] |  |  |
| **3** | **Non-response** | Sustained | -0.74 | 0.75 | -0.99 | 0.3212 | [-2.21, 0.73] | **10.79** | **0.29** |
|  |  | Relapsing | -0.93 | 0.78 | -1.19 | 0.2343 | [-2.47, 0.60] |  |  |

### Table S6c: Multinomial Logistic Regression Models using the predictor Suitability (PATHEV - baseline)

| **Model** | **Reference Category** | **Outcome Category** | **Coefficient (B)** | **Standard Error (SE)** | **z-value** | **p-value** | **95% CI** | **Model Fit** | |
| --- | --- | --- | --- | --- | --- | --- | --- | --- | --- |
|  |  |  |  |  |  |  |  | **Log-Likelihood** | **McFadden's R²** |
| **1** | **Sustained** | Relapsing | 0.40 | 0.57 | 0.70 | 0.4856 | [-0.72, 1.51] | **14.46** | **0.04** |
|  |  | Non-response | 0.30 | 0.60 | 0.50 | 0.6171 | [-0.87, 1.47] |  |  |
| **2** | **Relapsing** | Sustained | -0.40 | 0.57 | -0.70 | 0.4853 | [-1.51, 0.72] | **14.46** | **0.04** |
|  |  | Non-response | -0.09 | 0.41 | -0.23 | 0.8150 | [-0.89, 0.70] |  |  |
| **3** | **Non-response** | Sustained | -0.30 | 0.60 | -0.50 | 0.6160 | [-1.48, 0.87] | **14.46** | **0.04** |
|  |  | Relapsing | 0.10 | 0.41 | 0.24 | 0.8129 | [-0.70, 0.89] |  |  |

### Table S6d: Multinomial Logistic Regression Models using the predictor Mindset

| **Model** | **Reference Category** | **Outcome Category** | **Coefficient (B)** | **Standard Error (SE)** | **z-value** | **p-value** | **95% CI** | **Model Fit** | |
| --- | --- | --- | --- | --- | --- | --- | --- | --- | --- |
|  |  |  |  |  |  |  |  | **Log-Likelihood** | **McFadden's R²** |
| **1** | **Sustained** | Relapsing | 0.21 | 0.30 | 0.71 | 0.4798 | [-0.38, 0.81] | **6.22** | **0.58** |
|  |  | Non-response | -2.05 | 0.09 | -23.64 | **<0.0001** | [-2.22, -1.88] |  |  |
| **2** | **Relapsing** | Sustained | -0.17 | 0.29 | -0.58 | 0.5588 | [-0.73, -.39] | **6.38** | **0.58** |
|  |  | Non-response | -1.63 | 0.06 | -29.03 | **<0.0001** | [-1.75, -1.52] |  |  |
| **3** | **Non-response** | Sustained | 0.79 | 0.16 | 4.99 | **<0.0001** | [0.48, 1.09] | **6.93** | **0.54** |
|  |  | Relapsing | 1.01 | 0.14 | 7.32 | **<0.0001** | [0.74, 1.28] |  |  |

### Table S6e: Multinomial Logistic Regression Models using the predictor Unity of Experience (11D-ASC)

| **Model** | **Reference Category** | **Outcome Category** | **Coefficient (B)** | **Standard Error (SE)** | **z-value** | **p-value** | **95% CI** | **Model Fit** | |
| --- | --- | --- | --- | --- | --- | --- | --- | --- | --- |
|  |  |  |  |  |  |  |  | **Log-Likelihood** | **McFadden's R²** |
| **1** | **Sustained** | Relapsing | -0.42 | 0.45 | -0.94 | 0.3496 | [-1.29, 0.46] | **4.46** | **0.70** |
|  |  | Non-response | -0.50 | 0.45 | -1.11 | 0.2691 | [-1.39, 0.39] |  |  |
| **2** | **Relapsing** | Sustained | 0.56 | 0.74 | 0.75 | 0.4505 | [-0.89, 2.00] | **4.33** | **0.71** |
|  |  | Non-response | -0.08 | 0.08 | -1.08 | 0.2821 | [-0.23, 0.07] |  |  |
| **3** | **Non-response** | Sustained | 0.51 | 0.46 | 1.10 | 0.2719 | [-0.40, 1.41] | **4.45** | **0.71** |
|  |  | Relapsing | 0.08 | 0.08 | 1.07 | 0.2856 | [-0.07, 0.24] |  |  |

### Table S6f: Multinomial Logistic Regression Models using the predictor Spiritual Experience (11D-ASC)

| **Model** | **Reference Category** | **Outcome Category** | **Coefficient (B)** | **Standard Error (SE)** | **z-value** | **p-value** | **95% CI** | **Model Fit** | |
| --- | --- | --- | --- | --- | --- | --- | --- | --- | --- |
|  |  |  |  |  |  |  |  | **Log-Likelihood** | **McFadden's R²** |
| **1** | **Sustained** | Relapsing | -0.81 | 0.18 | -4.58 | **<0.0001** | [-1.16, -0.46] | **5.94** | **0.61** |
|  |  | Non-response | -0.85 | 0.18 | -4.72 | **<0.0001** | [-1.20, -0.50] |  |  |
| **2** | **Relapsing** | Sustained | 0.52 | 0.66 | 0.79 | 0.4283 | [-0.77, 1.81] | **6.03** | **0.60** |
|  |  | Non-response | -0.04 | 0.05 | -0.80 | 0.4230 | [-0.13, 0.06] |  |  |
| **3** | **Non-response** | Sustained | 0.55 | 0.64 | 0.86 | 0.3880 | [-0.70, 1.80] | **6.03** | **0.60** |
|  |  | Relapsing | 0.04 | 0.05 | 0.81 | 0.4175 | [-0.06, 0.14] |  |  |

### Table S6g: Multinomial Logistic Regression Models using the predictor Blissful State (11D-ASC)

| **Model** | **Reference Category** | **Outcome Category** | **Coefficient (B)** | **Standard Error (SE)** | **z-value** | **p-value** | **95% CI** | **Model Fit** | |
| --- | --- | --- | --- | --- | --- | --- | --- | --- | --- |
|  |  |  |  |  |  |  |  | **Log-Likelihood** | **McFadden's R²** |
| **1** | **Sustained** | Relapsing | -0.48 | 0.84 | -0.57 | 0.5684 | [-2.14, 1.17] | **0.02** | **1.00** |
|  |  | Non-response | -1.16 | 4.27 | -0.27 | 0.7856 | [-9.53, 7.21] |  |  |
| **2** | **Relapsing** | Sustained | 0.54 | 1.20 | 0.45 | 0.6534 | [-1.82, 2.90] | **0.01** | **1.00** |
|  |  | Non-response | -0.81 | 10.74 | -0.08 | 0.9397 | [-21.86, 20.24] |  |  |
| **3** | **Non-response** | Sustained | 1.12 | 2.20 | 0.51 | 0.6125 | [-3.20, 5.43] | **0.02** | **1.00** |
|  |  | Relapsing | 0.59 | 1.84 | 0.32 | 0.7463 | [-3.01, 4.19] |  |  |

### Table S6h: Multinomial Logistic Regression Models using the predictor Insightfulness (11D-ASC)

| **Model** | **Reference Category** | **Outcome Category** | **Coefficient (B)** | **Standard Error (SE)** | **z-value** | **p-value** | **95% CI** | **Model Fit** | |
| --- | --- | --- | --- | --- | --- | --- | --- | --- | --- |
|  |  |  |  |  |  |  |  | **Log-Likelihood** | **McFadden's R²** |
| **1** | **Sustained** | Relapsing | -0.48 | 0.55 | -0.88 | 0.3793 | [-1.56, 0.60] | **5.35** | **0.65** |
|  |  | Non-response | -0.53 | 0.55 | -0.96 | 0.3374 | [-1.61, 0.55] |  |  |
| **2** | **Relapsing** | Sustained | 0.46 | 0.49 | 0.93 | 0.3521 | [-0.50, 1.42] | **5.37** | **0.64** |
|  |  | Non-response | -0.04 | 0.04 | -1.03 | 0.3022 | [-0.13, 0.04] |  |  |
| **3** | **Non-response** | Sustained | 0.57 | 0.66 | 0.86 | 0.3882 | [-0.72, 1.86] | **5.32** | **0.65** |
|  |  | Relapsing | 0.04 | 0.04 | 1.05 | 0.2956 | [-0.04, 0.13] |  |  |

### Table S6i: Multinomial Logistic Regression Models using the predictor Disembodiment (11D-ASC)

| **Model** | **Reference Category** | **Outcome Category** | **Coefficient (B)** | **Standard Error (SE)** | **z-value** | **p-value** | **95% CI** | **Model Fit** | |
| --- | --- | --- | --- | --- | --- | --- | --- | --- | --- |
|  |  |  |  |  |  |  |  | **Log-Likelihood** | **McFadden's R²** |
| **1** | **Sustained** | Relapsing | -0.46 | 0.81 | -0.57 | 0.5707 | [-2.04, 1.13] | **0.06** | **1.00** |
|  |  | Non-response | -2.86 | 3.91 | -0.73 | 0.4647 | [-10.52, 4.81] |  |  |
| **2** | **Relapsing** | Sustained | 0.66 | 2.71 | 0.24 | 0.8087 | [-4.65, 5.97] | **0.03** | **1.00** |
|  |  | Non-response | -2.59 | 4.48 | -0.58 | 0.5625 | [-11.38, 6.19] |  |  |
| **3** | **Non-response** | Sustained | 2.24 | 3.89 | 0.58 | 0.5643 | [-5.39, 9.87] | **0.21** | **0.99** |
|  |  | Relapsing | 1.64 | 2.01 | 0.82 | 0.4125 | [-697.29, 467.89] |  |  |

### Table S6j: Multinomial Logistic Regression Models using the predictor Impaired Control and Cognition (11D-ASC)

| **Model** | **Reference Category** | **Outcome Category** | **Coefficient (B)** | **Standard Error (SE)** | **z-value** | **p-value** | **95% CI** | **Model Fit** | |
| --- | --- | --- | --- | --- | --- | --- | --- | --- | --- |
|  |  |  |  |  |  |  |  | **Log-Likelihood** | **McFadden's R²** |
| **1** | **Sustained** | Relapsing | -0.02 | 0.06 | -0.29 | 0.7680 | [-0.14, 0.10] | **13.53** | **0.10** |
|  |  | Non-response | -0.06 | 0.06 | -0.99 | 0.3237 | [-0.18, 0.06] |  |  |
| **2** | **Relapsing** | Sustained | 0.02 | 0.06 | 0.29 | 0.7693 | [-0.10, 0.14] | **13.53** | **0.10** |
|  |  | Non-response | -0.04 | 0.05 | -0.94 | 0.3492 | [-0.13, 0.05] |  |  |
| **3** | **Non-response** | Sustained | 0.06 | 0.06 | 0.99 | 0.3240 | [-0.06, 0.18] | **13.53** | **0.10** |
|  |  | Relapsing | 0.04 | 0.05 | 0.94 | 0.3497 | [-0.05, 0.13] |  |  |

### Table S6k: Multinomial Logistic Regression Models using the predictor Anxiety (11D-ASC)

| **Model** | **Reference Category** | **Outcome Category** | **Coefficient (B)** | **Standard Error (SE)** | **z-value** | **p-value** | **95% CI** | **Model Fit** | |
| --- | --- | --- | --- | --- | --- | --- | --- | --- | --- |
|  |  |  |  |  |  |  |  | **Log-Likelihood** | **McFadden's R²** |
| **1** | **Sustained** | Relapsing | 0.02 | 0.05 | 0.47 | 0.6354 | [-0.08, 0.13] | **14.63** | **0.03** |
|  |  | Non-response | -0.01 | 0.06 | -0.16 | 0.8732 | [-0.11, 0.10] |  |  |
| **2** | **Relapsing** | Sustained | -0.02 | 0.05 | -0.47 | 0.6353 | [-0.13, 0.08] | **14.63** | **0.03** |
|  |  | Non-response | -0.03 | 0.05 | -0.63 | 0.5273 | [-0.14, 0.07] |  |  |
| **3** | **Non-response** | Sustained | 0.01 | 0.06 | 0.16 | 0.8731 | [-0.10, 0.12] | **14.63** | **0.03** |
|  |  | Relapsing | 0.03 | 0.05 | 0.63 | 0.5274 | [-0.07, 0.14] |  |  |

### Table S6l: Multinomial Logistic Regression Models using the predictor Complex Imagery (11D-ASC)

| **Model** | **Reference Category** | **Outcome Category** | **Coefficient (B)** | **Standard Error (SE)** | **z-value** | **p-value** | **95% CI** | **Model Fit** | |
| --- | --- | --- | --- | --- | --- | --- | --- | --- | --- |
|  |  |  |  |  |  |  |  | **Log-Likelihood** | **McFadden's R²** |
| **1** | **Sustained** | Relapsing | 0.00 | 0.03 | 0.08 | 0.9381 | [-0.05, 0.06] | **15.03** | **<0.01** |
|  |  | Non-response | -0.01 | 0.03 | -0.18 | 0.8595 | [-0.07, 0.06] |  |  |
| **2** | **Relapsing** | Sustained | -0.00 | 0.03 | -0.08 | 0.9381 | [-0.06, 0.05] | **15.03** | **<0.01** |
|  |  | Non-response | -0.01 | 0.03 | -0.27 | 0.7862 | [-0.06, 0.05] |  |  |
| **3** | **Non-response** | Sustained | 0.01 | 0.03 | 0.18 | 0.8594 | [-0.06, 0.07] | **15.03** | **<0.01** |
|  |  | Relapsing | 0.01 | 0.03 | 0.27 | 0.7861 | [-0.05, 0.06] |  |  |

### Table S6m: Multinomial Logistic Regression Models using the predictor Elemental Imagery (11D-ASC)

| **Model** | **Reference Category** | **Outcome Category** | **Coefficient (B)** | **Standard Error (SE)** | **z-value** | **p-value** | **95% CI** | **Model Fit** | |
| --- | --- | --- | --- | --- | --- | --- | --- | --- | --- |
|  |  |  |  |  |  |  |  | **Log-Likelihood** | **McFadden's R²** |
| **1** | **Sustained** | Relapsing | -0.11 | 0.10 | -1.11 | 0.2660 | [-0.31, 0.09] | **4.26** | **0.72** |
|  |  | Non-response | -0.54 | 1.32 | -0.41 | 0.6812 | [-3.12, 2.04] |  |  |
| **2** | **Relapsing** | Sustained | 0.11 | 0.10 | 1.11 | 0.2658 | [-0.08, 0.31] | **4.26** | **0.72** |
|  |  | Non-response | -0.46 | 1.77 | -0.26 | 0.7959 | [-3.93, 3.02] |  |  |
| **3** | **Non-response** | Sustained | 0.48 | 0.84 | 0.57 | 0.5682 | [-1.17, 2.13] | **4.27** | **0.72** |
|  |  | Relapsing | 0.37 | 0.84 | 0.44 | 0.6593 | [-1.27, 2.01] |  |  |

### Table S6n: Multinomial Logistic Regression Models using the predictor Audio-Visual Synaesthesia (11D-ASC)

| **Model** | **Reference Category** | **Outcome Category** | **Coefficient (B)** | **Standard Error (SE)** | **z-value** | **p-value** | **95% CI** | **Model Fit** | |
| --- | --- | --- | --- | --- | --- | --- | --- | --- | --- |
|  |  |  |  |  |  |  |  | **Log-Likelihood** | **McFadden's R²** |
| **1** | **Sustained** | Relapsing | -0.08 | 0.08 | -0.96 | 0.3386 | [-0.24, 0.08] | **9.92** | **0.34** |
|  |  | Non-response | -0.13 | 0.09 | -1.37 | 0.1711 | [-0.31, 0.06] |  |  |
| **2** | **Relapsing** | Sustained | 0.08 | 0.08 | 0.96 | 0.3387 | [-0.08, 0.24] | **9.92** | **0.34** |
|  |  | Non-response | -0.05 | 0.05 | -1.11 | 0.2668 | [-0.14, 0.04] |  |  |
| **3** | **Non-response** | Sustained | 0.13 | 0.09 | 1.37 | 0.1709 | [-0.06, 0.31] | **9.92** | **0.34** |
|  |  | Relapsing | 0.05 | 0.05 | 1.11 | 0.2666 | [-0.04, 0.14] |  |  |

### Table S6o: Multinomial Logistic Regression Models using the predictor Changed Meaning of Percepts (11D-ASC)

| **Model** | **Reference Category** | **Outcome Category** | **Coefficient (B)** | **Standard Error (SE)** | **z-value** | **p-value** | **95% CI** | **Model Fit** | |
| --- | --- | --- | --- | --- | --- | --- | --- | --- | --- |
|  |  |  |  |  |  |  |  | **Log-Likelihood** | **McFadden's R²** |
| **1** | **Sustained** | Relapsing | -0.06 | 0.05 | -1.13 | 0.2581 | [-0.16, 0.04] | **4.58** | **0.70** |
|  |  | Non-response | -1.70 | 57.64 | -0.03 | 0.9765 | [-114.67, 111.27] |  |  |
| **2** | **Relapsing** | Sustained | 0.06 | 0.05 | 1.13 | 0.2580 | [-0.04, 0.16] | **4.58** | **0.70** |
|  |  | Non-response | -1.33 | 19.41 | -0.07 | 0.9454 | [-39.38, 36.72] |  |  |
| **3** | **Non-response** | Sustained | 4.67 | 0.03 | 178.28 | **<0.0001** | [4.62, 4.73] | **4.58** | **0.70** |
|  |  | Relapsing | 4.62 | 0.03 | 176.02 | **<0.0001** | [4.56, 4.66] |  |  |

### Table S6p: Multinomial Logistic Regression Models using the predictor PIQ

| **Model** | **Reference Category** | **Outcome Category** | **Coefficient (B)** | **Standard Error (SE)** | **z-value** | **p-value** | **95% CI** | **Model Fit** | |
| --- | --- | --- | --- | --- | --- | --- | --- | --- | --- |
|  |  |  |  |  |  |  |  | **Log-Likelihood** | **McFadden's R²** |
| **1** | **Sustained** | Relapsing | -0.07 | 0.07 | -1.09 | 0.2770 | [-0.21, 0.06] | **12.23** | **0.19** |
|  |  | Non-response | -0.06 | 0.07 | -0.90 | 0.3682 | [-0.19, 0.07] |  |  |
| **2** | **Relapsing** | Sustained | 0.07 | 0.07 | 1.09 | 0.2769 | [-0.06, 0.21] | **12.23** | **0.19** |
|  |  | Non-response | 0.01 | 0.03 | 0.50 | 0.6138 | [-0.04, 0.06] |  |  |
| **3** | **Non-response** | Sustained | 0.06 | 0.07 | 0.90 | 0.3687 | [-0.07, 0.19] | **12.23** | **0.19** |
|  |  | Relapsing | -0.01 | 0.03 | -0.50 | 0.6141 | [-0.06, 0.04] |  |  |

### Table S6q: Multinomial Logistic Regression Models using the predictor EBI

| **Model** | **Reference Category** | **Outcome Category** | **Coefficient (B)** | **Standard Error (SE)** | **z-value** | **p-value** | **95% CI** | **Model Fit** | |
| --- | --- | --- | --- | --- | --- | --- | --- | --- | --- |
|  |  |  |  |  |  |  |  | **Log-Likelihood** | **McFadden's R²** |
| **1** | **Sustained** | Relapsing | -0.27 | 0.35 | -0.78 | 0.4358 | [-0.95, 0.41] | **10.19** | **0.33** |
|  |  | Non-response | -0.29 | 0.35 | -0.83 | 0.4084 | [-0.97, 0.39] |  |  |
| **2** | **Relapsing** | Sustained | 0.30 | 0.41 | 0.72 | 0.4704 | [-0.51, 1.11] | **10.19** | **0.33** |
|  |  | Non-response | -0.02 | 0.04 | -0.40 | 0.6879 | [-0.10, 0.07] |  |  |
| **3** | **Non-response** | Sustained | 0.29 | 0.34 | 0.84 | 0.4031 | [-0.38, 0.95] | **10.20** | **0.33** |
|  |  | Relapsing | 0.02 | 0.04 | 0.41 | 0.6827 | [-0.07, 0.10] |  |  |

# Feasibility and participant safety and wellbeing

Participants were discharged between 6 and 7.5 hours after psilocybin administration. There were 6 cases (n = 4) of blood pressure and 1 case of heart rate exceeding threshold for further monitoring during dosing sessions (160/110 mmHg, 110 bpm), all resolved spontaneously without intervention. No participant required rescue medication during the dosing sessions or used the 24-hour therapist support phone line. There were no clinically significant changes in safety blood tests post-dosing, and no symptoms of HPPD reported.

## Table S7: Adverse events during screening

|  | **Screening phase** | **Severity** | **Seriousness** | **Resolved?** |
| --- | --- | --- | --- | --- |
| **Privacy concern** Request for deletion | Screening survey | Mild | Not serious | Yes |
| **Privacy concern** Request for deletion | Screening survey | Mild | Not serious | Yes |
| **Depression exacerbated** Deterioration during antidepressant withdrawal | Healthcare consultation and antidepressant withdrawal | Moderate | Not serious | Yes |
| **Unspecified adverse reaction to externally prescribed Delta-9-tetrahydrocannabinol (THC)** Participant no longer wished to participate due to this experience | Healthcare consultation and antidepressant withdrawal | Moderate | Not serious | Yes |

## Table S8: Adverse events by participant reported after enrolment

|  | **Related to pre-existing condition** | **Severity** | **Relationship to study drug** | **Starting timepoint** | **Duration** |
| --- | --- | --- | --- | --- | --- |
| **PARTICIPANT 1** |  |  |  |  |  |
| **Depression exacerbation** Linked with medication withdrawal | Yes – depression | Mild | No | Baseline | 4 days |
| **Sleep myoclonus exacerbated** Increased myoclonus intensity/frequency leading to insomnia. Linked with medication withdrawal. | Yes – sleep myoclonus | Moderate | Possibly | PPT2 | Unresolved Probable cause: pre-existing sleep myoclonus that fluctuates with medications and psychedelic use. Onset linked to previous psychedelic use. |
| **Insomnia exacerbated** Interrupted asleep and poor sleep quality. Linked to sleep myoclonus exacerbation and medication withdrawal. | Yes – insomnia, restless leg, sleep myoclonus | Moderate | Possibly | PPT2 | Unresolved Probable cause: pre-existing sleep myoclonus that fluctuates with mood, medications, and psychedelic use |
| **Nausea**  No vomitting | No | Mild | Probably | Dose 1 | 2 hours |
| **Nausea** No vomitting | No | Moderate | Probably | Dose 2 | 2 hours |
| **Depression exacerbation**  Return of previous symptoms | Yes – depression | Moderate | No | Long-term follow-up | Unresolved  Probable cause: return of pre-existing depression |
| **PARTICIPANT 2** |  |  |  |  |  |
| **Urinary incontinence** | No | Moderate | Probably | Dose 1 | 1 event |
| **Anxiety** Medication prescribed but issue persisted**.** | Yes – depression | Moderate-Severe | Probably | IPT2 | Unresolved  Probable cause: pre-existing psychological challenges and psilocybin dosing |
| **Suicidal ideation exacerbation**  Passive | Yes – depression | Moderate | Probably | 2 days post dose 1 | 12 days |
| **Nausea**  Persistent low-level nausea, no vomitting | No | Moderate | Probably | IPT2 | 12 days |
| **Decreased appetite**  As a result of nausea, resulting in reduced food intake and self-reported weight loss of 9 kg | No | Moderate | Probably | IPT2 | 65 days |
|  |  |  |  |  |  |
| **Insomnia**  Difficulty falling asleep and early morning awakenings. Improved with medication. | No | Moderate | Probably | IPT2 | 65 days |
| **Inappropriate behaviour**  Removal of clothing and refusal to redress in dosing room | No | Mild | Probably | Dose 2 | 45 minutes |
| **Inappropriate behaviour**  Attempt to leave treatment room in an agitated state. Refusal to leave bathroom and return to treatment room. | No | Mild | Probably | Dose 2 | 1 event |
| **PARTICIPANT 3** |  |  |  |  |  |
| **Headache** | No | Mild | Probably | Dose 1 | 1 day |
| **PARTICIPANT 4** |  |  |  |  |  |
| **Abnormal perception**  Strong scent perceived in the absense of odors. Fluctuated with music - auditory-olfactory syneasthesia. | Yes – general hypersensitivity to scent | Mild | Probably | Dose 1 | 1 day |
| **Nausea**  No vomitting | No | Mild | Probably | Dose 1 | 15 days |
| **Depression exacerbation**  Return of previous symptoms | Yes – depression | Moderate | No | Long-term follow-up | Unresolved  Probable cause: return of pre-existing depression |
| **PARTICIPANT 5** |  |  |  |  |  |
| **Insomnia**  Linked to medication withdrawal. Worsened following dosing session. | Yes – depression and poor sleep | Moderate | No | Baseline | Unresolved Probable cause: pre-existing depression with poor sleep |
| **Depression exacerbation**  Linked to insomnia and medication withdrawal | Yes – depression | Mild | No | Baseline | 2 weeks |
| **Herpes zoster**  (Shingles) | No | Moderate | No | PPT2 | 3 weeks |
| **Nausea**  No vomitting | No | Moderate | Probably | Dose 1 | 1 day |
| **PARTICIPANT 6** |  |  |  |  |  |
| **Upper respiratory tract infection** | No | Mild | No | IPT4 | 3 days |
| **PARTICIPANT 7** |  |  |  |  |  |
| **Headache** | No | Mild | Probably | Dose 1 | 2 days |
| **Nausea** | No | Mild | Probably | Dose 2 | 1 day |
| **Headache** Repeated minor headaches, no OTC pain relief needed | No | Mild | No | 3W post-dose 2 | 1 week |
| **Depression exacerbation**  Return of previous symptoms | Yes – depression | Moderate | No | Long-term follow-up | Unresolved - cause identified: return of pre-existing depression |

PPT = Preparatory psychotherapy. IPT = Integrative psychotherapy. No adverse events recorded for participant 8 who was withdrawn prior to starting treatment

##

## Protocol deviations

One participant initially began the trial but did not proceed to dosing due to an issue with therapist continuity. The participant was later re-enrolled with a different therapist team and completed the full protocol. Only data from the completed participation were included in the analyses.

One participant started taking an ADHD medication prior to the end of the treatment phase, between IPT5 and IPT6. This was not disclosed to trial staff until after these sessions and the 3W post-dose 2 assessments had been conducted.

One participant recommenced antidepressant medication at IPT5 as recommended by the therapist dyad and external therapist.

One participant completed the 1D post-dose 2 assessments outside of the required window in the protocol due to sickness (unrelated to dosing). They completed this assessment 3 days after the second dose.

# REFERENCES

Hothorn T, Hornik K, Van De Wiel MA, et al. (2006) A lego system for conditional inference. *The American Statistician* 60(3): 257-263.

Jackman S (2010) pscl: Classes and methods for r. developed in the political science computational laboratory, stanford university. department of political science, stanford university, stanford, ca. r package version 1.03. 5. [*http://www*](http://www)*. pscl. stanford. edu/*.

Torchiano M (2020) effsize: Efficient effect size computation. *R package version 0.8* 1(10.5281).

Venables W (2002) Statistics complements to modern applied statistics with S Fourth edition. *(No Title)*.
